# Geographic and sociodemographic variation of cardiovascular disease risk in India: A cross-sectional study of 797,540 adults

**DOI:** 10.1371/journal.pmed.1002581

**Published:** 2018-06-19

**Authors:** Pascal Geldsetzer, Jennifer Manne-Goehler, Michaela Theilmann, Justine I. Davies, Ashish Awasthi, Goodarz Danaei, Thomas A. Gaziano, Sebastian Vollmer, Lindsay M. Jaacks, Till Bärnighausen, Rifat Atun

**Affiliations:** 1 Department of Global Health and Population, Harvard T.H. Chan School of Public Health, Harvard University, Boston, Massachusetts, United States of America; 2 Department of Medicine, Beth Israel Deaconess Medical Center, Harvard Medical School, Boston, Massachusetts, United States of America; 3 Department of Economics, University of Goettingen, Göttingen, Germany; 4 Centre for Modern Indian Studies, University of Goettingen, Göttingen, Germany; 5 MRC/Wits Rural Public Health and Health Transitions Research Unit, School of Public Health, University of the Witwatersrand, Johannesburg, South Africa; 6 Centre for Global Health, King’s College London, London, United Kingdom; 7 Indian Institute of Public Health, Gandhinagar, Gujarat, India; 8 Department of Epidemiology, Harvard T.H. Chan School of Public Health, Harvard University, Boston, Massachusetts, United States of America; 9 Department of Cardiovascular Medicine, Brigham and Women’s Hospital, Harvard Medical School, Boston, Massachusetts, United States of America; 10 Center for Health Decision Science, Harvard T.H. Chan School of Public Health, Boston, Massachusetts, United States of America; 11 Public Health Foundation of India, Delhi, National Capital Region, India; 12 Institute of Public Health, Heidelberg University, Heidelberg, Germany; 13 Africa Health Research Institute, Mtubatuba, South Africa; 14 Department of Global Health and Social Medicine, Harvard Medical School, Harvard University, Boston, Massachusetts, United States of America; The George Institute for Global Health, UNSW Sydney, AUSTRALIA

## Abstract

**Background:**

Cardiovascular disease (CVD) is the leading cause of mortality in India. Yet, evidence on the CVD risk of India’s population is limited. To inform health system planning and effective targeting of interventions, this study aimed to determine how CVD risk—and the factors that determine risk—varies among states in India, by rural–urban location, and by individual-level sociodemographic characteristics.

**Methods and findings:**

We used 2 large household surveys carried out between 2012 and 2014, which included a sample of 797,540 adults aged 30 to 74 years across India. The main outcome variable was the predicted 10-year risk of a CVD event as calculated with the Framingham risk score. The Harvard–NHANES, Globorisk, and WHO–ISH scores were used in secondary analyses. CVD risk and the prevalence of CVD risk factors were examined by state, rural–urban residence, age, sex, household wealth, and education. Mean CVD risk varied from 13.2% (95% CI: 12.7%–13.6%) in Jharkhand to 19.5% (95% CI: 19.1%–19.9%) in Kerala. CVD risk tended to be highest in North, Northeast, and South India. District-level wealth quintile (based on median household wealth in a district) and urbanization were both positively associated with CVD risk. Similarly, household wealth quintile and living in an urban area were positively associated with CVD risk among both sexes, but the associations were stronger among women than men. Smoking was more prevalent in poorer household wealth quintiles and in rural areas, whereas body mass index, high blood glucose, and systolic blood pressure were positively associated with household wealth and urban location. Men had a substantially higher (age-standardized) smoking prevalence (26.2% [95% CI: 25.7%–26.7%] versus 1.8% [95% CI: 1.7%–1.9%]) and mean systolic blood pressure (126.9 mm Hg [95% CI: 126.7–127.1] versus 124.3 mm Hg [95% CI: 124.1–124.5]) than women. Important limitations of this analysis are the high proportion of missing values (27.1%) in the main outcome variable, assessment of diabetes through a 1-time capillary blood glucose measurement, and the inability to exclude participants with a current or previous CVD event.

**Conclusions:**

This study identified substantial variation in CVD risk among states and sociodemographic groups in India—findings that can facilitate effective targeting of CVD programs to those most at risk and most in need. While the CVD risk scores used have not been validated in South Asian populations, the patterns of variation in CVD risk among the Indian population were similar across all 4 risk scoring systems.

## Abstract 2

In a large observational study, Rifat Atun and colleagues study cardiovascular risk factors in different regions and populations across India.

## Author summary

**Why was this study done?:**

- Cardiovascular disease (CVD) is thought to cause a large and increasing health and economic burden in India.
- Understanding how CVD risk varies among India’s population groups could inform health system planning and the targeting of CVD programs to those most in need.
- Yet, to date, there has not, to our knowledge, been a large-scale population-based study that examines how CVD risk varies among India’s states and sociodemographic groups.

**What did the researchers do and find?:**

- This analysis pooled data from 797,540 participants aged 30 to 74 years across 2 large population-based household surveys, which jointly covered 27 of 29 states and 5 of 7 union territories in India.
- The average 10-year risk of a fatal or nonfatal CVD event varied widely among states in India, ranging from 13.2% in Jharkhand to 19.5% in Kerala.
- In addition, adults living in urban areas, as well as those with a higher household wealth or education, tended to have a greater CVD risk.

**What do these findings mean?:**

- This study identified important variation in composite CVD risk (as well as in the prevalence of individual CVD risk factors) among India’s population, which can inform effective targeting of CVD programs to those most at risk and most in need.
- While the absolute CVD risk levels predicted by any 1 of the 4 CVD risk calculators we used should be interpreted with caution (because of the absence of CVD risk equations that have been validated among South Asian populations), the relative variation of CVD risk among India’s population groups was similar across all 4 risk calculators.

## Introduction

Cardiovascular disease (CVD) is the leading cause of mortality worldwide, including in low- and middle-income countries [1]. While the Global Burden of Disease project has recently highlighted the limited data availability for India [2], it nonetheless estimated that the country contributed almost one-fifth (18.6%) of the global CVD burden, as measured by disability-adjusted life years, in 2016 [3]. Although this proportion is only slightly above the share of the world’s population that lives in India (17.7% in 2015) [4], it is likely to increase in the future for 3 main reasons. First, India is expected to make the greatest contribution to global population growth of any country until at least 2050 [5]. Second, India’s population is aging and urbanizing: the share of people aged more than 60 years is estimated to double from 8.9% to 19.4% between 2015 and 2050 [5], and the percentage of Indians living in cities is projected to grow from 30.9% in 2010 to 50.3% in 2050 [6]. Third, the rise in living standards and socio-cultural transitions in India are likely to lead to more obesogenic lifestyles [7]. Evidence indicates that urban South Asians, especially those living in North America and Western Europe, have a higher prevalence of CVD and type 2 diabetes than local white populations [8–10]. While the reasons for this phenomenon are not clear (although some explanatory models have been proposed in the literature) [10,11], this susceptibility for CVD among South Asians living in urban, high-income settings suggests that increasing urbanization and the spread of obesogenic environments might raise the prevalence of CVD even more in India (and South Asia in general) than it has already in other world regions.

Given the detrimental effects of CVD on health outcomes [12], financial risk protection [13], and economic growth [14], the course of India’s CVD epidemic will directly impact several Sustainable Development Goals (SDGs). These include SDG 1 (“End poverty in all its forms everywhere”) and SDG 3 (“Ensure healthy lives and promote well-being for all at all ages”) as well as their corresponding targets SDG 3.4 (“By 2030, reduce by one-third premature mortality from NCDs [noncommunicable diseases]”) and SDG 3.8 on achieving universal health coverage. Considering the size and growth of India’s population [5], the development of its CVD epidemic over the next decade will also have a decisive impact on the world’s ability to achieve the SDGs [15].

Many studies have focused on providing the best possible prevalence estimates for CVD and its risk factors at the national level in India [16–19]. However, much less is known about the distribution of these risk factors within India—both geographically and by individuals’ sociodemographic characteristics. Given that India’s health system is largely decentralized to the state level [20], understanding the variation of CVD risk within India is highly relevant not only to identify target groups for CVD prevention, screening, and treatment programs but also for health system planning at the state and district level. Using data from a sample of 797,540 adults aged 30–74 years, this study therefore aimed to determine how CVD risk varies by geography and individual-level sociodemographic characteristics across India.

## Methods

### Data sources

We pooled data from 2 large household surveys in India, the District Level Household Survey–4 (DLHS-4) and the second update of the Annual Health Survey (AHS), both of which were conducted between 2012 and 2014. These 2 surveys were combined because they (i) jointly covered most states (27 of 29) and union territories (5 of 7) of India (and no areas in India were covered by both surveys), (ii) were conducted simultaneously, (iii) are both representative at the district level, and (iv) used the same questionnaire and methodology to collect clinical, anthropometric, and biomarker (CAB) measurements. The states covered by each of the surveys are shown in Fig A in S1 Fig.

In both surveys, all non-pregnant household members aged 18 years and older were eligible for blood glucose, blood pressure (BP), height, and weight measurements. The analyses in this study were restricted to those aged 30 to 74 years because the CVD risk equations used in this study were developed among adults of this age range only [21–23]. Body mass index (BMI) was calculated as weight in kilograms divided by the square of height in meters. Participants’ blood glucose was measured using a capillary blood sample (from a finger prick) taken using a handheld blood glucose meter (SD CodeFree), which multiplied capillary blood glucose readings by 1.11 to display their plasma equivalent [24]. Participants were instructed to fast for at least 8 hours before the time of the measurement. BP was measured twice, with each measurement 10 minutes apart, using an electronic upper arm BP monitor (Rossmax AW150).

All data collectors for the AHS and DLHS-4 were trained in the collection of sociodemographic as well as the CAB data. In the AHS and DLHS-4, training sessions were organized for 12–15 and 15–20 data collectors at a time, respectively. Trainings for anthropometric and biomarker measurements lasted for 7 days, with 4 days of training conducted in the classroom and 3 days in the field. The following mechanisms were put in place for both the AHS and DLHS-4 to ensure good data quality: (i) establishment of standard protocols for questionnaire administration, anthropometry, BP measurement, and blood glucose measurement, (ii) the field supervisor conducted a second CAB measurement on 10% of participants each day to identify poor-quality measurements, (iii) a medical consultant (who received additional training for the CAB component) visited 10% of all sampled households and conducted a second CAB assessment to identify poor-quality measurements, (iv) continuous data monitoring by the implementing organization, (v) immediate replacement of faulty equipment, and (vi) regular checks of the accuracy of digital BP monitors and the handheld blood glucometers. More details on the data collection procedures can be found in the CAB manuals of the AHS and DLHS-4 [25,26]. The documents can be obtained from the corresponding author.

### Sampling procedure

The AHS and DLHS-4 jointly cover all 29 states of India apart from Jammu and Kashmir (where data were not collected due to violent conflicts) and Gujarat (where data were not available in the public domain). The datasets also include all union territories of India except Dadra and Nagar Haveli, and Lakshadweep. The 2 states and 2 union territories not included in this analysis accounted for 6% of India’s population at the time of the last census (2011) [27].

#### Annual health survey

Carried out between 2012 and 2013, the AHS covered all 284 districts in 9 states of India that were selected for the AHS because they had the highest rate of infant and child mortality in the country in 2010 [28]. These states accounted for 48% of the country’s population in 2011 [27]. The AHS employed a self-weighting 2-stage cluster random sampling design (stratified by rural versus urban) in each district, whereby primary sampling units (PSUs) were villages in rural areas and census enumeration blocks in urban areas. Secondary sampling units (SSUs) were households. PSUs were selected through simple random sampling with probability proportional to population size (using projections from the 2001 India Census). After all households in a PSU were enumerated, households were selected using systematic random sampling (with an interval of 2) whereby the first household in each PSU was selected randomly, and then every alternate (third, fifth, seventh, etc.) household was selected, for blood glucose, BP, height, and weight measurements. These measurements were taken 12 to 18 months after administration of a questionnaire, which asked about the same participants’ sociodemographic information, including treatment for diabetes and hypertension as well as smoking history. Thus, the sociodemographic and CAB information were collected at 2 different time-points, and both were only collected once. We merged the dataset containing participants’ sociodemographic information with the dataset containing their anthropometric, BP, and blood glucose measurements as described in Text A in S1 Text.

#### District level household survey–4

Carried out between 2012 and 2014, the DLHS-4 covered all 336 districts in 18 states and 5 union territories (henceforth also referred to as “states”) of India, which jointly accounted for 46% of India’s population at the time of the 2011 census [27,28]. The DLHS-4 used 2-stage cluster random sampling (stratified by urban versus rural). PSUs were “census villages” in rural areas and “urban frame survey blocks” in urban areas; SSUs were households. Rural PSUs were selected with probability proportional to population size, and urban PSUs through simple random sampling. SSUs were selected using systematic random sampling. A more detailed description of the sampling procedure is available in the DLHS-4 state reports [29].

### Ethics

This analysis of an existing dataset in the public domain received a determination of “not human subjects research” by the institutional review board of the Harvard T.H. Chan School of Public Health on 23 November 2016 (protocol number: IRB16-1915). All participants provided written informed consent to participate in the AHS and DLHS-4.

### Outcome variables

Throughout this analysis, we used the predicted 10-year risk of a CVD event to summarize CVD risk as computed by risk calculators across different risk factors. However, we also “disaggregated” predicted CVD risk by examining the geographic and sociodemographic variation of each of the risk factors included in these risk calculators: (i) BMI, (ii) high blood glucose, (iii) systolic BP, and (iv) smoking. Results on diastolic BP are presented in supplementary files for completeness (Figs D and G in S1 Fig).

We primarily used continuous predicted 10-year CVD risk as an outcome. However, in secondary analyses, we dichotomized predicted 10-year risk of a CVD event into high and low risk whereby “high CVD risk” was defined as a 10-year CVD risk ≥ 30%. This threshold was chosen because it is the cutoff used in the World Health Organization’s NCD Global Action Plan targets to decide who is eligible for drug therapy and counseling [30]. We primarily used the Framingham risk score (the version not requiring total cholesterol measurements) to calculate CVD risk because it is the most widely used CVD risk scoring system internationally [21,31]. However, in secondary analyses, we also show results using CVD risk calculated with 3 other risk scores that do not require blood lipid measurements, namely Harvard–NHANES [23], Globorisk [32], and the risk score developed by WHO and the International Society for Hypertension (WHO–ISH) [33]. None of these risk scores have been validated among South Asian populations. Because data on participants’ medical history were unavailable, we did not exclude participants with a previous or current CVD.

All 4 risk scores used predict the risk of a fatal or nonfatal CVD event, but each score defines a CVD event differently (Table 1). The Framingham risk score uses the broadest [21], and Globorisk [32] and WHO–ISH [33] the narrowest, range of CVD events as outcome. The Globorisk project has calibrated its risk equation to 182 countries, including India, as described by Ueda et al. [32]. Similarly, WHO has calibrated its risk score to each WHO sub-region [33]. The Framingham and Harvard–NHANES risk scores were calibrated to India using the incidence rate (by 5-year age group) of peripheral artery disease (Framingham only), ischemic heart disease, and cerebrovascular disease in 2015 as estimated by the Global Burden of Disease project [12].

**Table 1:** Outcomes predicted by each cardiovascular risk score.

Outcome category	Framingham [21]	Harvard–NHANES [23]	Globorisk [32]	WHO–ISH [33]
*Fatal outcomes*	• Any coronary disease • Stroke	• Any coronary disease • Stroke	• Myocardial infarction • Stroke • Sudden cardiac death	• Myocardial infarction • Stroke
*Nonfatal outcomes*	• Angina pectoris • Coronary insufficiency • Heart failure • Myocardial infarction • Peripheral artery disease • Stroke • Transient ischemic attack	• Congestive heart failure • Coronary revascularization • Myocardial infarction • Stroke	• Myocardial infarction • Stroke	• Myocardial infarction • Stroke

The 4 risk scores predict CVD risk by sex using the following inputs: age, BMI (except WHO–ISH), presence of diabetes (except the office-based version of the Globorisk score), current smoking, systolic BP, and treatment for hypertension (except Globorisk and WHO–ISH). Diabetes was defined as having a blood glucose ≥7.0 mmol/l if reporting to have fasted or ≥11.1 mmol/l if reporting not to have fasted, or reporting to be on regular treatment for diabetes. Because the survey only measured blood glucose to assess diabetes, which is insufficient for a clinical diagnosis of this condition, we refer to this outcome as “high blood glucose” for the remainder of the paper. For systolic BP, we used the average of the 2 systolic BP readings recorded.

### Explanatory variables

Explanatory variables were household wealth quintile, education, and whether the household was located in a rural or urban area. We used a principal component analysis to create a household wealth index based on 5 key housing characteristics (water supply, type of toilet and whether it is shared, cooking fuel, housing material, and source of lighting) and household ownership of 12 assets (radio, TV, computer, phone, refrigerator, bicycle, scooter, car, washing machine, sewing machine, house, and land). The first component in the principal component analysis (using the methodology developed by Filmer and Pritchett [34,35]) was used to combine these variables into a single measure, separately for urban and rural areas. This index was then divided into quintiles (again, separately for rural and urban areas) based on the distribution in the national (aggregate) dataset.

### Statistical analysis

CVD risk was computed for each study participant aged 30 to 74 years. Using sampling weights to account for the complex survey design, we then calculated the mean 10-year CVD risk at the national level, by state, and by individual-level sociodemographic characteristics. All mean risk values (and prevalence estimates) are unadjusted for individuals’ sociodemographic characteristics (other than age standardization where explicitly indicated). In addition, we used ordinary least squares regressions to regress the natural logarithm of the CVD risk score on sociodemographic characteristics and a fixed effect for district (i.e., a binary indicator for each district to adjust for unobserved differences between districts). The natural logarithm of CVD risk was used in all regression models to allow for a more intuitive interpretation of the regression coefficients as percentage changes in CVD risk. The regressions were run separately for males and females because each CVD risk score provides sex-specific risks. Two different regression models were fitted for each CVD risk score (except WHO–ISH because it only provides risk categories rather than a continuous risk variable [33]) and sex: (i) a model that included only 1 sociodemographic characteristic, age group, and a district-level fixed effect and (ii) a model that included all sociodemographic characteristics and a district-level fixed effect as explanatory variables. Standard errors were adjusted for clustering at the level of the PSU. The mean (for BMI and systolic BP) or the prevalence (for high blood glucose and smoking) of each CVD risk factor was plotted by state and sociodemographic characteristics to help explain observed patterns in the CVD risk scores.

This study did not have a prospective analysis plan. The analysis outlined above was conceived by the authors prior to embarking on data analysis. None of the analyses were unplanned with the exception that reviewer comments led us to add (i) additional maps to examine state-level variation (specifically, to stratify variation not only by sex but also by age group and rural–urban residence) and (ii) multi-level modeling to examine the association of CVD risk with district-level wealth and urbanization. Regarding the latter, the peer reviewer comments prompted us to further investigate area-level predictors of CVD risk because we identified wide geographic variation in CVD risk in our initial analysis. To do so, we computed a measure of district-level wealth by calculating (separately for rural and urban areas within districts because household wealth was also computed separately for rural and urban areas) the median of the continuous household wealth index in a district, and then categorizing the district-level median into quintiles (henceforth referred as “district wealth quintiles”). Another potential area-level predictor that we examined was the level of urbanization of a district assessed through the proportion of participants in a district who were residing in an urban area. These 2 area-level predictors were chosen because they could be calculated directly from the data. We, thus, did not have to rely on the accuracy of other data sources, and—unlike other indicators—these district-level indicators were automatically available for all districts in the sample for the time of the survey. The association of these 2 district-level predictors with CVD risk were studied using a multivariable linear regression model with the natural logarithm of 10-year CVD risk as the dependent variable, random intercepts by district, and individual-level sociodemographic characteristics (5-year age group, sex, educational attainment, and household wealth quintile) as independent variables.

We conducted a complete case analysis for all analyses presented in this paper. The Global Burden of Disease project’s 2013 population for India was used for age standardization [36]. This study is reported as per STROBE guidelines (S1 Checklist). Statistical analyses were run in R version 3.3.2 (2016) [37], and the WHO–ISH score was calculated using the whoishRisk package [38].

## Results

### Sample characteristics

Sociodemographic information was available for a total of 1,094,754 adults aged 30–74 years, which included individuals who were not present at the time of the household visit (as sociodemographic information was collected for all household members from the household head). In total, 797,540 (72.9% [797,540/1,094,754]) survey participants who had all the values for the variables needed to calculate each CVD risk score (i.e., blood glucose, systolic BP, height and weight, age, sex, and smoking status) were included in the analysis. While mean BMI was similar between males and females (22.6 kg/m^2^ and 22.3 kg/m^2^, respectively), females were more likely to have BMI < 18.5 kg/m^2^ or BMI ≥ 25 kg/m^2^ than males (Table 2). In all, 10.0% (42,066/420,691) of females and 10.7% (40,444/376,849) of males had high blood glucose. Smoking prevalence and mean systolic BP were higher among men than women (27.1% [102,182/376,849] versus 2.6% [10,992/420,691] and 129.1 mm Hg versus 126.7 mm Hg, respectively). In all, 56.2% (236,555/420,691) of females and 34.0% (128,183/376,849) of males had not completed primary school, and approximately one-third of participants lived in urban areas. Table A in S1 Table shows that those who were excluded from the analysis (27.1% of participants) because they had a missing value for at least 1 of the variables needed to calculate predicted CVD risk had a similar prevalence of CVD risk factors as those who were included in the analysis.

**Table 2:** Sample characteristics.

Characteristic	Females	Males
***n***	420,691	376,849
**Cardiovascular risk factors**		
Age group		
30–34 years	72,262 (17.2%)	57,874 (15.4%)
35–39 years	71,458 (17.0%)	56,575 (15.0%)
40–44 years	64,453 (15.3%)	55,851 (14.8%)
45–49 years	55,589 (13.2%)	50,610 (13.4%)
50–54 years	49,350 (11.7%)	44,312 (11.8%)
55–59 years	37,064 (8.8%)	36074 (9.6%)
60–64 years	31,893 (7.6%)	32,639 (8.7%)
65–69 years	23,553 (5.6%)	25,197 (6.7%)
70–74 years	15,069 (3.6%)	17,717 (4.7%)
*Missing*	*0 (0*.*0%)*	*0 (0*.*0%)*
Mean BMI in kg/m^2^ (SD)	22.6 (4.8)	22.3 (4.1)
*Missing*	*0 (0*.*0%)*	*0 (0*.*0%)*
BMI		
<18.5 kg/m^2^	72,882 (17.3%)	59,100 (15.7%)
18.5–22.9 kg/m^2^	183,441 (43.6%)	176,857 (46.9%)
23.0–24.9 kg/m^2^	63,412 (15.1%)	64,810 (17.2%)
25.0–29.9 kg/m^2^	74,037 (17.6%)	61,241 (16.3%)
≥30.0 kg/m^2^	26,919 (6.4%)	14,841 (3.9%)
High blood glucose^1^	42,066 (10.0%)	40,444 (10.7%)
*Missing*	*0 (0*.*0%)*	*0 (0*.*0%)*
Current smoking	10,992 (2.6%)	102,182 (27.1%)
*Missing*	*0 (0*.*0%)*	*0 (0*.*0%)*
Mean systolic BP in mm Hg (SD)	126.7 (21.3)	129.1 (19.7)
*Missing*	*0 (0*.*0%)*	*0 (0*.*0%)*
Systolic BP		
<120 mm Hg	168,890 (40.1%)	118,408 (31.4%)
120–129 mm Hg	93,055 (22.1%)	92,742 (24.6%)
130–139 mm Hg	66,204 (15.7%)	75,475 (20.0%)
140–179 mm Hg	81,570 (19.4%)	82,312 (21.8%)
≥180 mm Hg	10,972 (2.6%)	7,912 (2.1%)
Current treatment for hypertension	9,758 (2.3%)	6,501 (1.7%)
*Missing*	*0 (0*.*0%)*	*0 (0*.*0%)*
**Sociodemographic characteristics**		
Educational attainment		
<Primary school	236,555 (56.4%)	128,183 (34.1%)
Primary school	50,585 (12.1%)	51,021 (13.6%)
Middle school	50,218 (12.0%)	61,050 (16.3%)
Secondary school	40,320 (9.6%)	59,369 (15.8%)
High school	19,675 (4.7%)	32,860 (8.7%)
>High school	22,139 (5.3%)	43,169 (11.5%)
*Missing*	*1*,*199 (0*.*3%)*	*1*,*197 (0*.*3%)*
Urban area	136,426 (32.4%)	121,112 (32.2%)
*Missing*	*0 (0*.*0%)*	*0 (0*.*0%)*
Household wealth quintile		
*Missing*	*14 (0*.*0%)*	*10 (0*.*0%)*

Values are number (percent) unless otherwise indicated.

^1^This also includes respondents who had a normal blood glucose but reported being on treatment for diabetes.

BMI, body mass index; BP, blood pressure; SD, standard deviation.

### Cardiovascular risk at the national level

Overall, the mean 10-year risk of a CVD event in the (not age-standardized) population aged 30–74 years was 12.7% (95% CI: 12.7%–12.8%) among females and 21.4% (95% CI: 21.3%–21.6%) among males (Table B in S1 Table). The (not age-standardized) prevalence of a high CVD risk (10-year risk ≥ 30%) in those aged 30 to 74 years was 14.6% (95% CI: 14.4%–14.8%) among females and 31.7% (95% CI: 31.4%–32.0%) among males. The Framingham risk score yielded similar risk estimates to Harvard–NHANES, but substantially higher estimates than Globorisk and WHO–ISH (Table C in S1 Table). As an alternative measure of need for treatment and counseling to reduce CVD risk, we show the (not age-standardized) proportion of participants who were current smokers, had a high blood glucose, had hypertension, or were overweight in Table D in S1 Table.

### Geographic variation of cardiovascular risk

**Fig 1:**
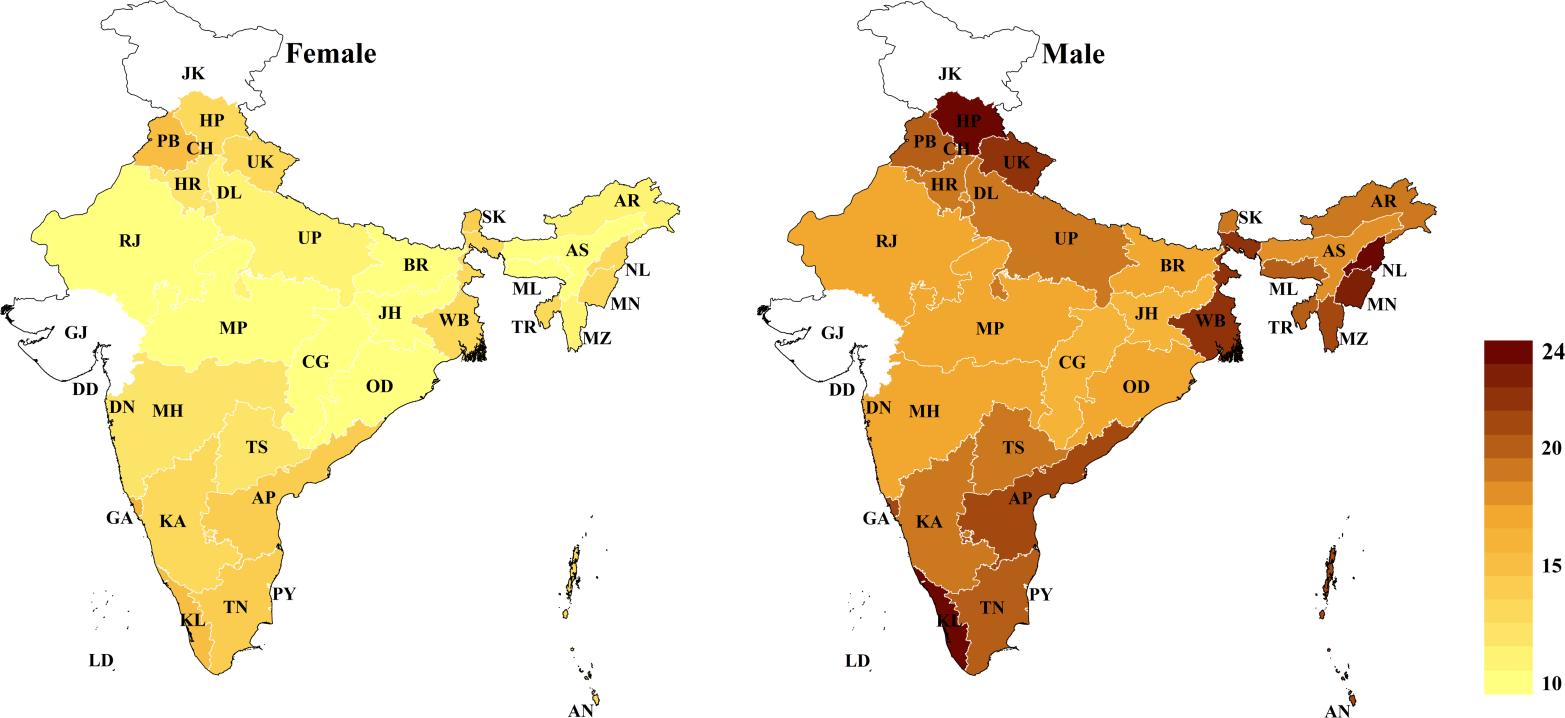
Age-standardized state-level mean 10-year risk of a cardiovascular disease event (percent as calculated by the Framingham risk score), by sex. The Global Burden of Disease project’s 2013 population for India was used for age standardization [36]. No data were available for Gujarat and Jammu and Kashmir. AN, Andaman and Nicobar Islands; AP, Andhra Pradesh; AR, Arunachal Pradesh; AS, Assam; BR, Bihar; CG, Chhattisgarh; CH, Chandigarh; DD, Daman and Diu; DL, Delhi; DN, Dadra and Nagar Haveli; GA, Goa; GJ, Gujarat; HR, Haryana; HP, Himachal Pradesh; JH, Jharkhand; JK, Jammu and Kashmir; KA, Karnataka; KL, Kerala; LD, Lakshadweep; MP, Madhya Pradesh; MH, Maharashtra; MN, Manipur; ML, Meghalaya; MZ, Mizoram; NL, Nagaland; OD, Odisha (Orissa); PB, Punjab; PY, Puducherry; RJ, Rajasthan; SK, Sikkim; TN, Tamil Nadu; TS, Telangana State; TR, Tripura; UP, Uttar Pradesh; UK, Uttarakhand (Uttaranchal); WB, West Bengal.

**Fig 2:**
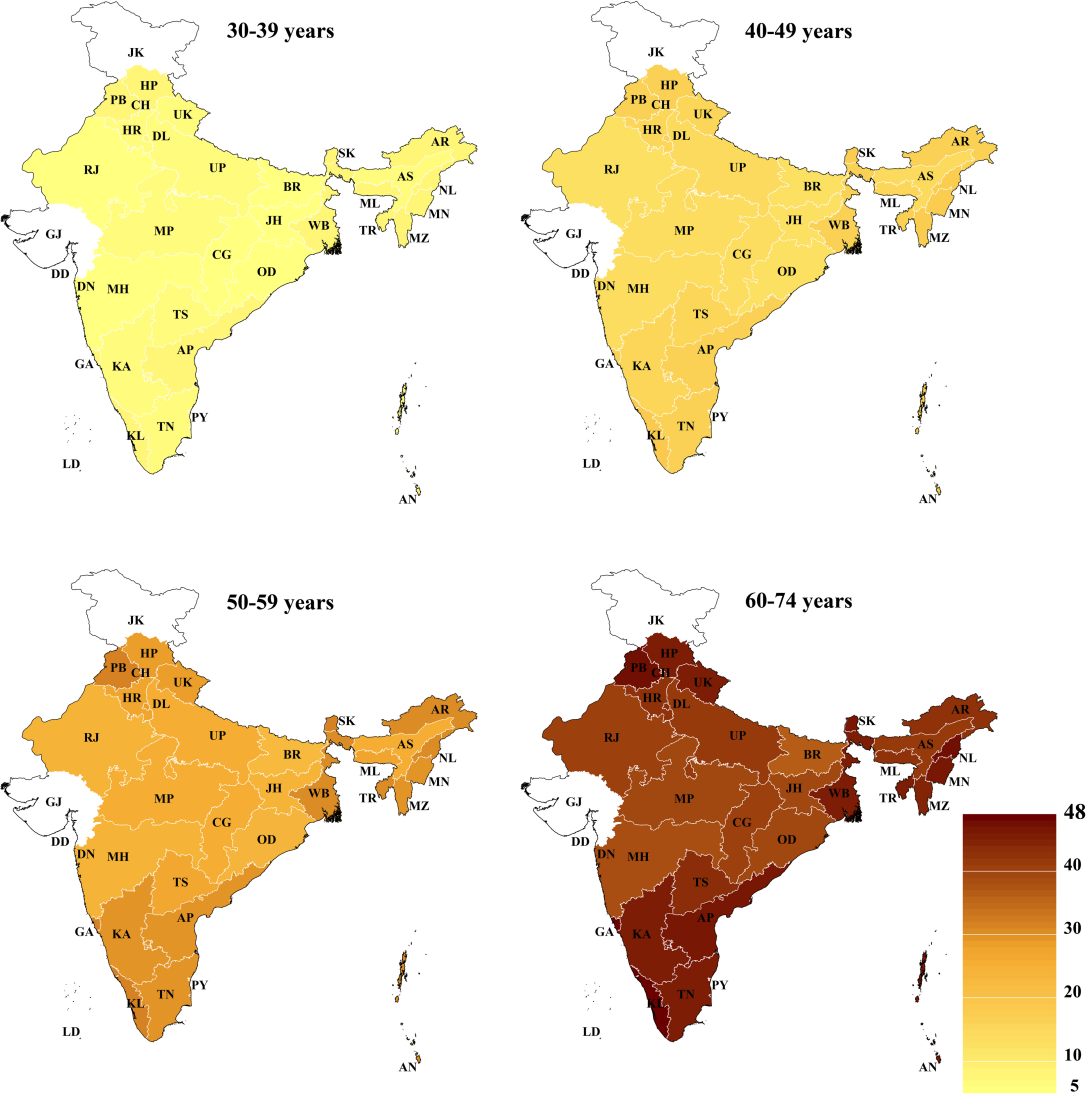
Age-standardized state-level mean 10-year risk of a cardiovascular disease event (percent as calculated by the Framingham risk score), by age group. The Global Burden of Disease project’s 2013 population for India was used for age standardization [36]. No data were available for Gujarat and Jammu and Kashmir. AN, Andaman and Nicobar Islands; AP, Andhra Pradesh; AR, Arunachal Pradesh; AS, Assam; BR, Bihar; CG, Chhattisgarh; CH, Chandigarh; DD, Daman and Diu; DL, Delhi; DN, Dadra and Nagar Haveli; GA, Goa; GJ, Gujarat; HR, Haryana; HP, Himachal Pradesh; JH, Jharkhand; JK, Jammu and Kashmir; KA, Karnataka; KL, Kerala; LD, Lakshadweep; MP, Madhya Pradesh; MH, Maharashtra; MN, Manipur; ML, Meghalaya; MZ, Mizoram; NL, Nagaland; OD, Odisha (Orissa); PB, Punjab; PY, Puducherry; RJ, Rajasthan; SK, Sikkim; TN, Tamil Nadu; TS, Telangana State; TR, Tripura; UP, Uttar Pradesh; UK, Uttarakhand (Uttaranchal); WB, West Bengal.

**Fig 3:**
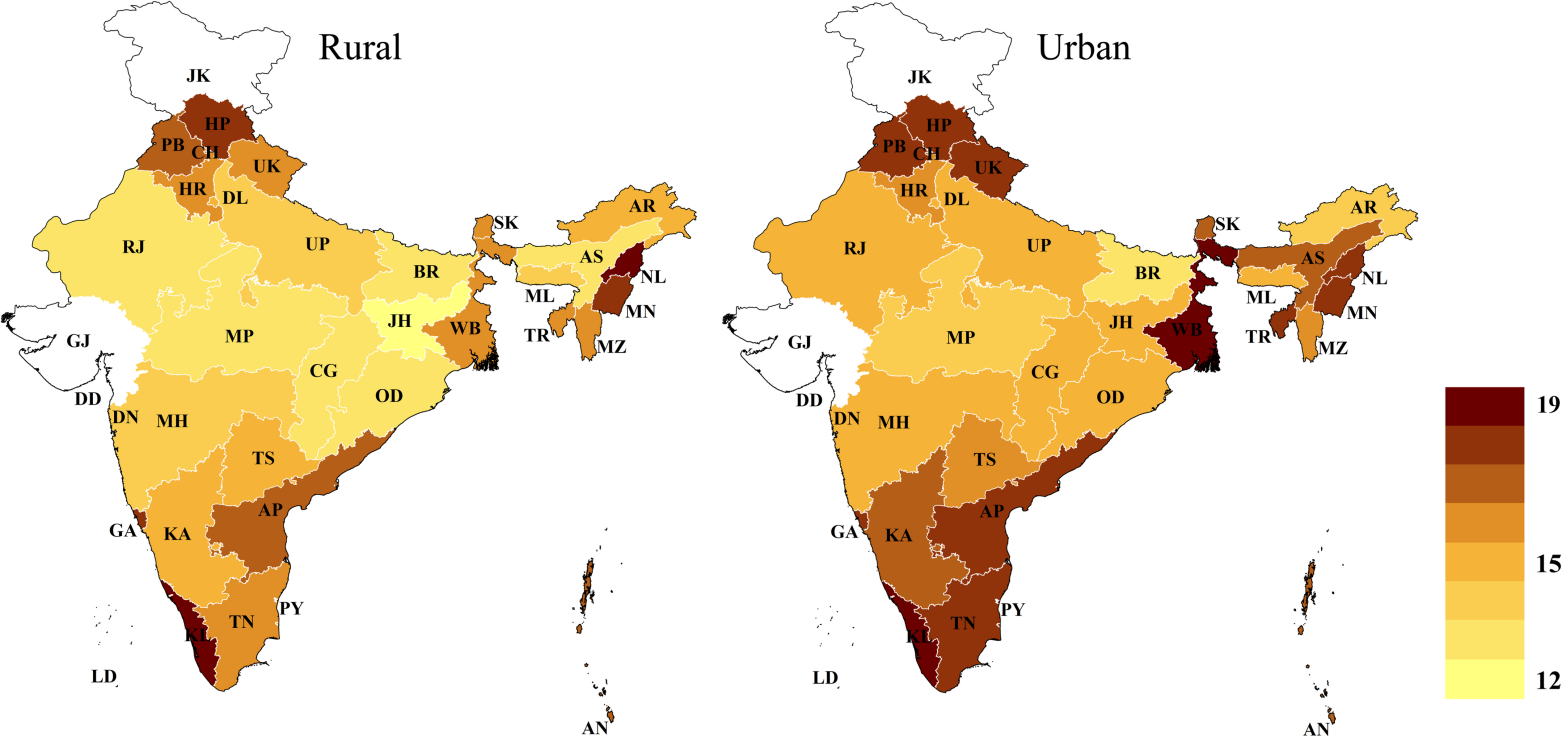
Age-standardized state-level mean 10-year risk of a cardiovascular disease event (percent as calculated by the Framingham risk score), by rural versus urban area. The Global Burden of Disease project’s 2013 population for India was used for age standardization [36]. No data were available for Gujarat and Jammu and Kashmir. AN, Andaman and Nicobar Islands; AP, Andhra Pradesh; AR, Arunachal Pradesh; AS, Assam; BR, Bihar; CG, Chhattisgarh; CH, Chandigarh; DD, Daman and Diu; DL, Delhi; DN, Dadra and Nagar Haveli; GA, Goa; GJ, Gujarat; HR, Haryana; HP, Himachal Pradesh; JH, Jharkhand; JK, Jammu and Kashmir; KA, Karnataka; KL, Kerala; LD, Lakshadweep; MP, Madhya Pradesh; MH, Maharashtra; MN, Manipur; ML, Meghalaya; MZ, Mizoram; NL, Nagaland; OD, Odisha (Orissa); PB, Punjab; PY, Puducherry; RJ, Rajasthan; SK, Sikkim; TN, Tamil Nadu; TS, Telangana State; TR, Tripura; UP, Uttar Pradesh; UK, Uttarakhand (Uttaranchal); WB, West Bengal.

The age-standardized state-level mean 10-year CVD risk (across all age groups) varied from 10.2% (95% CI: 9.8%–10.7%) among females in Assam to 24.2% among males in Nagaland (95% CI: 23.5%–25.0%) and Himachal Pradesh (95% CI: 23.6%–24.9%) (Fig 1). Similarly, the age-standardized prevalence of a high CVD risk varied from 5.0% (95% CI: 4.5%–5.6%) among females in Assam to 30.4% (95% CI: 28.8%–32.0%) among males in Kerala (Fig B in S1 Fig). Among both males and females, CVD risk tended to be highest in South India (including Goa), the 3 most northern states in the dataset (Himachal Pradesh, Punjab, and Uttarakhand), the northeastern states (except Assam), and West Bengal (particularly among males). This pattern across states, as well as the wide degree of variation in CVD risk between states, largely remained when examining state-level prevalence within only certain age groups (Fig 2) and within rural and urban areas (Fig 3). While the absolute risk levels depended strongly on the choice of CVD risk calculator, the relative variation across states was similar regardless of the CVD risk score used (Fig C in S1 Fig).

**Fig 4:**
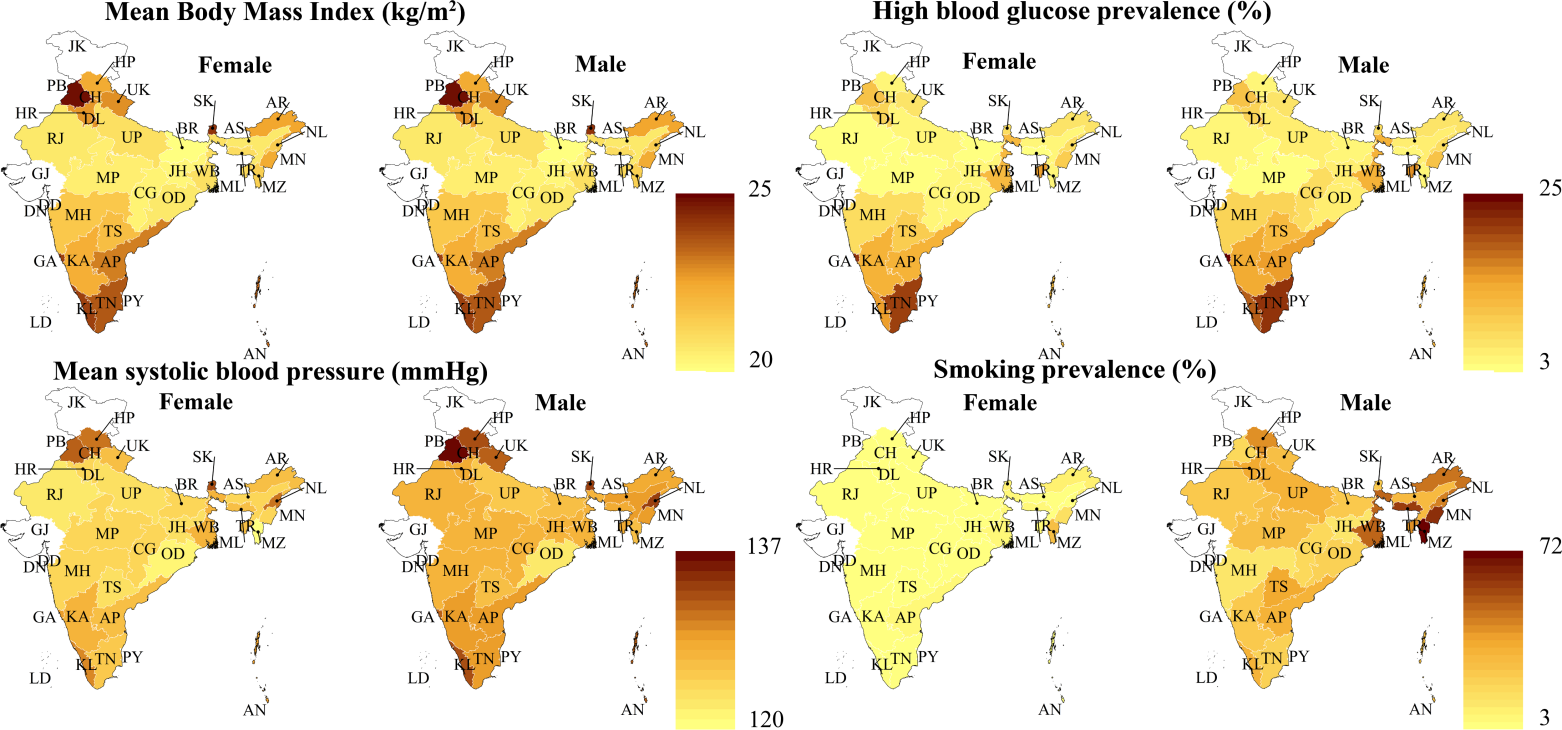
Body mass index, high blood glucose, systolic blood pressure, and smoking prevalence by state and sex. All outcome variables in this figure have been age-standardized using the Global Burden of Disease project’s 2013 population for India [36]. “Smoking” refers to smoking of any tobacco products but does not include chewing of tobacco. High blood glucose was defined as a high capillary blood glucose measurement (≥7.0 mmol/l if fasted and ≥11.1 mmol/l if non-fasted) or reporting to be on regular treatment for diabetes. AN, Andaman and Nicobar Islands; AP, Andhra Pradesh; AR, Arunachal Pradesh; AS, Assam; BR, Bihar; CG, Chhattisgarh; CH, Chandigarh; DD, Daman and Diu; DL, Delhi; DN, Dadra and Nagar Haveli; GA, Goa; GJ, Gujarat; HR, Haryana; HP, Himachal Pradesh; JH, Jharkhand; JK, Jammu and Kashmir; KA, Karnataka; KL, Kerala; LD, Lakshadweep; MP, Madhya Pradesh; MH, Maharashtra; MN, Manipur; ML, Meghalaya; MZ, Mizoram; NL, Nagaland; OD, Odisha (Orissa); PB, Punjab; PY, Puducherry; RJ, Rajasthan; SK, Sikkim; TN, Tamil Nadu; TS, Telangana State; TR, Tripura; UP, Uttar Pradesh; UK, Uttarakhand (Uttaranchal); WB, West Bengal.

Fig 4 shows differences between states in the age-standardized mean (for BMI and systolic BP) or prevalence (for high blood glucose and smoking) for each of the CVD risk factors that are included in the CVD risk score. Mean BMI was high in both northern (Haryana, Himachal Pradesh, Punjab, and Uttarakhand) and southern states (Andhra Pradesh, Goa, Karnataka, Kerala, Tamil Nadu), ranging from 22.8 kg/m^2^ among males in Uttarakhand to 25.1 kg/m^2^ among females in Punjab. High blood glucose prevalence, however, was relatively low in the northern states (ranging from 4.4% among males in Himachal Pradesh to 10.9% among females in Punjab). Mean systolic BP was highest in the northern states (ranging from 123.7 mm Hg among females in Haryana to 136.2 mm Hg among males in Punjab) as well as in Nagaland and Sikkim (130.7 mm Hg and 132.8 mm Hg among females and 133.6 mm Hg and 133.1 mm Hg among males, respectively). Smoking was most prevalent among males in the northeastern states of Arunachal Pradesh (46.4%), Manipur (60.3%), Meghalaya (59.7%), and Mizoram (71.7%) and the eastern state of West Bengal (49.5%). As with the CVD risk score, these patterns across states and the wide variation between states remained when examining the state-level distribution of these variables only within certain age groups and within rural and urban areas (Fig D in S1 Fig).

### Socioeconomic drivers of geographic variation in cardiovascular risk

**Fig 5:**
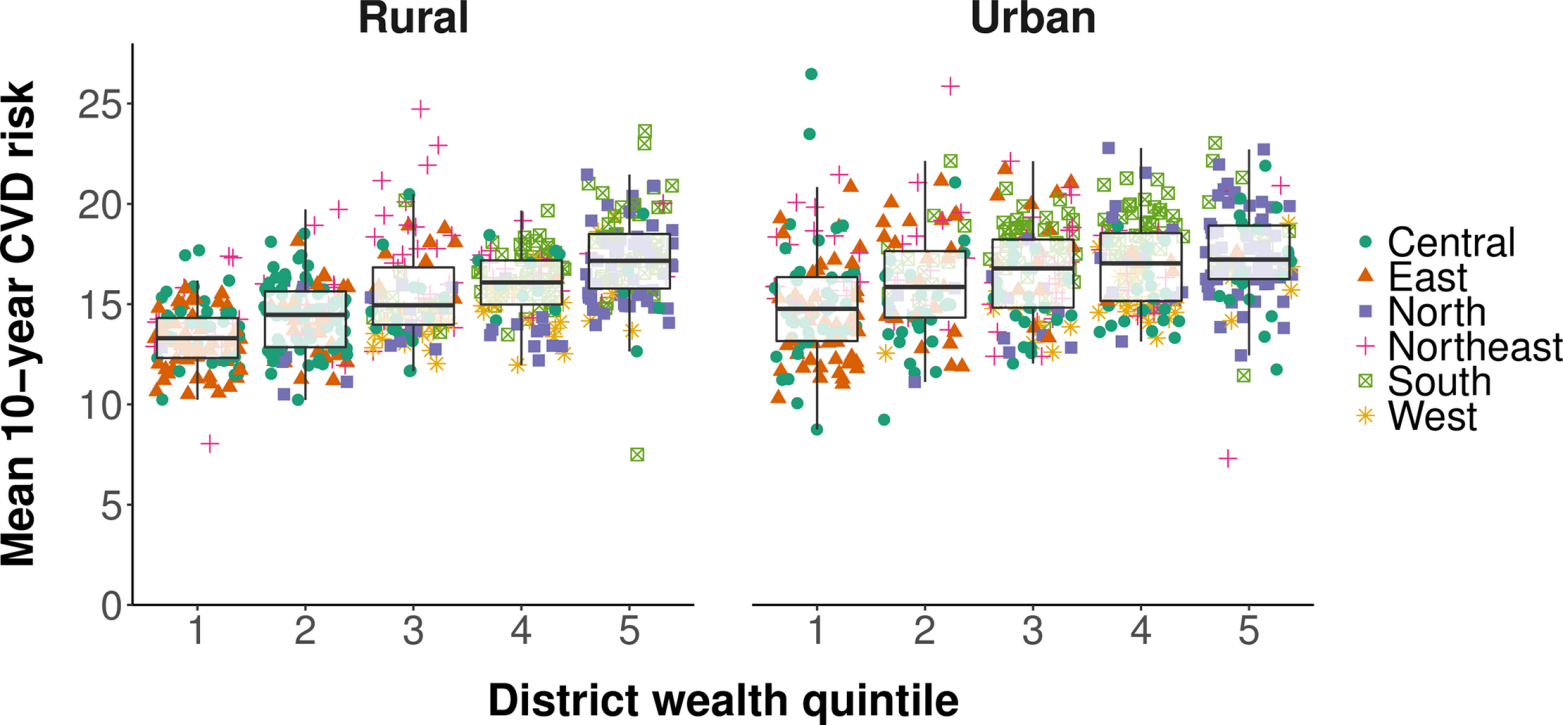
Association between the age-standardized district-level mean 10-year cardiovascular disease risk and district wealth quintile. Mean 10-year risk of a cardiovascular disease (CVD) event was calculated using the Framingham risk score. District wealth quintile was calculated, separately for rural and urban areas within districts, by computing the median of the continuous household wealth index in a district and then categorizing the district-level median into quintiles. Age standardization was to the Global Burden of Disease project’s 2013 population structure for India [36]. The sample in each district was restricted to those aged 30 to 74 years. States and districts were divided into regions as per their allocation to Zonal Councils by the Government of India [39]. The whiskers of the box and whisker diagrams end at 1.5 × interquartile range.

**Fig 6:**
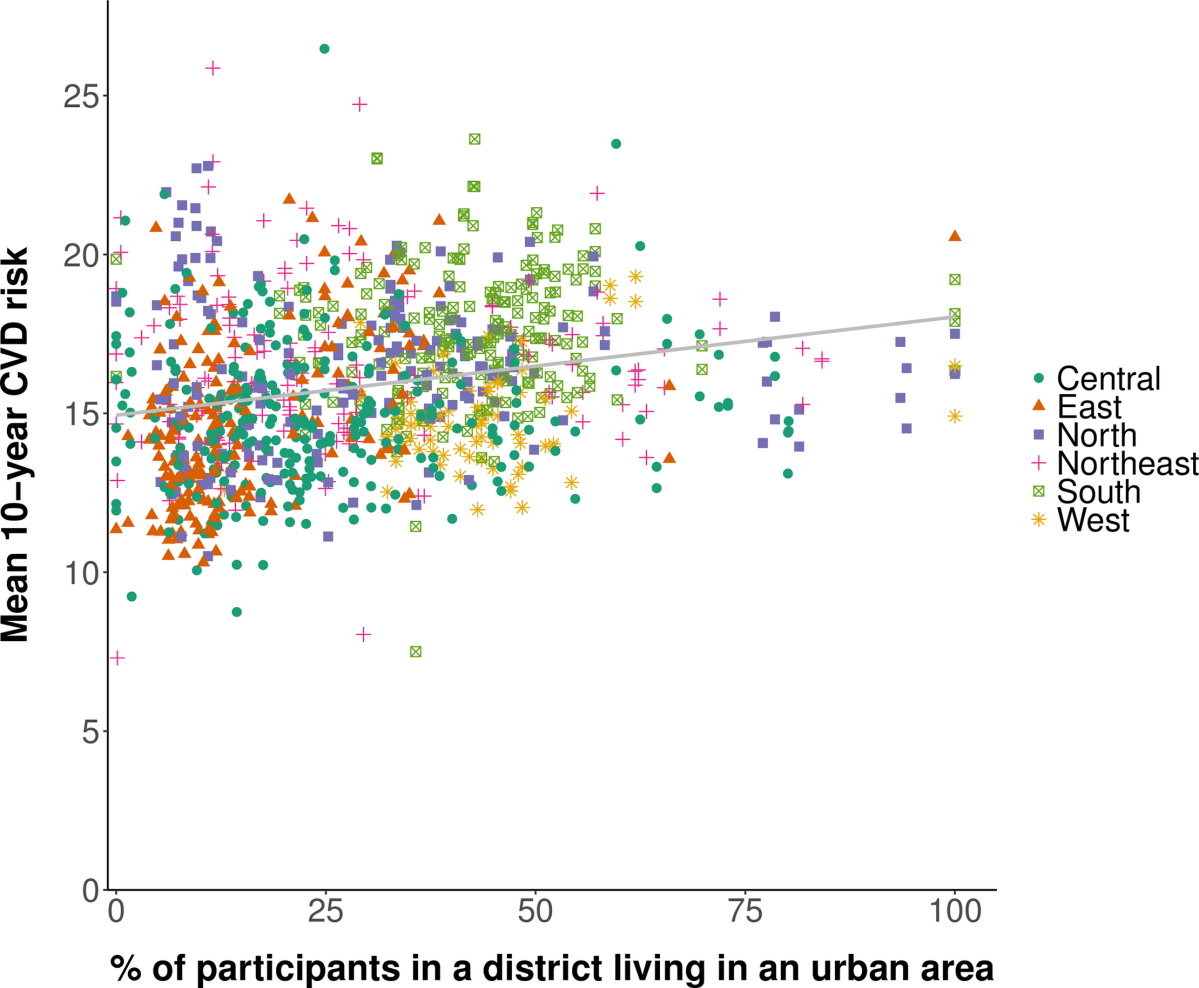
Association between the age-standardized district-level mean 10-year cardiovascular disease risk and urbanization. Mean 10-year risk of a cardiovascular disease (CVD) event was calculated using the Framingham risk score. Urbanization refers to the district-level percentage of adults aged 30 to 74 years in our sample who were living in an urban area. Age standardization was to the Global Burden of Disease project’s 2013 population structure for India [36]. The sample in each district was restricted to those aged 30 to 74 years. States and districts were divided into regions as per their allocation to Zonal Councils by the Government of India [39]. The grey line was fitted using ordinary least squares regression (with each data point in the plot having the same weight).

We found a positive association between the mean CVD risk in a district and the district’s wealth when plotting the district-level mean Framingham risk score against the district-level median (categorized into quintiles) of the continuous household wealth index (Fig 5). Similarly, mean CVD risk was positively associated with the proportion of the sample in a district that was living in an urban area (Fig 6).

Confirming the impression from the plotting of our data in Figs 5 and 6, our multivariable linear regressions revealed that district wealth quintile was positively associated with CVD risk in both rural and urban areas, with the association stronger in rural areas (Table 3). Specifically, among participants residing in rural areas, living in the wealthiest 20% of districts in India was associated with a relative increase in the 10-year CVD risk of 13.1% (95% CI: 10.7%–15.6%; *p <* 0.001) compared to the poorest 20% of districts. In urban areas, the corresponding increase was only 4.3% (95% CI: 1.5%–7.1%; *p =* 0.003). In addition, as shown in Table 4, living in an entirely urbanized district was associated with a relative increase in the 10-year CVD risk of 16.9% (95% CI: 12.7%–21.1%; *p <* 0.001) compared with living in an entirely rural district. The associations shown in Tables 3 and 4 were similar regardless of the CVD risk calculator used (Tables G–J in S1 Table).

**Table 3:** Multivariable linear regression of the natural logarithm of 10-year cardiovascular disease risk on district wealth quintile and individual-level sociodemographic characteristics.

Characteristic	Rural areas		Urban areas	
	Coefficient^1^ (95% CI)	*p*-Value	Coefficient^1^ (95% CI)	*p*-Value
District wealth quintile				
1 (poorest)	Ref.		Ref.	
2	1.93 (0.38–3.47)	0.014	3.46 (1.37 to 5.55)	0.001
3	7.53 (5.27–9.79)	<0.001	2.10 (−0.24 to 4.45)	0.079
4	4.74 (2.48–7.01)	<0.001	2.95 (0.59 to 5.30)	0.014
5 (richest)	13.12 (10.66–15.59)	<0.001	4.26 (1.45 to 7.07)	0.003
Household wealth quintile				
1 (poorest)	Ref.		Ref.	
2	0.55 (0.17–0.93)	0.005	4.09 (3.49 to 4.68)	<0.001
3	2.18 (1.76–2.59)	<0.001	8.15 (7.54 to 8.77)	<0.001
4	4.99 (4.54–5.43)	<0.001	10.57 (9.93 to 11.21)	<0.001
5 (richest)	11.29 (10.78–11.79)	<0.001	12.09 (11.39 to 12.79)	<0.001
Educational attainment				
<Primary school	Ref.		Ref.	
Primary school	2.00 (1.62–2.38)	<0.001	2.44 (1.81 to 3.07)	<0.001
Middle school	1.95 (1.56–2.33)	<0.001	2.43 (1.83 to 3.03)	<0.001
Secondary school	2.61 (2.16–3.06)	<0.001	2.72 (2.12 to 3.31)	<0.001
High school	1.06 (0.45–1.66)	<0.001	0.87 (0.16 to 1.58)	0.016
>High school	1.19 (0.52–1.86)	<0.001	0.15 (−0.51 to 0.81)	0.659

The model included random intercepts by district. The model included all variables listed in the table, 5-year age groups, and a binary indicator for sex as explanatory variables. Ten-year cardiovascular disease risk was calculated using the Framingham risk score. The dataset was first divided into participants living in rural versus urban areas before district wealth quintile was calculated and the regression model was fitted. District wealth quintile was calculated, separately for rural and urban areas within districts, by computing the median of the continuous household wealth index in a district and then categorizing the district-level median into quintiles.

^1^Coefficients were multiplied by 100 so that they can be interpreted as an approximation of the percentage change in cardiovascular disease risk associated with a 1-unit change in the explanatory variable.

CI, confidence interval; Ref., reference category.

**Table 4:** Multivariable linear regression of the natural logarithm of 10-year cardiovascular disease risk on the proportion of participants in a district who live in an urban area.

Characteristic	Coefficient^1^ (95% CI)	*p*-Value
District-level proportion living in an urban area^2^	16.91 (12.69–21.13)	<0.001
Household wealth quintile		
1 (poorest)	Ref.	
2	0.73 (0.41–1.05)	<0.001
3	2.08 (1.74–2.42)	<0.001
4	3.82 (3.46–4.17)	<0.001
5 (richest)	7.54 (7.15–7.94)	<0.001
Educational attainment		
<Primary school	Ref.	
Primary school	4.01 (3.69–4.34)	<0.001
Middle school	4.76 (4.44–5.08)	<0.001
Secondary school	6.57 (6.23–6.91)	<0.001
High school	5.53 (5.08–5.97)	<0.001
>High school	6.24 (5.82–6.66)	<0.001

The model included random intercepts by district. The model included all variables listed in the table, 5-year age groups, and a binary indicator for sex as explanatory variables. Ten-year cardiovascular disease risk was calculated using the Framingham risk score.

^1^Coefficients were multiplied by 100 so that they can be interpreted as an approximation of the percentage change in cardiovascular disease risk associated with a 1-unit change in the explanatory variable.

^2^“District-level proportion living in an urban area” refers to the proportion (between 0 and 1) of the participants in a district who live in an urban area.

CI, confidence interval; Ref., reference category.

### Cardiovascular risk by individual-level sociodemographic characteristics

**Fig 7:**
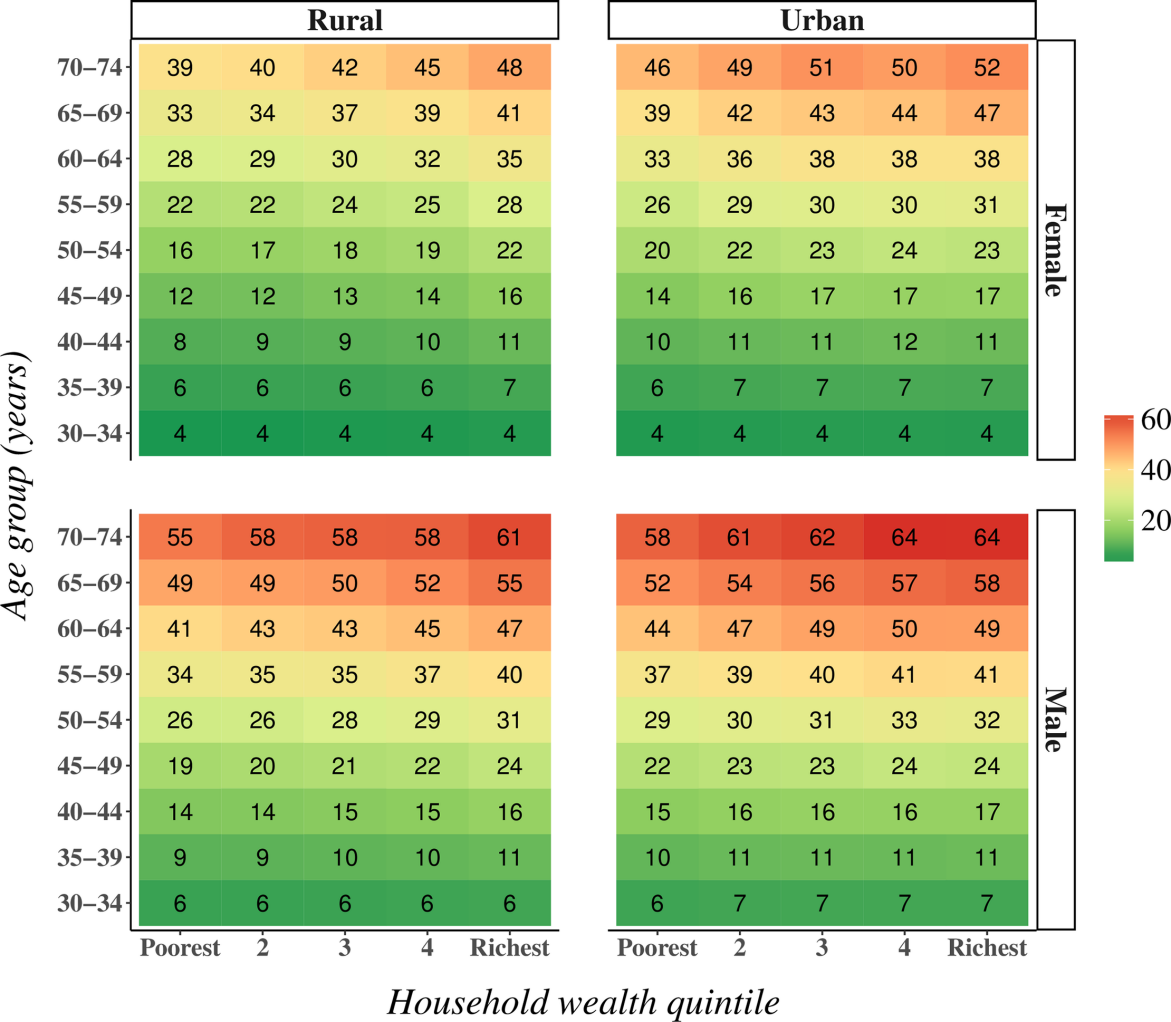
Crude mean 10-year cardiovascular disease risk by household wealth quintile, age group, rural versus urban location, and sex. This is the crude (i.e., not age-standardized) mean 10-year risk (in percent) of a cardiovascular disease event as calculated with the Framingham risk score.

Stratifying mean 10-year CVD risk by age group, sex, rural versus urban location, and household wealth quintile shows that (i) those living in urban areas generally had a higher CVD risk than those living in rural areas, (ii) irrespective of sex and location, mean CVD risk was higher in the wealthiest than in the poorest quintile in all age groups (except the youngest age group), and (iii) both the relative and absolute differences in mean CVD risk between wealth quintiles tended to be larger in rural than in urban areas (Fig 7). These patterns were generally similar when using Harvard–NHANES or Globorisk (WHO–ISH does not yield a continuous risk score) instead of the Framingham risk score (Fig E in S1 Fig), and when examining the prevalence of a high 10-year CVD risk (≥30%) as opposed to mean CVD risk (Fig F in S1 Fig).

Table 5 shows the regression coefficients (which can be interpreted as approximations of the percentage change in CVD risk) when regressing the natural logarithm of the Framingham risk score on individuals’ sociodemographic characteristics and a fixed effect for district. Household wealth quintile, education, and living in an urban area were positively associated with CVD risk among both sexes, but for all 3 variables the coefficients for males were substantially smaller than those for females. The association between education and CVD risk was weak once the regressions were adjusted for other sociodemographic characteristics. The regression results were similar when using Harvard–NHANES or Globorisk (WHO–ISH does not yield a continuous risk score) (Tables K and L in S1 Table).

**Table 5:** Ordinary least squares regressions of the natural logarithm of cardiovascular risk on sociodemographic covariates and a district-level fixed effect.

	Female (*n =* 419,478)				Male (*n =* 375,642)			
	Adjusted for age group only^1^		Adjusted for all covariates^2^		Adjusted for age group only^1^		Adjusted for all covariates^2^	
	Coefficient^3^ (95% CI)	*p*-Value	Coefficient^3^ (95% CI)	*p*-Value	Coefficient^3^ (95% CI)	*p*-Value	Coefficient^3^ (95% CI)	*p*-Value
Household wealth quintile								
1 (poorest)	Ref.		Ref.		Ref.		Ref.	
2	2.22 (1.75–2.69)	<0.001	2.44 (1.97 to 2.91)	<0.001	0.64 (0.22–1.05)	0.003	0.83 (0.42–1.25)	<0.001
3	4.68 (4.18–5.17)	<0.001	5.32 (4.82 to 5.82)	<0.001	1.97 (1.53–2.41)	<0.001	2.45 (2.00–2.90)	<0.001
4	7.47 (6.96–7.98)	<0.001	8.31 (7.79 to 8.84)	<0.001	3.91 (3.46–4.36)	<0.001	4.55 (4.08–5.02)	<0.001
5 (richest)	13.11 (12.57–13.66)	<0.001	14.14 (13.55 to 14.74)	<0.001	7.71 (7.23–8.19)	<0.001	8.44 (7.91–8.97)	<0.001
Educational attainment								
<Primary school	Ref.		Ref.		Ref.		Ref.	
Primary school	7.20 (6.72–7.67)	<0.001	4.03 (3.54 to 4.51)	<0.001	1.99 (1.57–2.41)	<0.001	0.86 (0.44–1.28)	<0.001
Middle school	8.80 (8.31–9.29)	<0.001	4.09 (3.59 to 4.59)	<0.001	2.69 (2.29–3.10)	<0.001	0.83 (0.42–1.24)	<0.001
Secondary school	11.61 (11.07–12.15)	<0.001	4.59 (4.02 to 5.16)	<0.001	5.35 (4.95–5.76)	<0.001	2.23 (1.80–2.66)	<0.001
High school	10.71 (9.98–11.44)	<0.001	2.12 (1.36 to 2.88)	<0.001	4.59 (4.09–5.10)	<0.001	0.54 (0.01–1.07)	0.047
>High school	10.69 (9.99–11.39)	<0.001	−0.66 (−1.42 to 0.10)	0.087	7.06 (6.60–7.52)	<0.001	1.09 (0.57–1.61	<0.001
Location								
Rural	Ref.		Ref.		Ref.		Ref.	
Urban	11.96 (11.62–12.30)	<0.001	12.51 (12.14 to 12.88)	<0.001	6.92 (6.62–7.23)	<0.001	7.35 (7.02–7.68)	<0.001

Standard errors were adjusted for clustering at the level of the primary sampling unit.

^1^These models included 1 sociodemographic characteristic, age group, and a binary indicator variable for each district as explanatory variables.

^2^These models included all variables listed in the table, age group, and a binary indicator for each district as explanatory variables.

^3^Coefficients were multiplied by 100 so that they can be interpreted as an approximation of the percentage change in cardiovascular risk associated with a 1-unit change in the explanatory variable.

CI, confidence interval; Ref., reference category.

**Fig 8:**
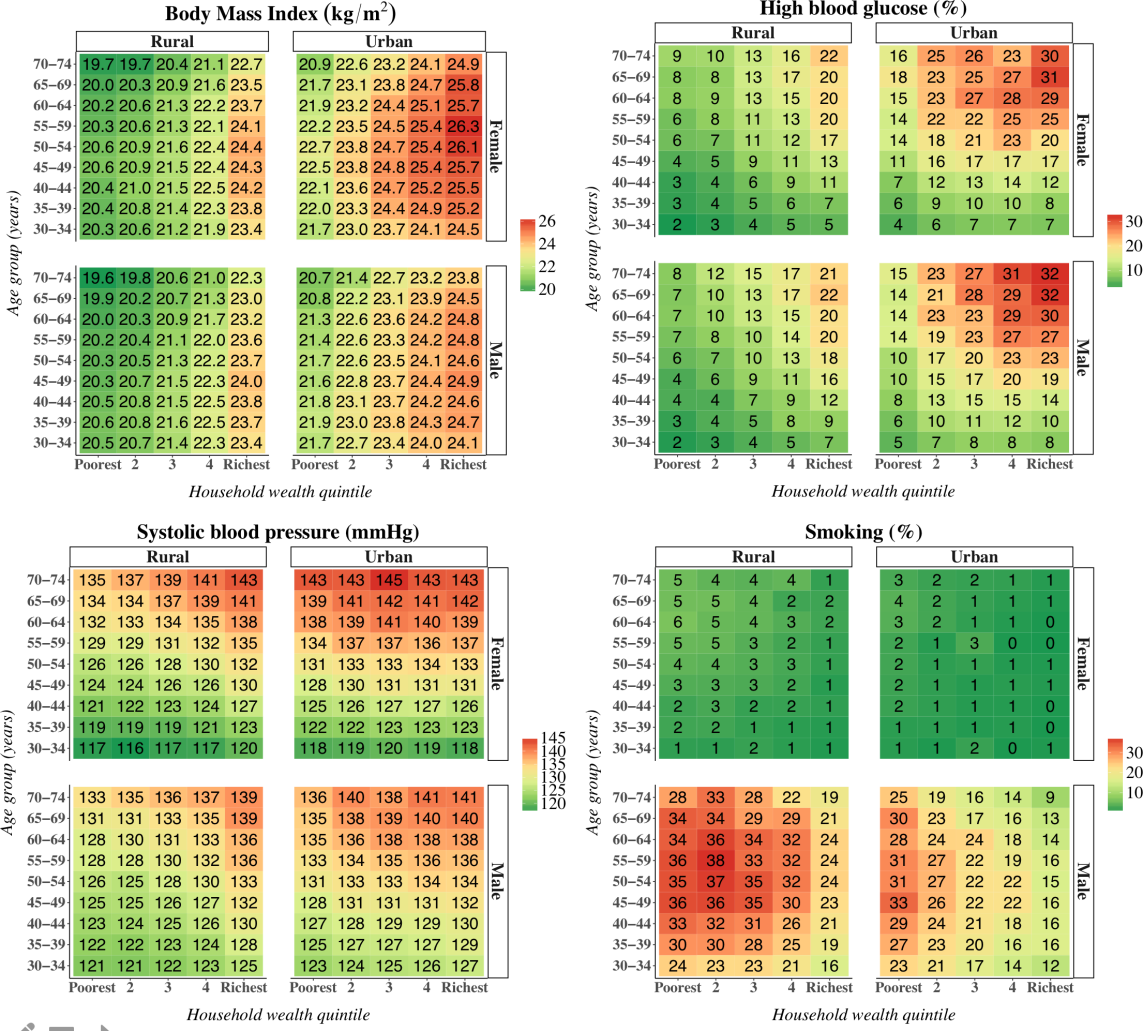
Mean body mass index, high blood glucose prevalence, smoking prevalence, and mean systolic blood pressure by rural versus urban residence, sex, and household wealth quintile. These are crude (not age-standardized) estimates. “Smoking” refers to smoking of any tobacco products but does not include chewing of tobacco. High blood glucose was defined as a high capillary blood glucose measurement (≥7.0 mmol/l if fasted and ≥11.1 mmol/l if non-fasted) or reporting to be on regular treatment for diabetes.

Fig 8 shows that while mean BMI, high blood glucose, and mean systolic BP were all positively associated with household wealth and living in an urban area, the prevalence of high blood glucose and mean systolic BP were nonetheless high in middle and old age among the poorest wealth quintiles and in rural areas. Smoking, on the other hand, was more common in poorer quintiles, in rural areas, and among males.

## Discussion

Pooling and analyzing data on CVD risk for 797,540 adults across India (a country that accounts for more than one-sixth of the world’s population [4]), we identified important variation in risk among states (with CVD risk tending to be highest in the northern, northeastern, and southern states) and by individuals’ sociodemographic characteristics. In particular, we found that (i) CVD risk was higher in urban areas and among males, (ii) while mean BMI was substantially higher among wealthy than poor individuals, high blood glucose and high systolic BP were common among poor individuals in middle and old age, and (iii) smoking was most prevalent among men, in poorer wealth quintiles, and in rural areas. Thus, while a major investment in CVD and risk factor prevention, screening, and treatment is needed across India, this study provides important new insights on the distribution of CVD risk to effectively target health system resources for CVD management to those most at risk and most in need. Given that we found that district-level mean CVD risk was positively associated with district wealth and urbanization, such investments may be crucial to minimize further rises in CVD risk as socioeconomic development and urbanization in India progress over the coming decades.

Even though the Globorisk and WHO–ISH scores were developed specifically with the goal of providing CVD risk estimates in populations for which no validated CVD risk calculator exists [22,32,33], the absence of a CVD risk equation that has been validated in South Asian cohorts is a major limitation of this study. Nonetheless, CVD risk calculators are used routinely in clinical settings (where they are employed in conjunction with a clinical assessment) in India [40]. Although this does not necessarily justify their employment at the population level, there has been a recent move to applying these risk equations to entire populations. For instance, one of the WHO’s NCD Global Action Plan targets (that “at least 50% of eligible people receive drug therapy and counselling to prevent heart attacks and strokes” by 2020, for which the WHO defined eligibility as a 10-year CVD risk ≥ 30% [30]) is based on the concept of applying CVD risk equations to the population level. In addition, several recent studies have used CVD risk calculators for population-level assessments of CVD risk [22,32,41]. Nevertheless, we wish to emphasize here that the absolute risk predictions provided in this study should be interpreted with caution. Indeed, the lack of validation in South Asian populations may be one reason that our risk estimates varied widely across CVD calculators. Specifically, the Framingham and Harvard–NHANES risk scores yielded substantially higher estimates than Globorisk and WHO–ISH. This observed difference in estimates was expected to some degree given that Globorisk and WHO–ISH predict the risk of (fatal or nonfatal) myocardial infarction or stroke, whereas the Framingham and Harvard–NHANES risk scores include a broader set of outcomes (Table 1). Having acknowledged this limitation, we do believe that the CVD risk predictions are useful as a summary measure of CVD risk when assessing variation of risk among population groups. In this regard, it is important to highlight that the patterns of variation in CVD risk by state, rural versus urban residence, and individual-level sociodemographic characteristics were very similar across the 4 different risk calculators used in this study.

This study has several additional limitations. First, a relatively high percentage (27.1%) of participants had a missing value for at least 1 variable needed to calculate their CVD risk. While we show that participants excluded because of a missing value had similar summary statistics for CVD risk factors as those included in the analysis, there is nonetheless potential for selection bias. Second, a 1-time capillary blood glucose measurement is not recommended for the diagnosis of diabetes in clinical settings [42]. However, this screening method has been shown to have an acceptable sensitivity and specificity for defining diabetes in population-based research, and is hence the recommended method for monitoring diabetes prevalence in the WHO’s STEPwise Approach to Noncommunicable Disease Risk Factor Surveillance [43–45]. Nonetheless, to be clear about this limitation of our data, we refer to high blood glucose values (or being on treatment for diabetes) as “high blood glucose” in this paper rather than “diabetes.” Third, the questionnaire used in both the DLHS-4 and AHS was designed such that only those who answered in the affirmative to having had symptoms (of any type) lasting for more than 1 month during the last 1 year were asked whether they were “getting regular treatment” for the condition. Our data thus likely underestimate the number of participants who were on treatment for hypertension and diabetes. Fourth, we were unable to exclude participants with a current or previous CVD (e.g., a previous myocardial infarction) because data on participants’ medical history were not collected. Since those with a previous or current CVD tend to have a higher CVD risk than predicted with a CVD risk score, this limitation biases our CVD risk estimates for the population of India downwards. Lastly, the CVD risk scores used here do not take into account consumption of smokeless tobacco, which is common in India and may increase CVD risk [46,47].

In conclusion, this study identified important variation in CVD risk and risk factor prevalence among states and population groups in India—information that will be essential for effective targeting of resources and interventions for prevention, screening, and treatment to those most at risk and most in need. Such investments in targeted CVD care programs as well as relevant health policy measures are urgently needed—particularly in states with a high CVD risk—if India is to minimize CVD’s adverse consequences for health, well-being, financial risk protection, and economic growth. Given the size and projected growth of India’s population, the determination and effectiveness of the country’s measures to prevent and treat CVD over the coming years will have an important bearing on the achievement of the SDGs at the global level.

## Supporting information

S1 Checklist STROBE checklist. (PDF)

S1 Fig All supplementary figures. (DOCX)

S1 Table All supplementary tables. (DOCX)

S1 Text Matching AHS biomarker data to participants’ sociodemographic data. (DOCX)

## Abbreviations

AHS: Annual Health Survey
BMI: body mass index
BP: blood pressure
CAB: clinical, anthropometric, and biomarker
CVD: cardiovascular disease
DLHS-4: District Level Household Survey–4
PSU: primary sampling unit
SDG: Sustainable Development Goal
SSU: secondary sampling unit
