# Supplementary material for: Geographic and sociodemographic variation of cardiovascular disease risk in India: A cross-sectional study of 797,540 adults
Source: PLoS Med. 2018 Jun 19;15(6):e1002581. doi: 10.1371/journal.pmed.1002581 (PMC6007838; doi:10.1371/journal.pmed.1002581)
Supplement: S1 Fig — (DOCX) [file pmed.1002581.s002.docx]

**Supplementary Figures**

Figure A. States and Union Territories covered by each survey 3

Figure B. Age-standardized state-level prevalence of a high CVD risk (as calculated by the Framingham risk score) 4

Figure C. Age-standardized cardiovascular disease risk by state as calculated with each risk score 7

Figure D. Cardiovascular disease risk factors by state 22

Figure E. Crude mean 10-year Harvard-NHANES and Globorisk score by household wealth quintile, age group, rural versus urban location, and sex 33

Figure F. Percentage of population with a high (≥30%) 10-year risk score by household wealth quintile, age group, rural versus urban location, and sex 35

Figure G. Mean diastolic blood pressure by household wealth quintile, age group, rural versus urban location, and sex.^1^ 39

References: 40

**Combined legend for all figures in this file:**

^1^ High CVD risk was defined as a 10-year cardiovascular disease risk ≥30% as calculated with the Framingham risk score.

^2^ The Global Burden of Disease Project’s 2013 population for India was used for age standardization.[1]

^3^ No data was available for Gujarat, and Jammu and Kashmir.

^4^ ‘High blood glucose’ was defined as a high capillary blood glucose measurement (≥126mg/dl if fasted and ≥200mg/dl if non-fasted) or reporting to be on regular treatment for diabetes.

^5^ ‘Smoking’ refers to smoking of any tobacco products but does not include chewing of tobacco.

^6^ This is the crude (i.e., age-unstandardized) mean 10-year risk (in percent) of a CVD event as calculated by the Harvard-NHANES score.

^7^ This is the crude (i.e., age-unstandardized) mean 10-year risk (in percent) of a CVD event as calculated by the Globorisk score.

^8^ Globorisk estimates cardiovascular risk only for those aged 40 to 74 years.

^9^ This is the crude prevalence (disaggregated by household wealth quintile, age group, rural-urban residence, and sex) of a high (≥30%) 10-year CVD risk as computed with the Framingham risk score.

^10^ This is the crude prevalence (disaggregated by household wealth quintile, age group, rural-urban residence, and sex) of a high (≥30%) 10-year CVD risk as computed with the Harvard-NHANES score.

^11^ This is the crude prevalence (disaggregated by household wealth quintile, age group, rural-urban residence, and sex) of a high (≥30%) 10-year CVD risk as computed with the Globorisk score.

^12^ This is the crude prevalence (disaggregated by household wealth quintile, age group, rural-urban residence, and sex) of a high (≥30%) 10-year CVD risk as computed with the WHO-ISH score.

**Figure A. States and Union Territories covered by each survey**

**
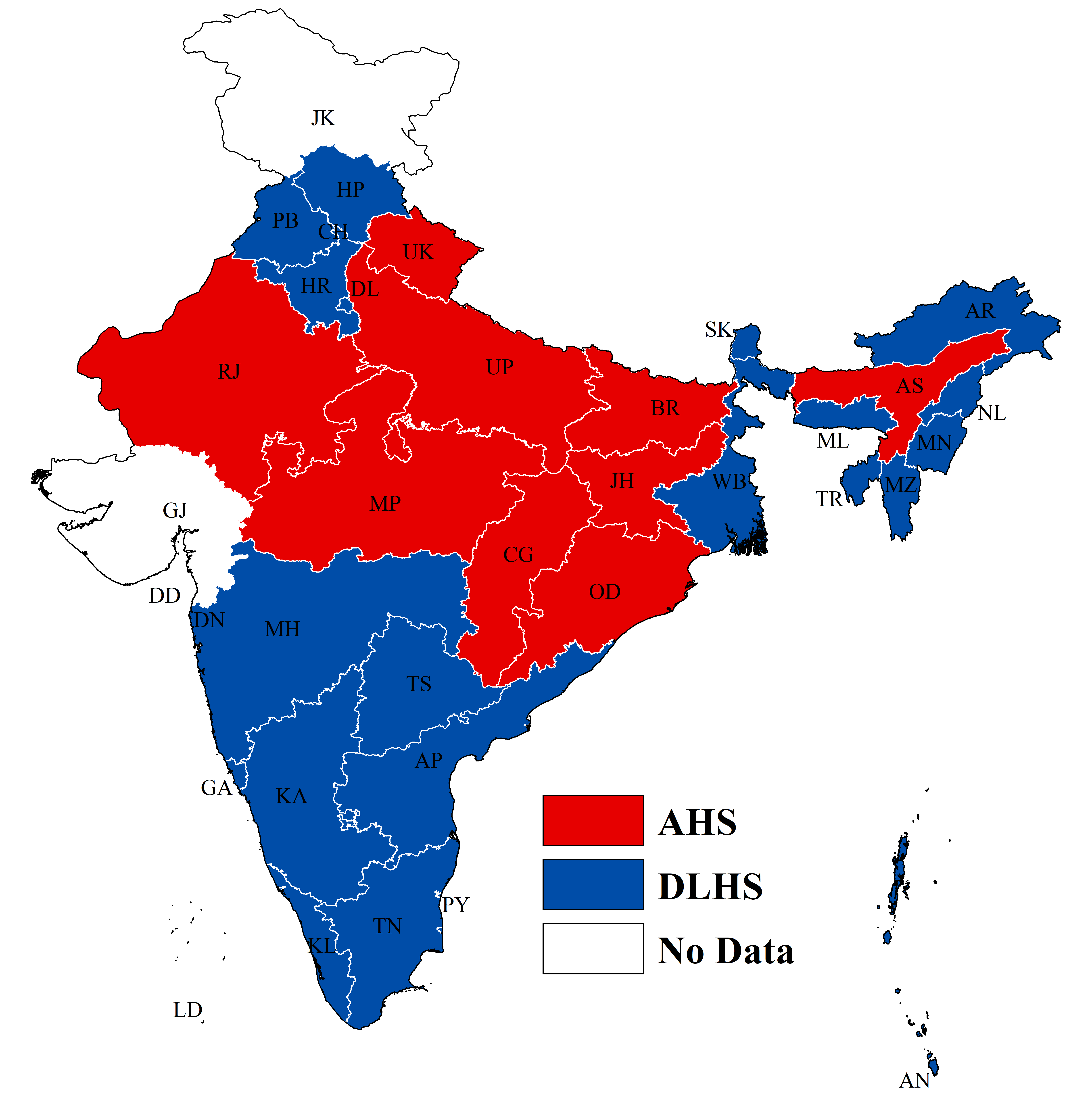
**

**Abbreviations:** AP indicates Andhra Pradesh; AR, Arunachal Pradesh; AS, Assam; BR, Bihar; CG, Chhattisgarh; CH, Chandigarh; DD, Daman and Diu; DL, Delhi; GA, Goa; GJ, Gujarat; HR, Haryana; HP, Himachal Pradesh; JH, Jharkhand; JK, Jammu and Kashmir; KA, Karnataka; KL, Kerala; MP, Madhya Pradesh; MH, Maharashtra; MN, Manipur; ML, Meghalaya; MZ, Mizoram; NL, Nagaland; OD, Odisha (Orissa); PB, Punjab; PY, Puducherry; RJ, Rajasthan; SK, Sikkim; TN, Tamil Nadu; TS, Telangana State; TR, Tripura; UP, Uttar Pradesh; UK, Uttarakhand (Uttaranchal); WB, West Bengal.

**Figure B. Age-standardized state-level prevalence of a high CVD risk (as calculated by the Framingham risk score)** ^1,2,3^

^
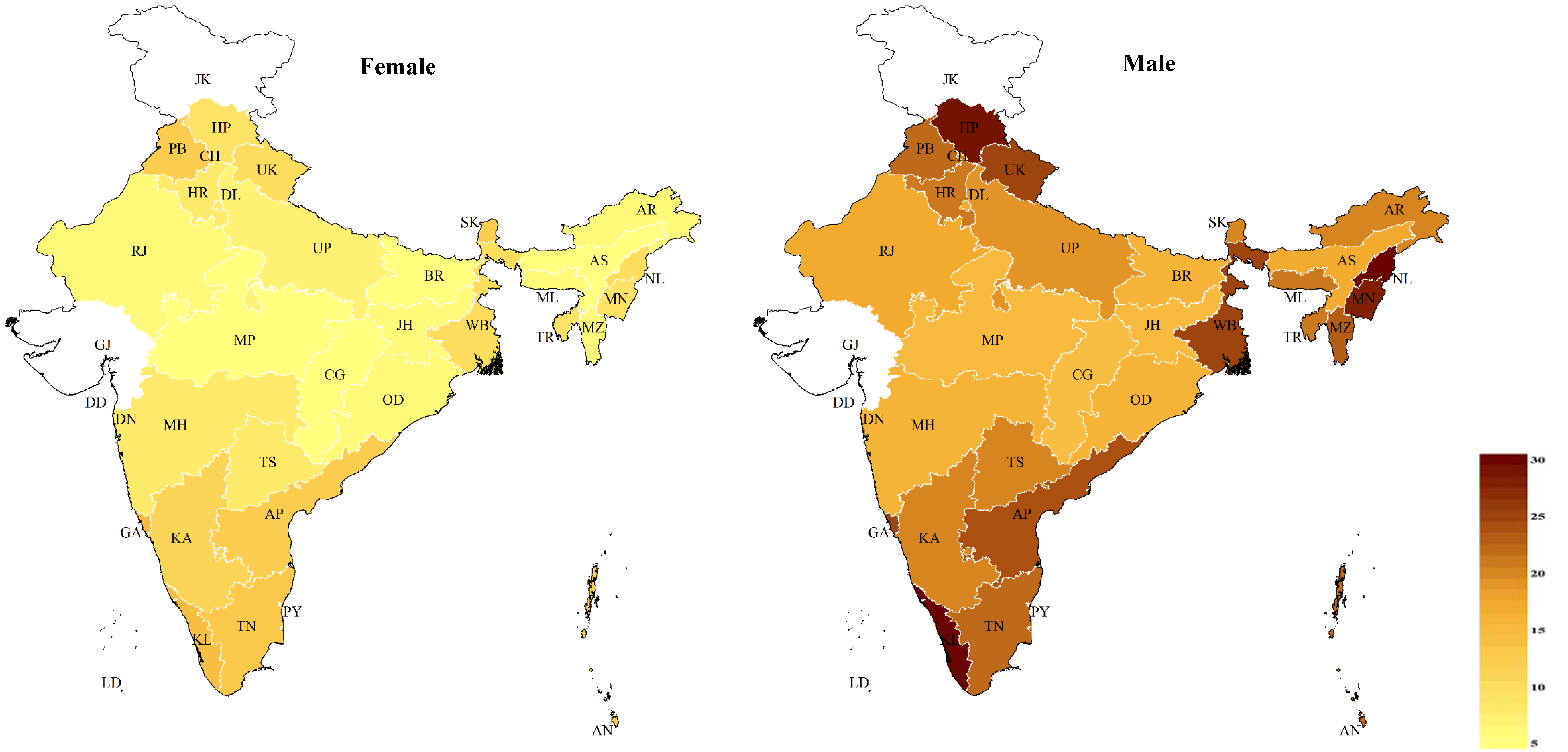
^

^
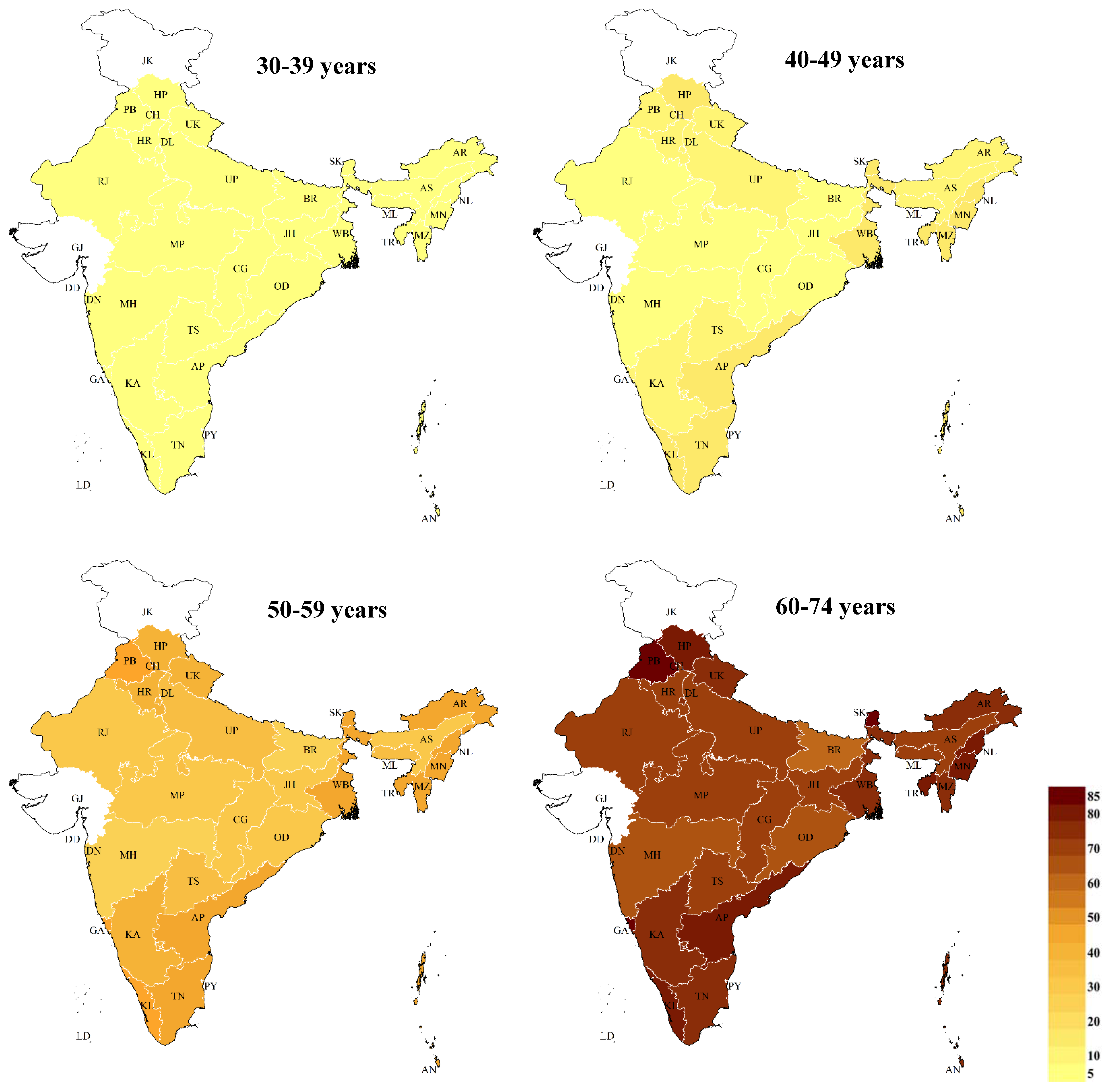
^

^
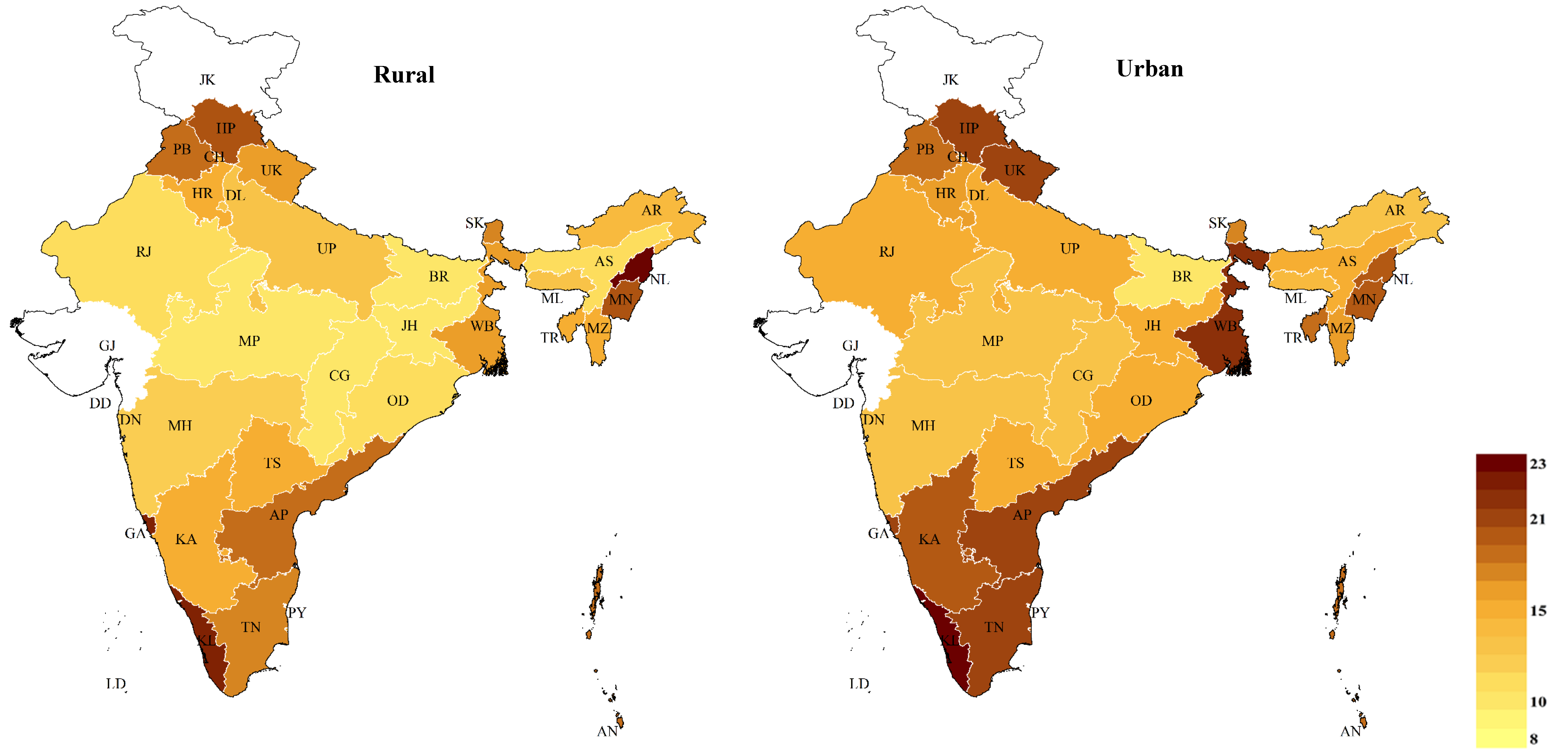
^

^1^ High CVD risk was defined as a 10-year cardiovascular disease risk ≥30% as calculated with the Framingham risk score.

^2^ The Global Burden of Disease Project’s 2013 population for India was used for age standardization.[1]

^3^ No data was available for Gujarat, and Jammu and Kashmir.

Abbreviations: AP indicates Andhra Pradesh; AR, Arunachal Pradesh; AS, Assam; BR, Bihar; CG, Chhattisgarh; CH, Chandigarh; DD, Daman and Diu; DL, Delhi; GA, Goa; GJ, Gujarat; HR, Haryana; HP, Himachal Pradesh; JH, Jharkhand; JK, Jammu and Kashmir; KA, Karnataka; KL, Kerala; MP, Madhya Pradesh; MH, Maharashtra; MN, Manipur; ML, Meghalaya; MZ, Mizoram; NL, Nagaland; OD, Odisha (Orissa); PB, Punjab; PY, Puducherry; RJ, Rajasthan; SK, Sikkim; TN, Tamil Nadu; TS, Telangana State; TR, Tripura; UP, Uttar Pradesh; UK, Uttarakhand (Uttaranchal); WB, West Bengal.

**Figure C. Age-standardized cardiovascular disease risk by state as calculated with each risk score**

Mean 10-year CVD risk using Harvard-NHANES^2,3^:

^
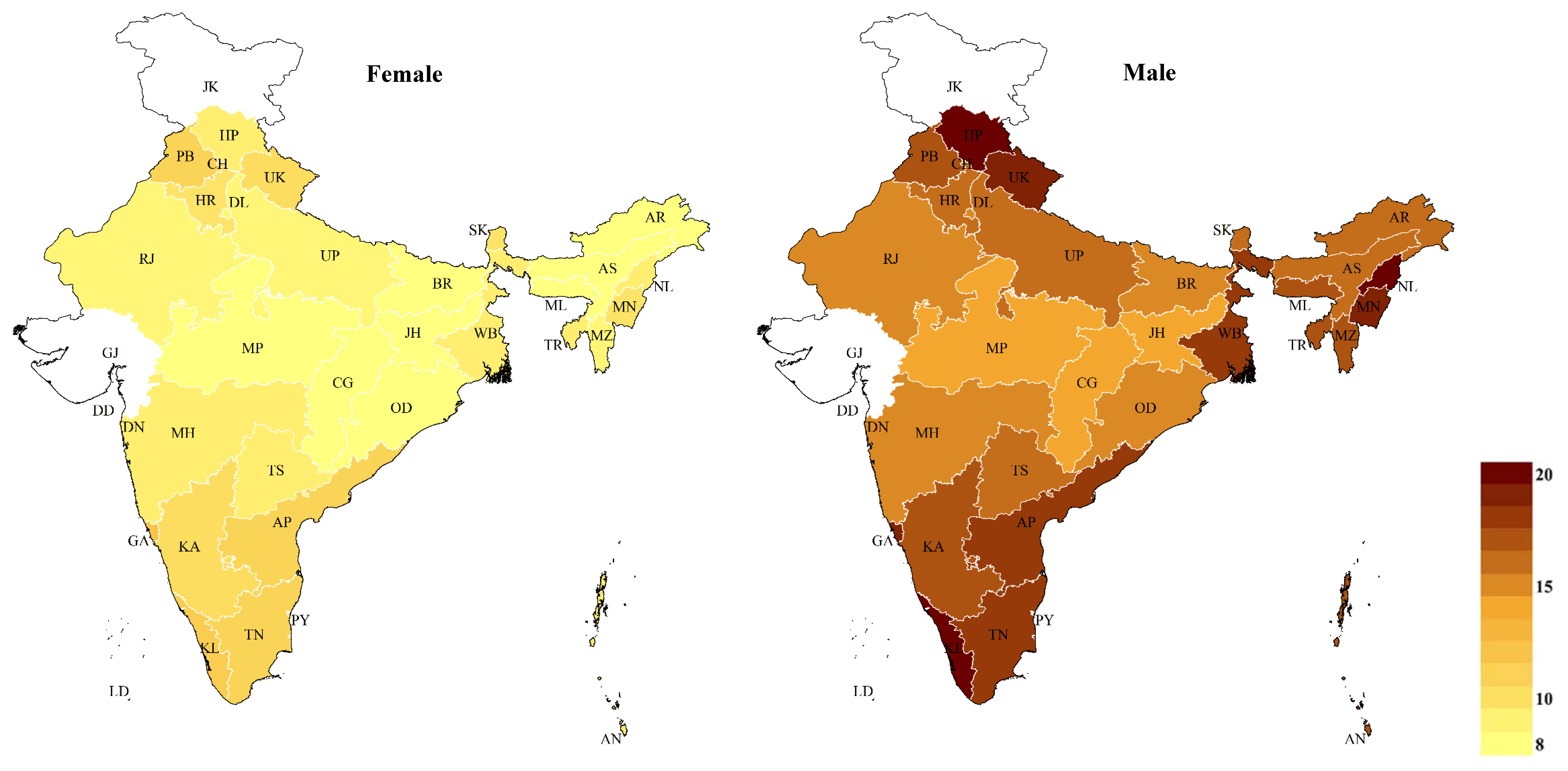
^

^2^ The Global Burden of Disease Project’s 2013 population for India was used for age standardization.[1]

^3^ No data was available for Gujarat, and Jammu and Kashmir.

Abbreviations: AP indicates Andhra Pradesh; AR, Arunachal Pradesh; AS, Assam; BR, Bihar; CG, Chhattisgarh; CH, Chandigarh; DD, Daman and Diu; DL, Delhi; GA, Goa; GJ, Gujarat; HR, Haryana; HP, Himachal Pradesh; JH, Jharkhand; JK, Jammu and Kashmir; KA, Karnataka; KL, Kerala; MP, Madhya Pradesh; MH, Maharashtra; MN, Manipur; ML, Meghalaya; MZ, Mizoram; NL, Nagaland; OD, Odisha (Orissa); PB, Punjab; PY, Puducherry; RJ, Rajasthan; SK, Sikkim; TN, Tamil Nadu; TS, Telangana State; TR, Tripura; UP, Uttar Pradesh; UK, Uttarakhand (Uttaranchal); WB, West Bengal.

Mean 10-year CVD risk using Harvard-NHANES^2,3^:

^
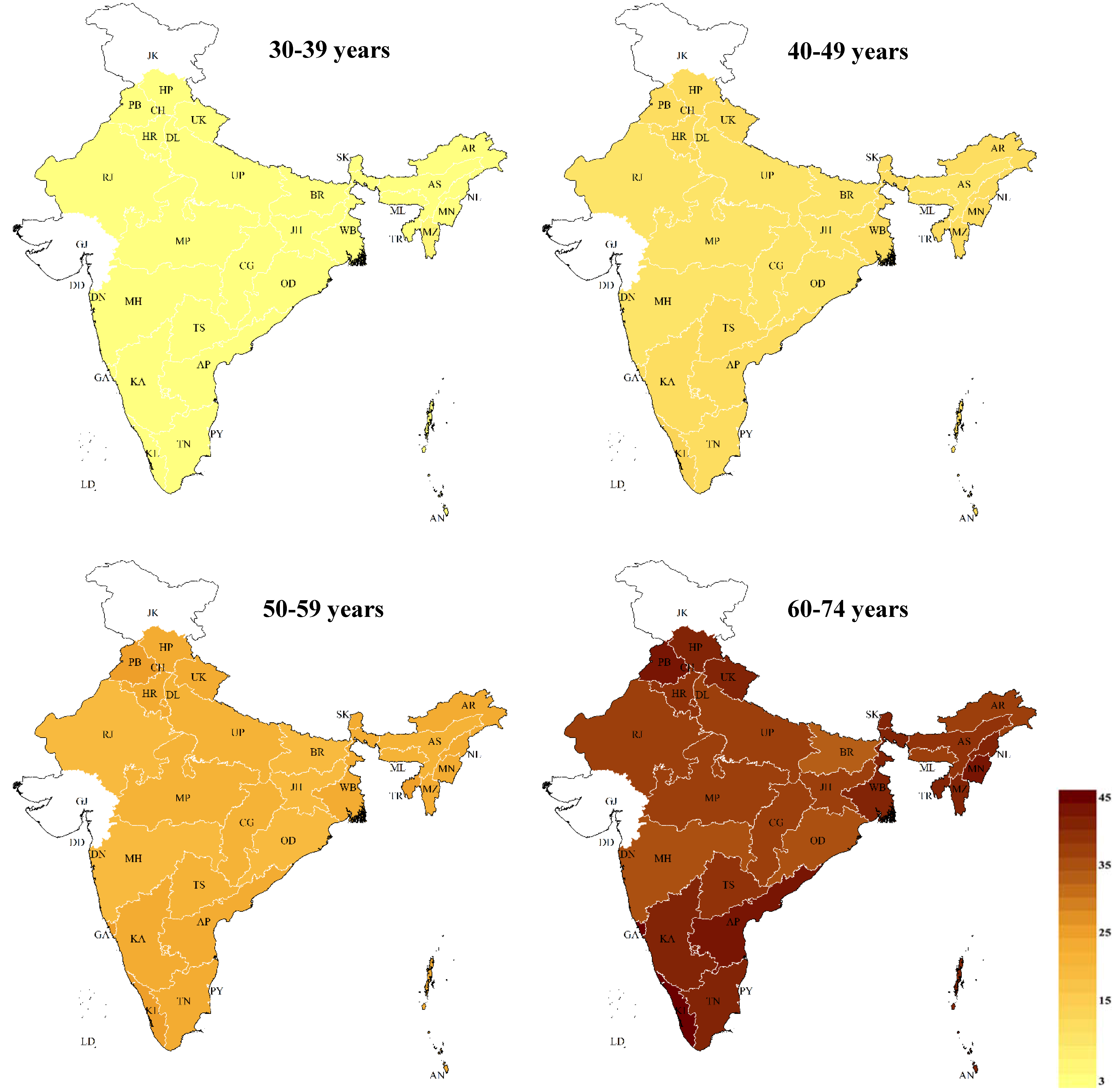
^

^2^ The Global Burden of Disease Project’s 2013 population for India was used for age standardization.[1]

^3^ No data was available for Gujarat, and Jammu and Kashmir.

Abbreviations: AP indicates Andhra Pradesh; AR, Arunachal Pradesh; AS, Assam; BR, Bihar; CG, Chhattisgarh; CH, Chandigarh; DD, Daman and Diu; DL, Delhi; GA, Goa; GJ, Gujarat; HR, Haryana; HP, Himachal Pradesh; JH, Jharkhand; JK, Jammu and Kashmir; KA, Karnataka; KL, Kerala; MP, Madhya Pradesh; MH, Maharashtra; MN, Manipur; ML, Meghalaya; MZ, Mizoram; NL, Nagaland; OD, Odisha (Orissa); PB, Punjab; PY, Puducherry; RJ, Rajasthan; SK, Sikkim; TN, Tamil Nadu; TS, Telangana State; TR, Tripura; UP, Uttar Pradesh; UK, Uttarakhand (Uttaranchal); WB, West Bengal.

Mean 10-year CVD risk using Harvard-NHANES^2,3^:

^
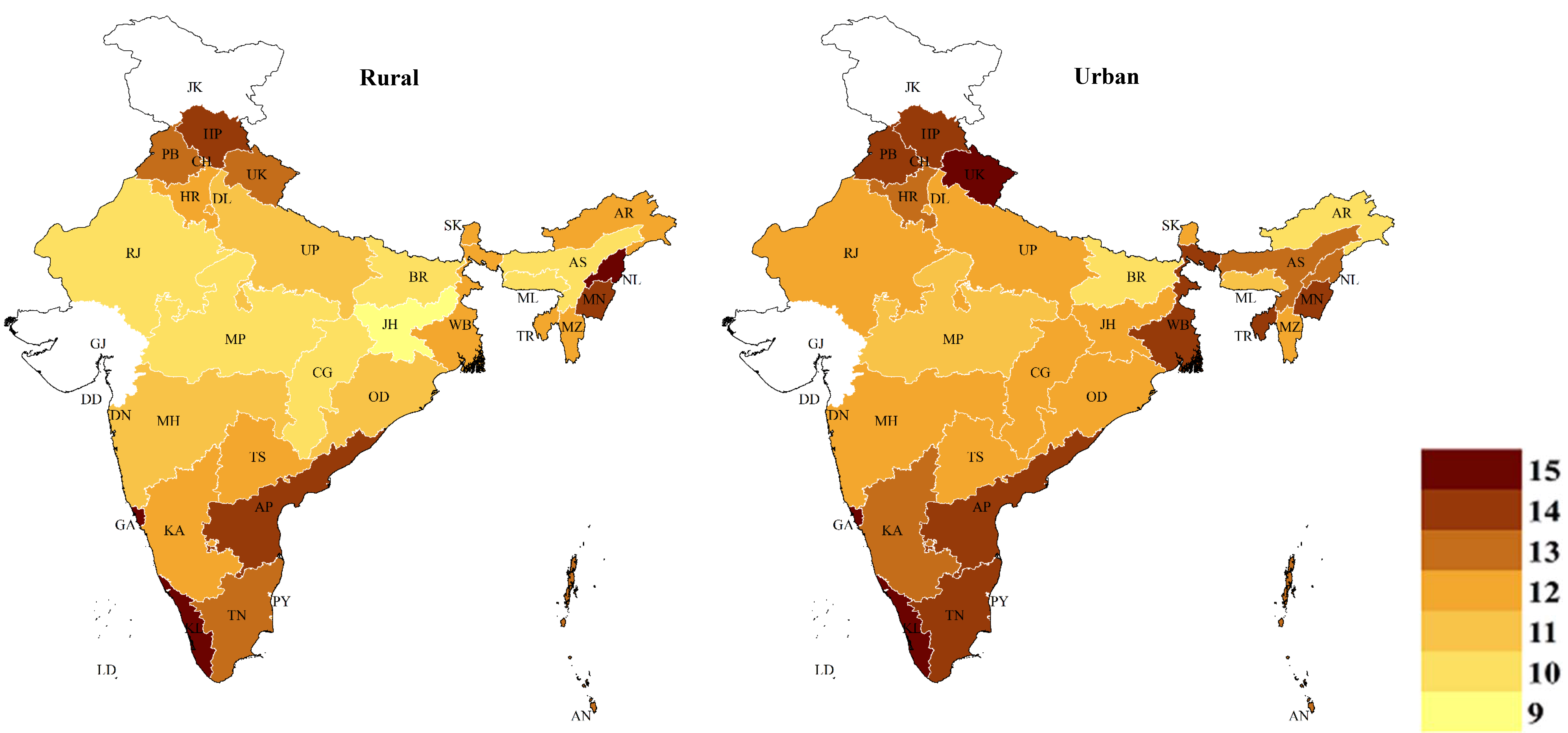
^

^2^ The Global Burden of Disease Project’s 2013 population for India was used for age standardization.[1]

^3^ No data was available for Gujarat, and Jammu and Kashmir.

Abbreviations: AP indicates Andhra Pradesh; AR, Arunachal Pradesh; AS, Assam; BR, Bihar; CG, Chhattisgarh; CH, Chandigarh; DD, Daman and Diu; DL, Delhi; GA, Goa; GJ, Gujarat; HR, Haryana; HP, Himachal Pradesh; JH, Jharkhand; JK, Jammu and Kashmir; KA, Karnataka; KL, Kerala; MP, Madhya Pradesh; MH, Maharashtra; MN, Manipur; ML, Meghalaya; MZ, Mizoram; NL, Nagaland; OD, Odisha (Orissa); PB, Punjab; PY, Puducherry; RJ, Rajasthan; SK, Sikkim; TN, Tamil Nadu; TS, Telangana State; TR, Tripura; UP, Uttar Pradesh; UK, Uttarakhand (Uttaranchal); WB, West Bengal.

Prevalence of a 10-year CVD risk ≥30% using Harvard-NHANES^2,3^:

^
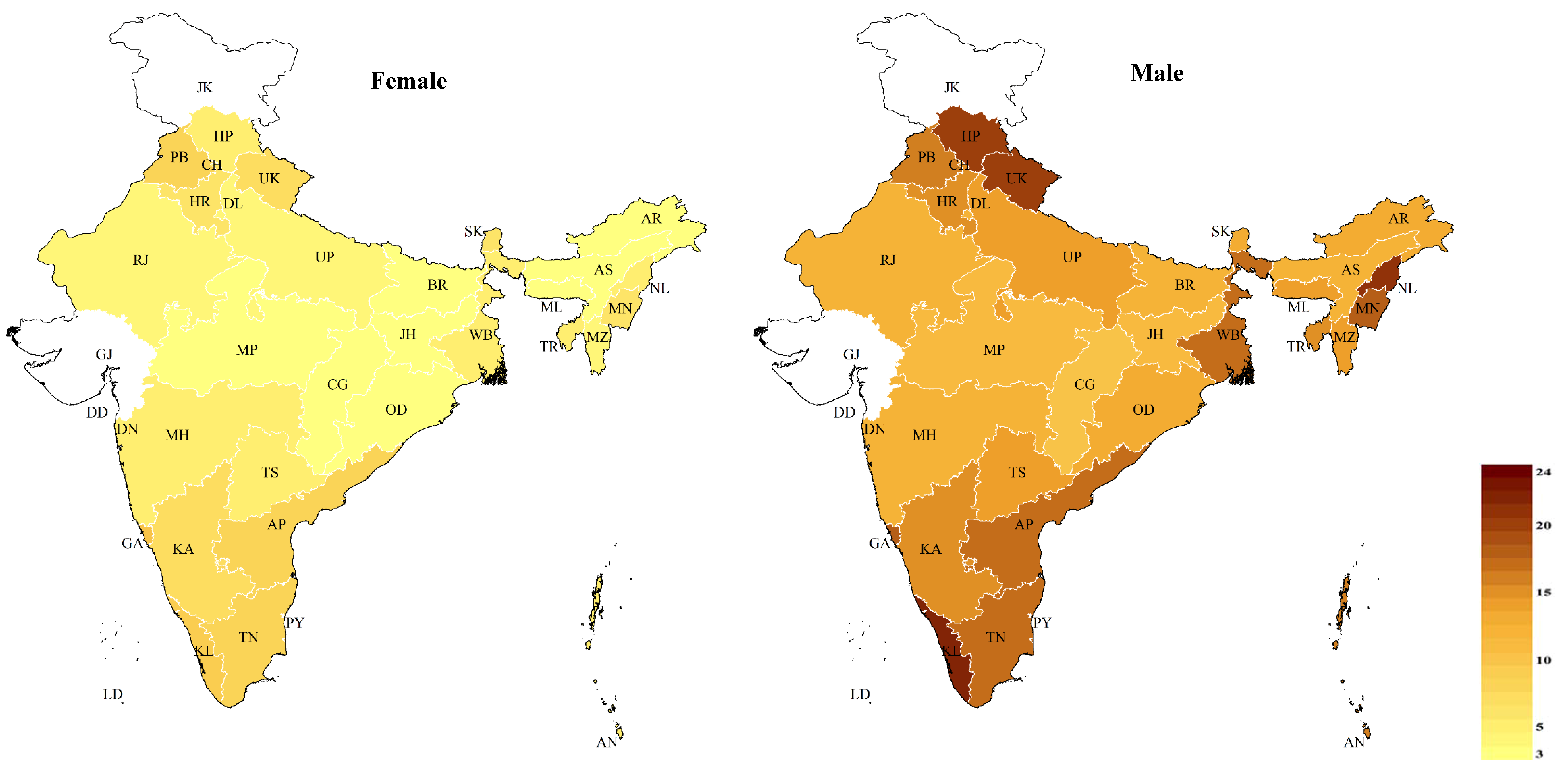
^

^2^ The Global Burden of Disease Project’s 2013 population for India was used for age standardization.[1]

^3^ No data was available for Gujarat, and Jammu and Kashmir.

Abbreviations: AP indicates Andhra Pradesh; AR, Arunachal Pradesh; AS, Assam; BR, Bihar; CG, Chhattisgarh; CH, Chandigarh; DD, Daman and Diu; DL, Delhi; GA, Goa; GJ, Gujarat; HR, Haryana; HP, Himachal Pradesh; JH, Jharkhand; JK, Jammu and Kashmir; KA, Karnataka; KL, Kerala; MP, Madhya Pradesh; MH, Maharashtra; MN, Manipur; ML, Meghalaya; MZ, Mizoram; NL, Nagaland; OD, Odisha (Orissa); PB, Punjab; PY, Puducherry; RJ, Rajasthan; SK, Sikkim; TN, Tamil Nadu; TS, Telangana State; TR, Tripura; UP, Uttar Pradesh; UK, Uttarakhand (Uttaranchal); WB, West Bengal.

Prevalence of a 10-year CVD risk ≥30% using Harvard-NHANES^2,3^:

^
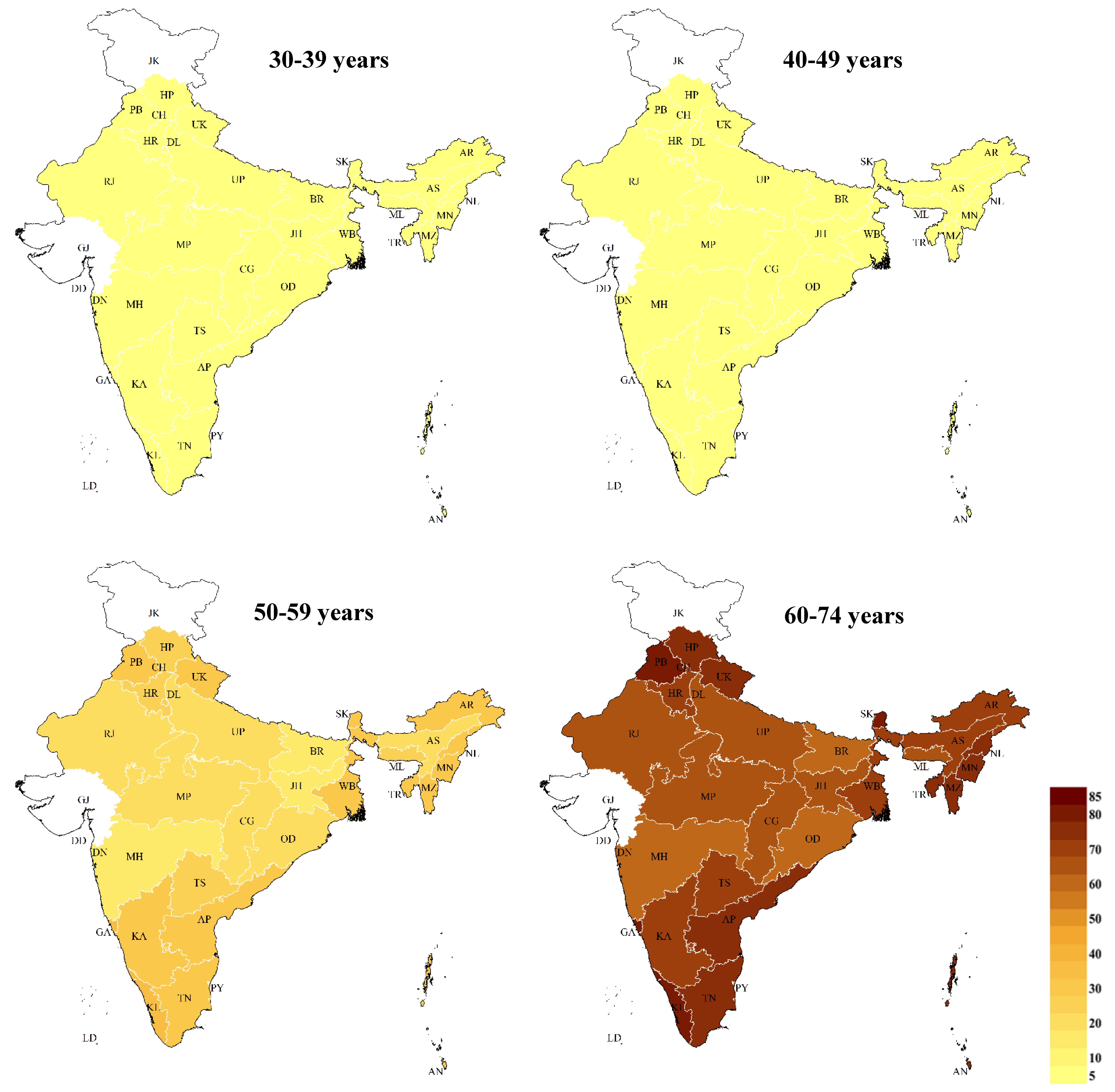
^

^2^ The Global Burden of Disease Project’s 2013 population for India was used for age standardization.[1]

^3^ No data was available for Gujarat, and Jammu and Kashmir.

Abbreviations: AP indicates Andhra Pradesh; AR, Arunachal Pradesh; AS, Assam; BR, Bihar; CG, Chhattisgarh; CH, Chandigarh; DD, Daman and Diu; DL, Delhi; GA, Goa; GJ, Gujarat; HR, Haryana; HP, Himachal Pradesh; JH, Jharkhand; JK, Jammu and Kashmir; KA, Karnataka; KL, Kerala; MP, Madhya Pradesh; MH, Maharashtra; MN, Manipur; ML, Meghalaya; MZ, Mizoram; NL, Nagaland; OD, Odisha (Orissa); PB, Punjab; PY, Puducherry; RJ, Rajasthan; SK, Sikkim; TN, Tamil Nadu; TS, Telangana State; TR, Tripura; UP, Uttar Pradesh; UK, Uttarakhand (Uttaranchal); WB, West Bengal.

Prevalence of a 10-year CVD risk ≥30% using Harvard-NHANES^2,3^:

^
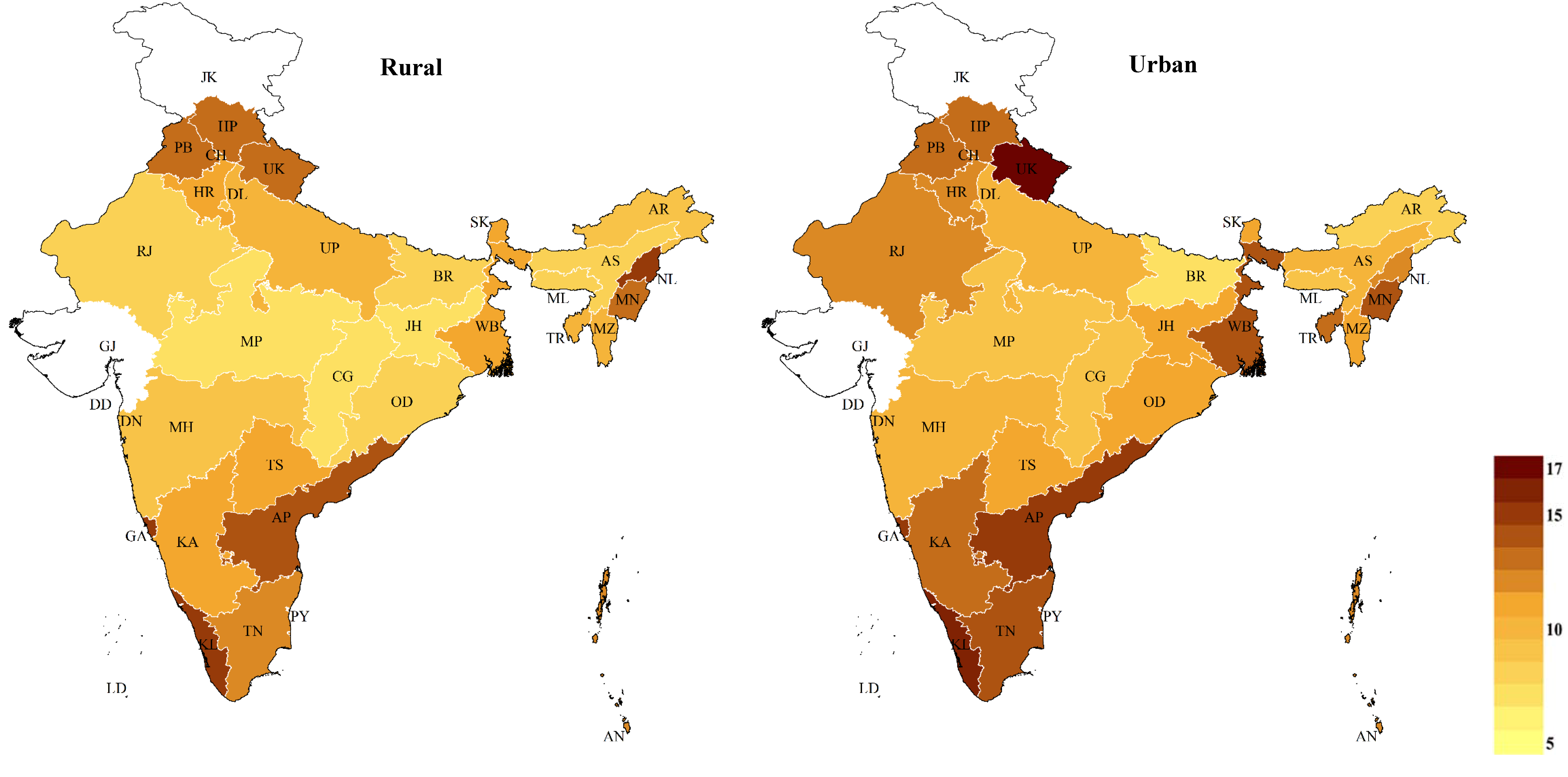
^

^2^ The Global Burden of Disease Project’s 2013 population for India was used for age standardization.[1]

^3^ No data was available for Gujarat, and Jammu and Kashmir.

Abbreviations: AP indicates Andhra Pradesh; AR, Arunachal Pradesh; AS, Assam; BR, Bihar; CG, Chhattisgarh; CH, Chandigarh; DD, Daman and Diu; DL, Delhi; GA, Goa; GJ, Gujarat; HR, Haryana; HP, Himachal Pradesh; JH, Jharkhand; JK, Jammu and Kashmir; KA, Karnataka; KL, Kerala; MP, Madhya Pradesh; MH, Maharashtra; MN, Manipur; ML, Meghalaya; MZ, Mizoram; NL, Nagaland; OD, Odisha (Orissa); PB, Punjab; PY, Puducherry; RJ, Rajasthan; SK, Sikkim; TN, Tamil Nadu; TS, Telangana State; TR, Tripura; UP, Uttar Pradesh; UK, Uttarakhand (Uttaranchal); WB, West Bengal.

Mean 10-year CVD risk using Globorisk^2,3^:

^
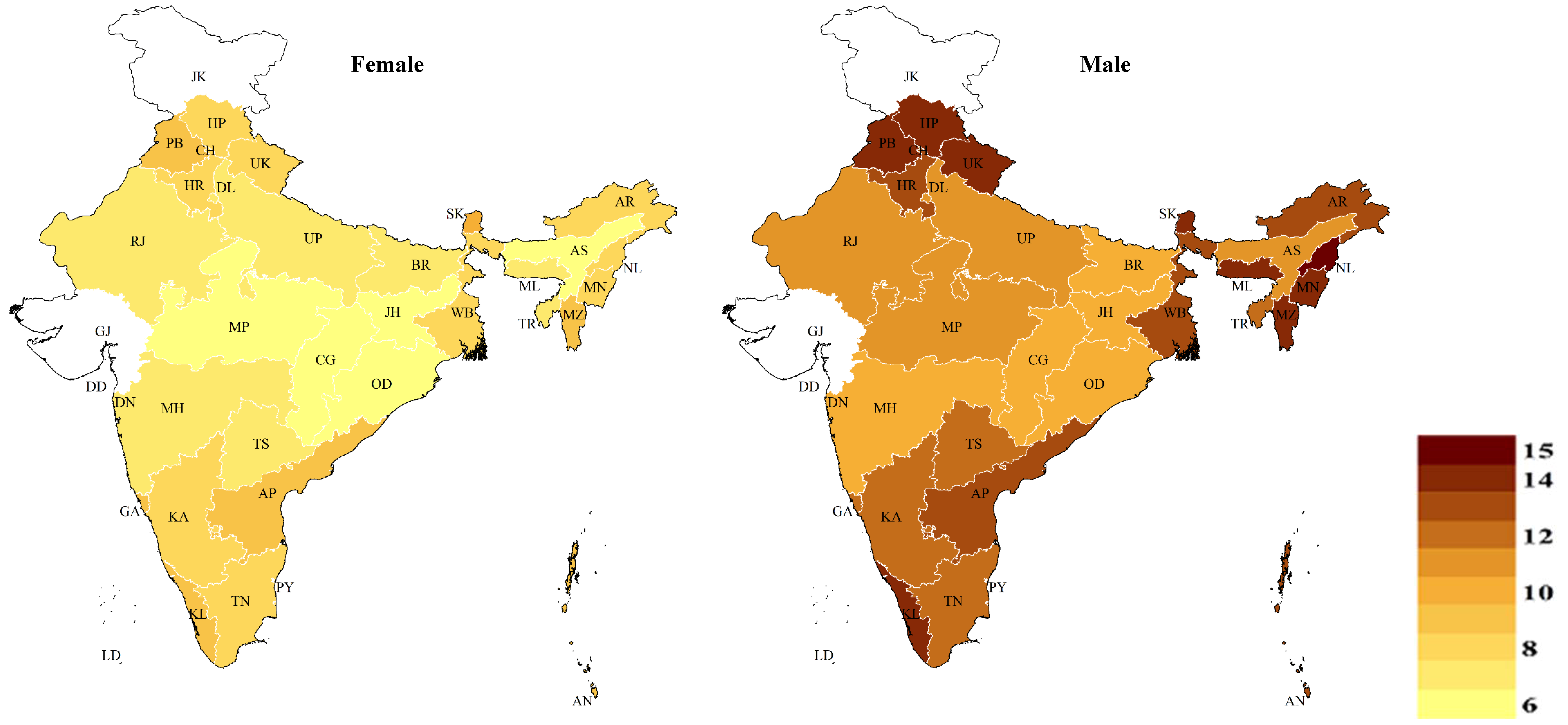
^

^2^ The Global Burden of Disease Project’s 2013 population for India was used for age standardization.[1]

^3^ No data was available for Gujarat, and Jammu and Kashmir.

Abbreviations: AP indicates Andhra Pradesh; AR, Arunachal Pradesh; AS, Assam; BR, Bihar; CG, Chhattisgarh; CH, Chandigarh; DD, Daman and Diu; DL, Delhi; GA, Goa; GJ, Gujarat; HR, Haryana; HP, Himachal Pradesh; JH, Jharkhand; JK, Jammu and Kashmir; KA, Karnataka; KL, Kerala; MP, Madhya Pradesh; MH, Maharashtra; MN, Manipur; ML, Meghalaya; MZ, Mizoram; NL, Nagaland; OD, Odisha (Orissa); PB, Punjab; PY, Puducherry; RJ, Rajasthan; SK, Sikkim; TN, Tamil Nadu; TS, Telangana State; TR, Tripura; UP, Uttar Pradesh; UK, Uttarakhand (Uttaranchal); WB, West Bengal.

Mean 10-year CVD risk using Globorisk^2,3^:

^
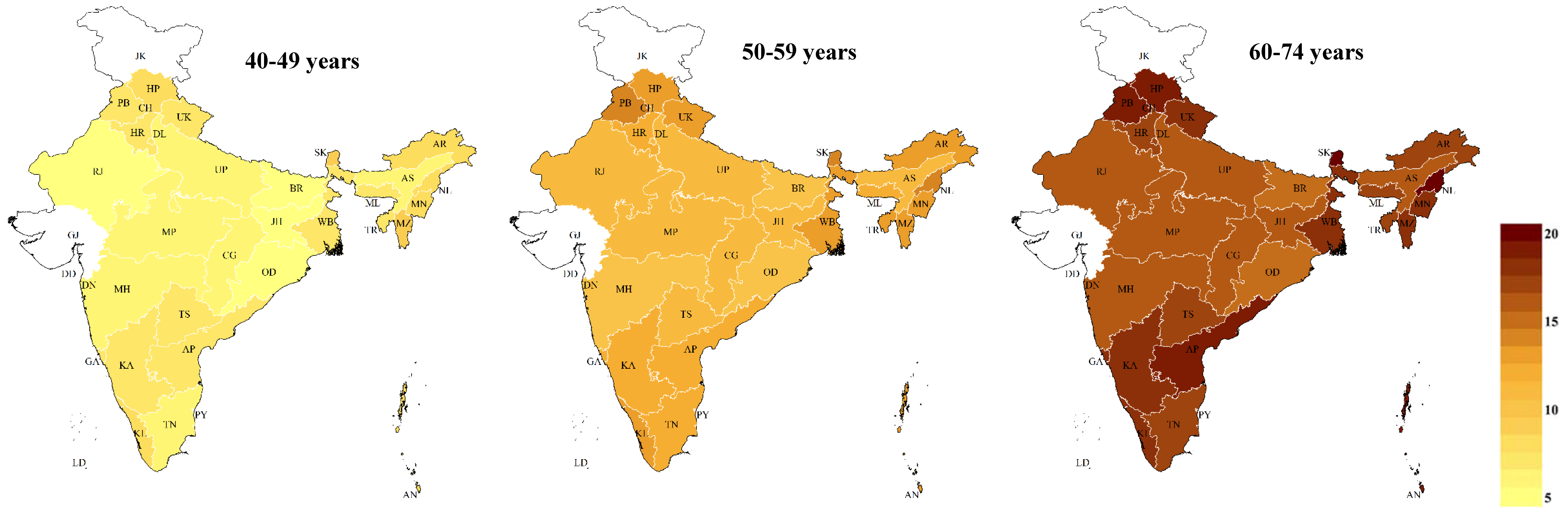
^

^2^ The Global Burden of Disease Project’s 2013 population for India was used for age standardization.[1]

^3^ No data was available for Gujarat, and Jammu and Kashmir.

Abbreviations: AP indicates Andhra Pradesh; AR, Arunachal Pradesh; AS, Assam; BR, Bihar; CG, Chhattisgarh; CH, Chandigarh; DD, Daman and Diu; DL, Delhi; GA, Goa; GJ, Gujarat; HR, Haryana; HP, Himachal Pradesh; JH, Jharkhand; JK, Jammu and Kashmir; KA, Karnataka; KL, Kerala; MP, Madhya Pradesh; MH, Maharashtra; MN, Manipur; ML, Meghalaya; MZ, Mizoram; NL, Nagaland; OD, Odisha (Orissa); PB, Punjab; PY, Puducherry; RJ, Rajasthan; SK, Sikkim; TN, Tamil Nadu; TS, Telangana State; TR, Tripura; UP, Uttar Pradesh; UK, Uttarakhand (Uttaranchal); WB, West Bengal.

Mean 10-year CVD risk using Globorisk^2,3^:

^
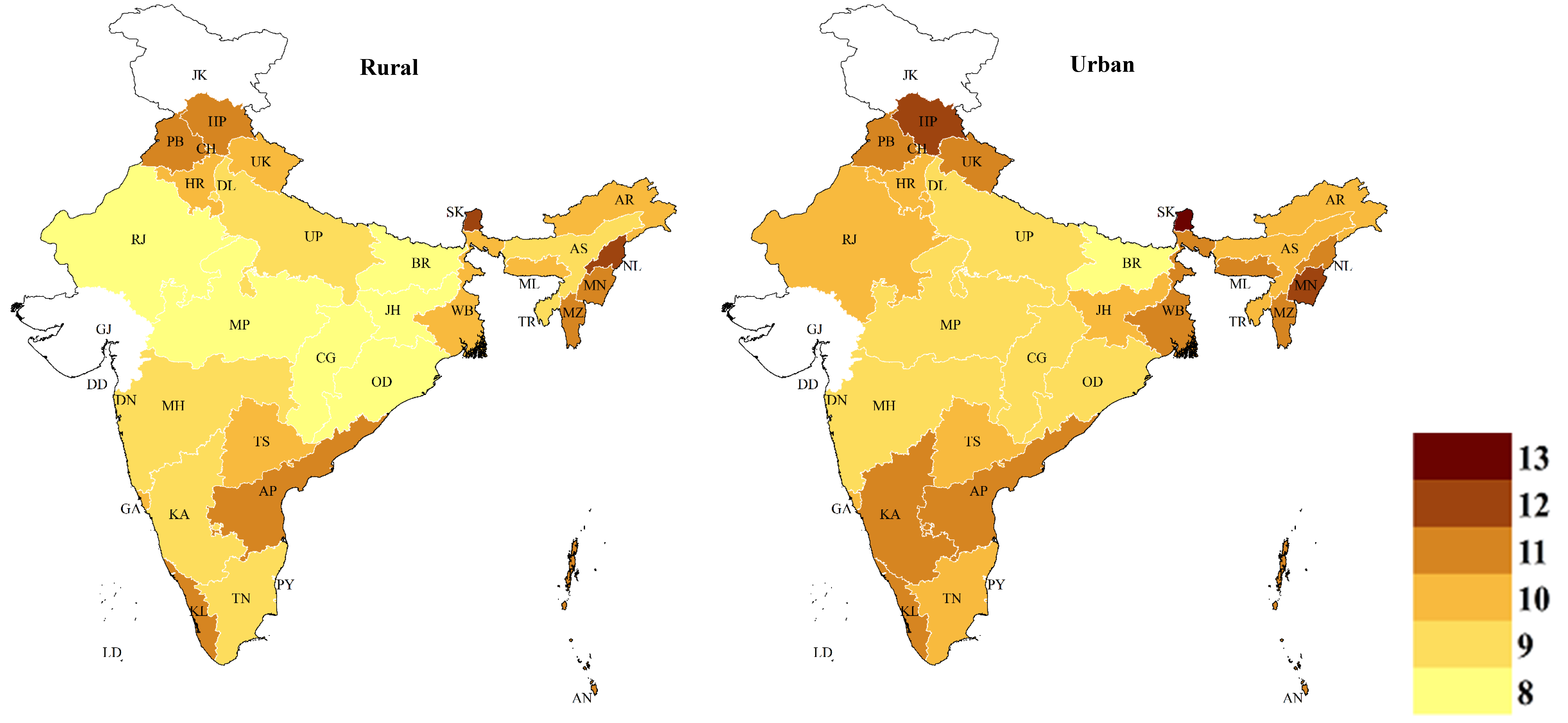
^

^2^ The Global Burden of Disease Project’s 2013 population for India was used for age standardization.[1]

^3^ No data was available for Gujarat, and Jammu and Kashmir.

Abbreviations: AP indicates Andhra Pradesh; AR, Arunachal Pradesh; AS, Assam; BR, Bihar; CG, Chhattisgarh; CH, Chandigarh; DD, Daman and Diu; DL, Delhi; GA, Goa; GJ, Gujarat; HR, Haryana; HP, Himachal Pradesh; JH, Jharkhand; JK, Jammu and Kashmir; KA, Karnataka; KL, Kerala; MP, Madhya Pradesh; MH, Maharashtra; MN, Manipur; ML, Meghalaya; MZ, Mizoram; NL, Nagaland; OD, Odisha (Orissa); PB, Punjab; PY, Puducherry; RJ, Rajasthan; SK, Sikkim; TN, Tamil Nadu; TS, Telangana State; TR, Tripura; UP, Uttar Pradesh; UK, Uttarakhand (Uttaranchal); WB, West Bengal.

Prevalence of a 10-year CVD risk ≥30% using Globorisk^2,3^:

^
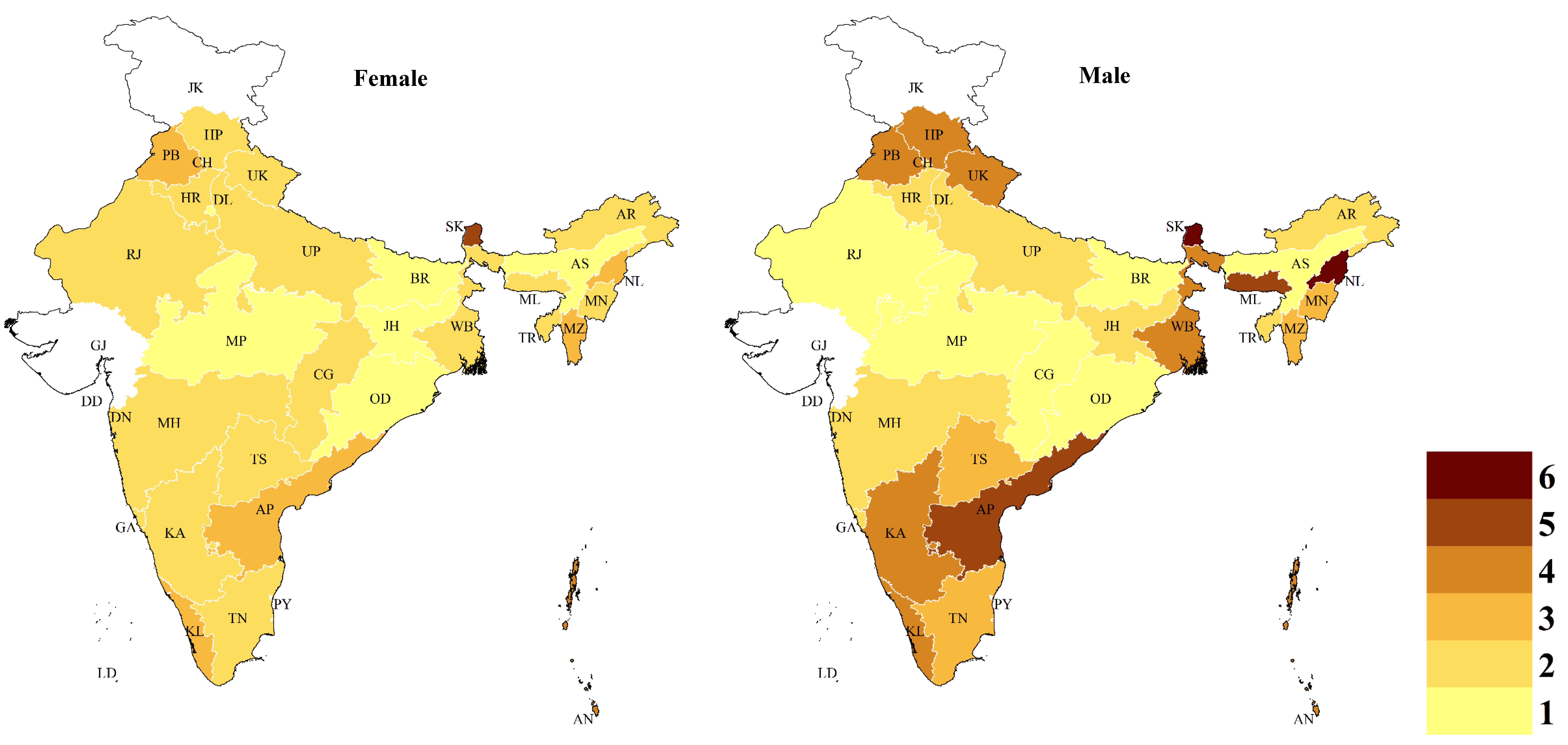
^

^2^ The Global Burden of Disease Project’s 2013 population for India was used for age standardization.[1]

^3^ No data was available for Gujarat, and Jammu and Kashmir.

Abbreviations: AP indicates Andhra Pradesh; AR, Arunachal Pradesh; AS, Assam; BR, Bihar; CG, Chhattisgarh; CH, Chandigarh; DD, Daman and Diu; DL, Delhi; GA, Goa; GJ, Gujarat; HR, Haryana; HP, Himachal Pradesh; JH, Jharkhand; JK, Jammu and Kashmir; KA, Karnataka; KL, Kerala; MP, Madhya Pradesh; MH, Maharashtra; MN, Manipur; ML, Meghalaya; MZ, Mizoram; NL, Nagaland; OD, Odisha (Orissa); PB, Punjab; PY, Puducherry; RJ, Rajasthan; SK, Sikkim; TN, Tamil Nadu; TS, Telangana State; TR, Tripura; UP, Uttar Pradesh; UK, Uttarakhand (Uttaranchal); WB, West Bengal.

Prevalence of a 10-year CVD risk ≥30% using Globorisk^2,3^:

^
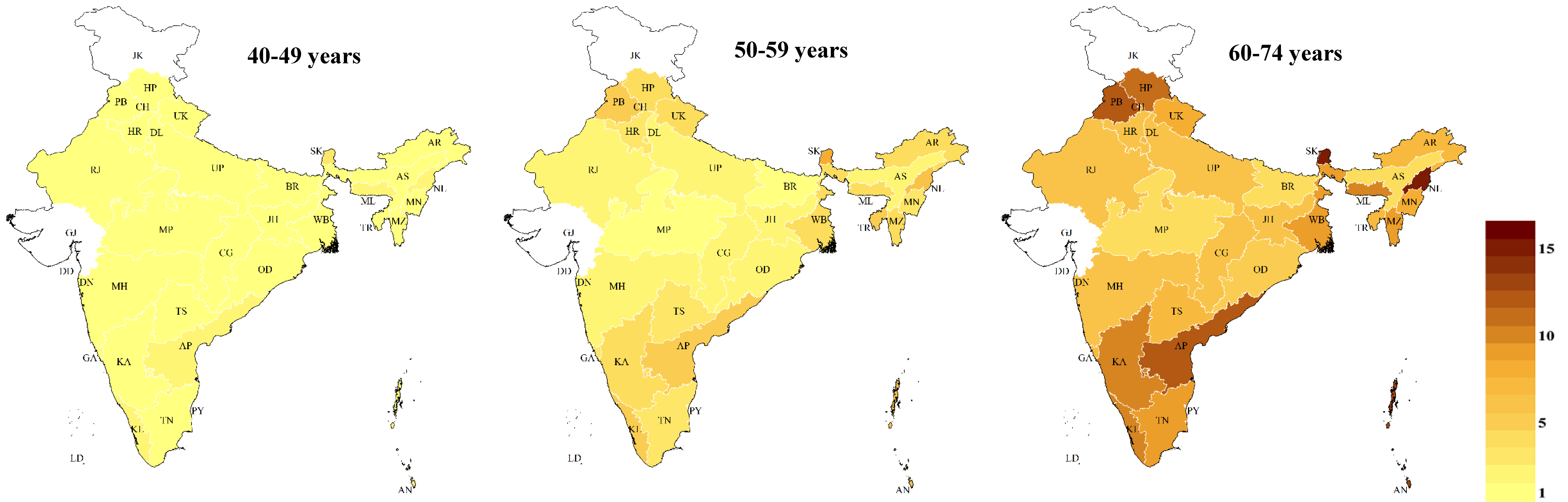
^

^2^ The Global Burden of Disease Project’s 2013 population for India was used for age standardization.[1]

^3^ No data was available for Gujarat, and Jammu and Kashmir.

Abbreviations: AP indicates Andhra Pradesh; AR, Arunachal Pradesh; AS, Assam; BR, Bihar; CG, Chhattisgarh; CH, Chandigarh; DD, Daman and Diu; DL, Delhi; GA, Goa; GJ, Gujarat; HR, Haryana; HP, Himachal Pradesh; JH, Jharkhand; JK, Jammu and Kashmir; KA, Karnataka; KL, Kerala; MP, Madhya Pradesh; MH, Maharashtra; MN, Manipur; ML, Meghalaya; MZ, Mizoram; NL, Nagaland; OD, Odisha (Orissa); PB, Punjab; PY, Puducherry; RJ, Rajasthan; SK, Sikkim; TN, Tamil Nadu; TS, Telangana State; TR, Tripura; UP, Uttar Pradesh; UK, Uttarakhand (Uttaranchal); WB, West Bengal.

Prevalence of a 10-year CVD risk ≥30% using Globorisk^2,3^:

^
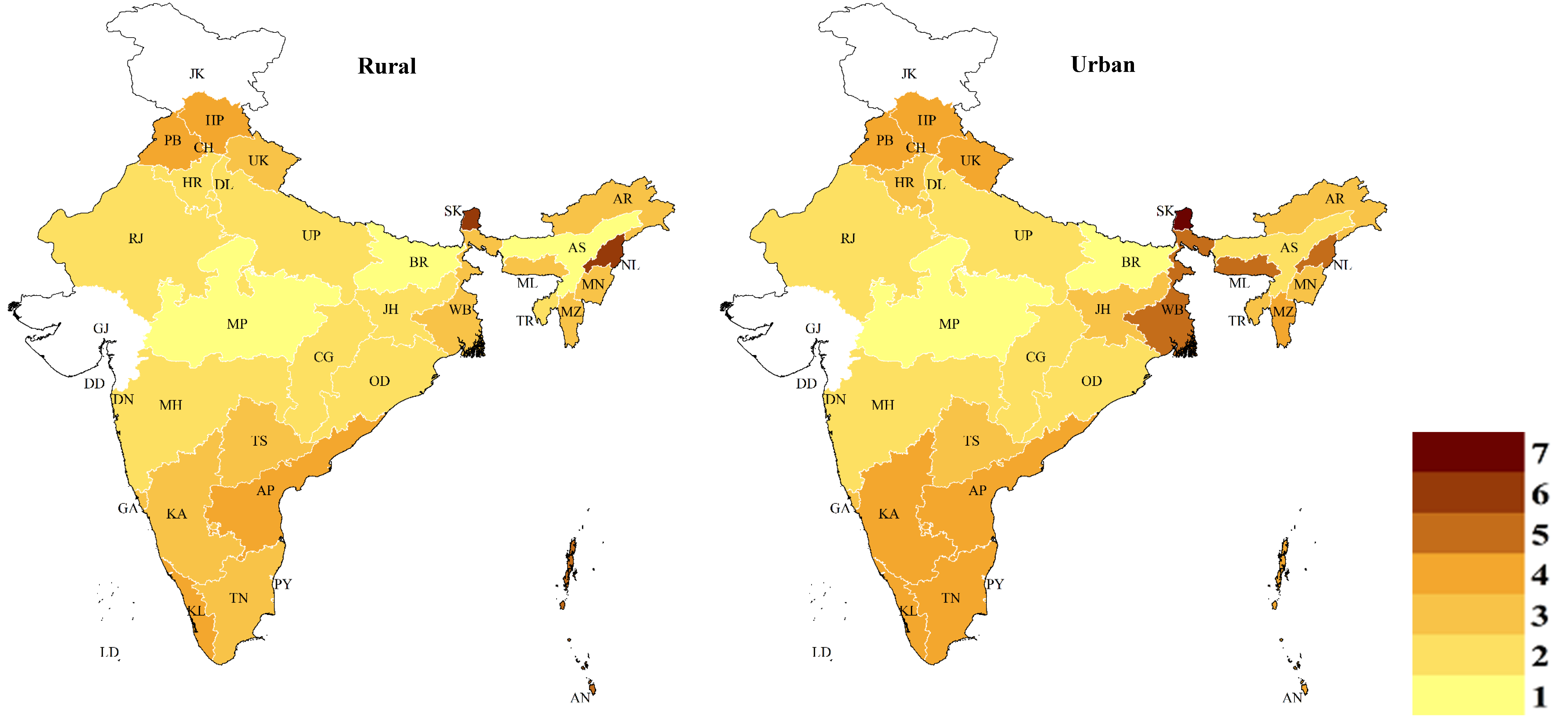
^

^2^ The Global Burden of Disease Project’s 2013 population for India was used for age standardization.[1]

^3^ No data was available for Gujarat, and Jammu and Kashmir.

Abbreviations: AP indicates Andhra Pradesh; AR, Arunachal Pradesh; AS, Assam; BR, Bihar; CG, Chhattisgarh; CH, Chandigarh; DD, Daman and Diu; DL, Delhi; GA, Goa; GJ, Gujarat; HR, Haryana; HP, Himachal Pradesh; JH, Jharkhand; JK, Jammu and Kashmir; KA, Karnataka; KL, Kerala; MP, Madhya Pradesh; MH, Maharashtra; MN, Manipur; ML, Meghalaya; MZ, Mizoram; NL, Nagaland; OD, Odisha (Orissa); PB, Punjab; PY, Puducherry; RJ, Rajasthan; SK, Sikkim; TN, Tamil Nadu; TS, Telangana State; TR, Tripura; UP, Uttar Pradesh; UK, Uttarakhand (Uttaranchal); WB, West Bengal.

Prevalence of a 10-year CVD risk ≥30% using WHO-ISH^2,3^:

^
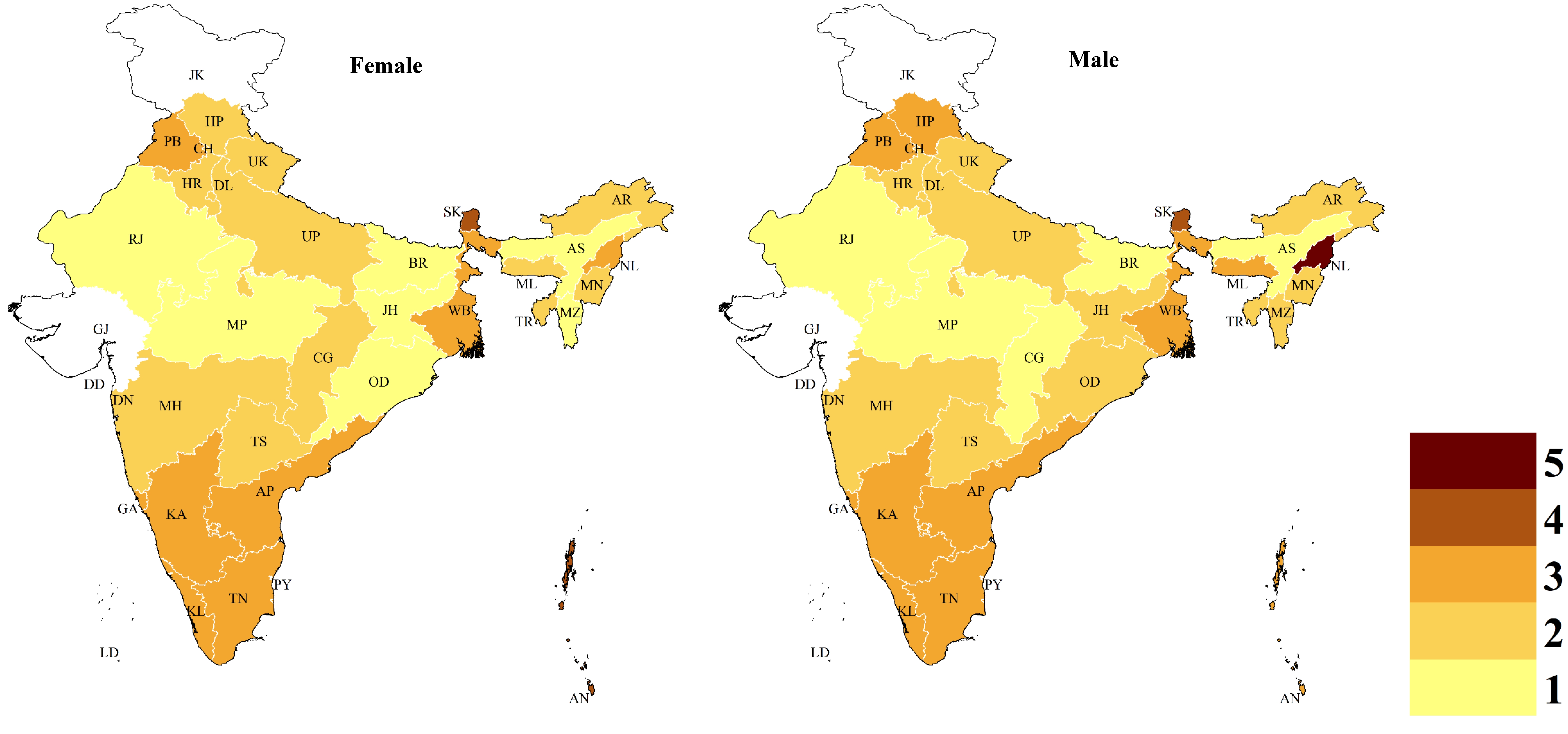
^

^2^ The Global Burden of Disease Project’s 2013 population for India was used for age standardization.[1]

^3^ No data was available for Gujarat, and Jammu and Kashmir.

Abbreviations: AP indicates Andhra Pradesh; AR, Arunachal Pradesh; AS, Assam; BR, Bihar; CG, Chhattisgarh; CH, Chandigarh; DD, Daman and Diu; DL, Delhi; GA, Goa; GJ, Gujarat; HR, Haryana; HP, Himachal Pradesh; JH, Jharkhand; JK, Jammu and Kashmir; KA, Karnataka; KL, Kerala; MP, Madhya Pradesh; MH, Maharashtra; MN, Manipur; ML, Meghalaya; MZ, Mizoram; NL, Nagaland; OD, Odisha (Orissa); PB, Punjab; PY, Puducherry; RJ, Rajasthan; SK, Sikkim; TN, Tamil Nadu; TS, Telangana State; TR, Tripura; UP, Uttar Pradesh; UK, Uttarakhand (Uttaranchal); WB, West Bengal.

Prevalence of a 10-year CVD risk ≥30% using WHO-ISH^2,3^:

^
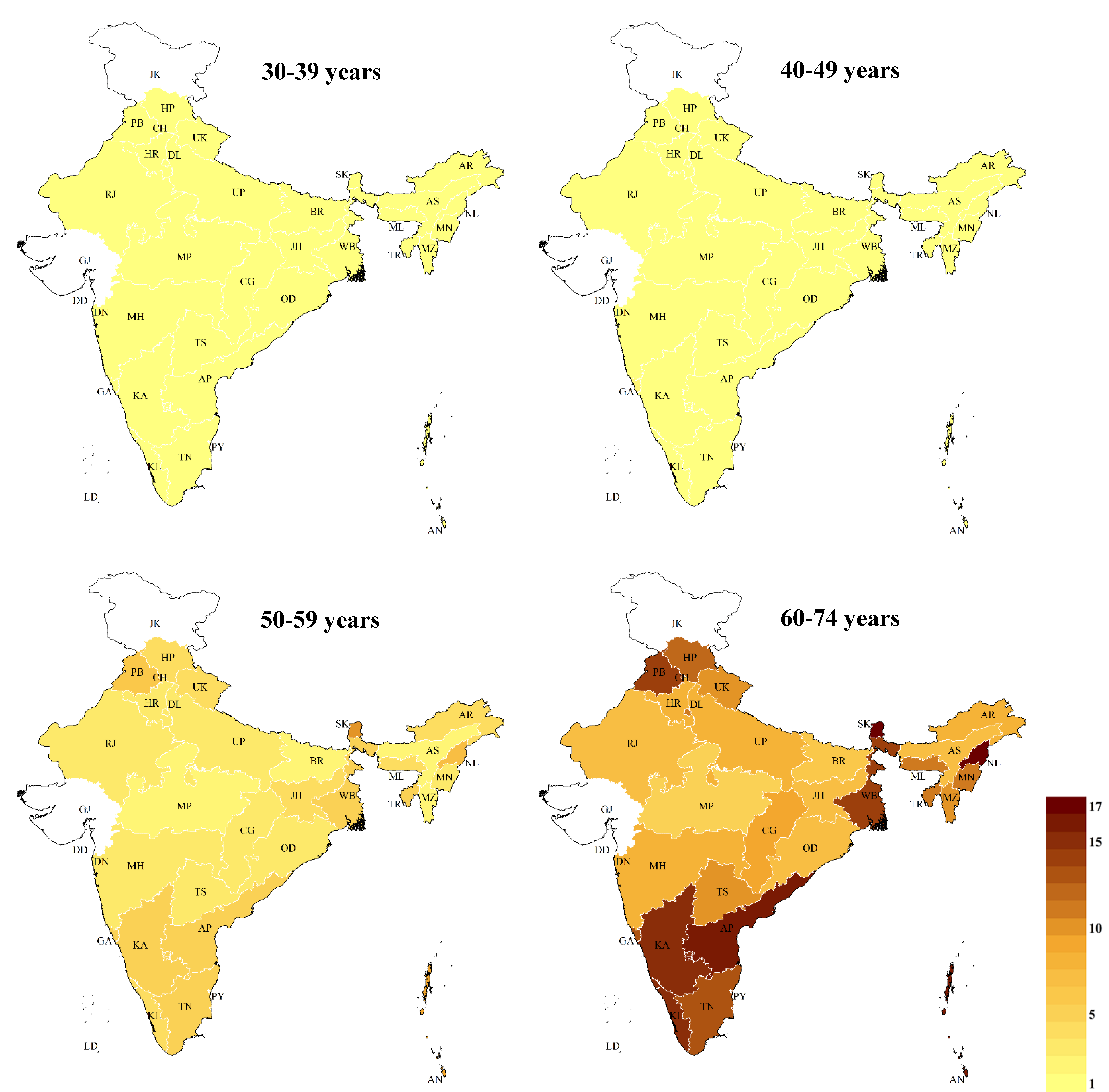
^

^2^ The Global Burden of Disease Project’s 2013 population for India was used for age standardization.[1]

^3^ No data was available for Gujarat, and Jammu and Kashmir.

Abbreviations: AP indicates Andhra Pradesh; AR, Arunachal Pradesh; AS, Assam; BR, Bihar; CG, Chhattisgarh; CH, Chandigarh; DD, Daman and Diu; DL, Delhi; GA, Goa; GJ, Gujarat; HR, Haryana; HP, Himachal Pradesh; JH, Jharkhand; JK, Jammu and Kashmir; KA, Karnataka; KL, Kerala; MP, Madhya Pradesh; MH, Maharashtra; MN, Manipur; ML, Meghalaya; MZ, Mizoram; NL, Nagaland; OD, Odisha (Orissa); PB, Punjab; PY, Puducherry; RJ, Rajasthan; SK, Sikkim; TN, Tamil Nadu; TS, Telangana State; TR, Tripura; UP, Uttar Pradesh; UK, Uttarakhand (Uttaranchal); WB, West Bengal.

Prevalence of a 10-year CVD risk ≥30% using WHO-ISH^2,3^:

^
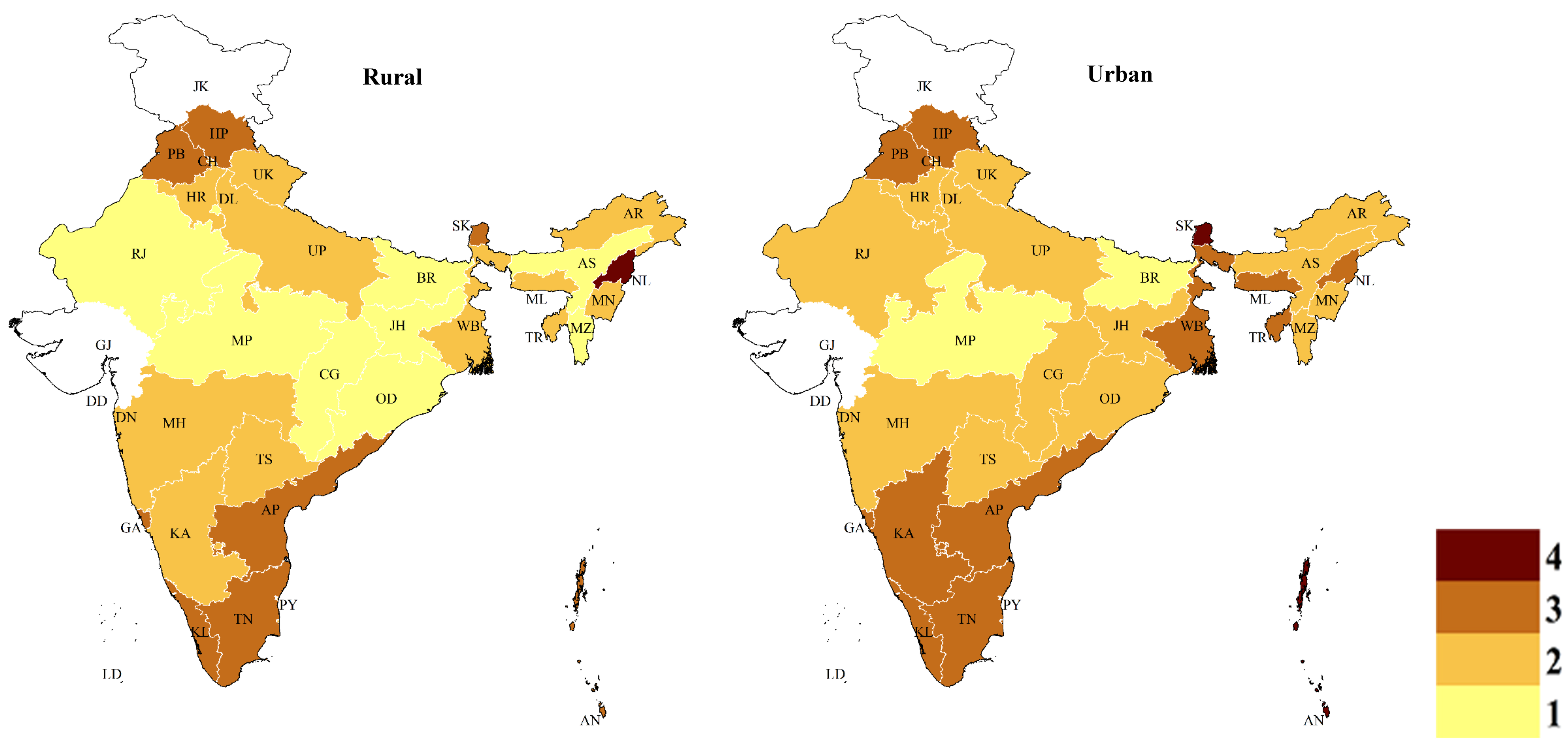
^

^2^ The Global Burden of Disease Project’s 2013 population for India was used for age standardization.[1]

^3^ No data was available for Gujarat, and Jammu and Kashmir.

Abbreviations: AP indicates Andhra Pradesh; AR, Arunachal Pradesh; AS, Assam; BR, Bihar; CG, Chhattisgarh; CH, Chandigarh; DD, Daman and Diu; DL, Delhi; GA, Goa; GJ, Gujarat; HR, Haryana; HP, Himachal Pradesh; JH, Jharkhand; JK, Jammu and Kashmir; KA, Karnataka; KL, Kerala; MP, Madhya Pradesh; MH, Maharashtra; MN, Manipur; ML, Meghalaya; MZ, Mizoram; NL, Nagaland; OD, Odisha (Orissa); PB, Punjab; PY, Puducherry; RJ, Rajasthan; SK, Sikkim; TN, Tamil Nadu; TS, Telangana State; TR, Tripura; UP, Uttar Pradesh; UK, Uttarakhand (Uttaranchal); WB, West Bengal.

**Figure D. Cardiovascular disease risk factors by state**

Age-standardized state-level mean body mass index, by age group^2,3^

^
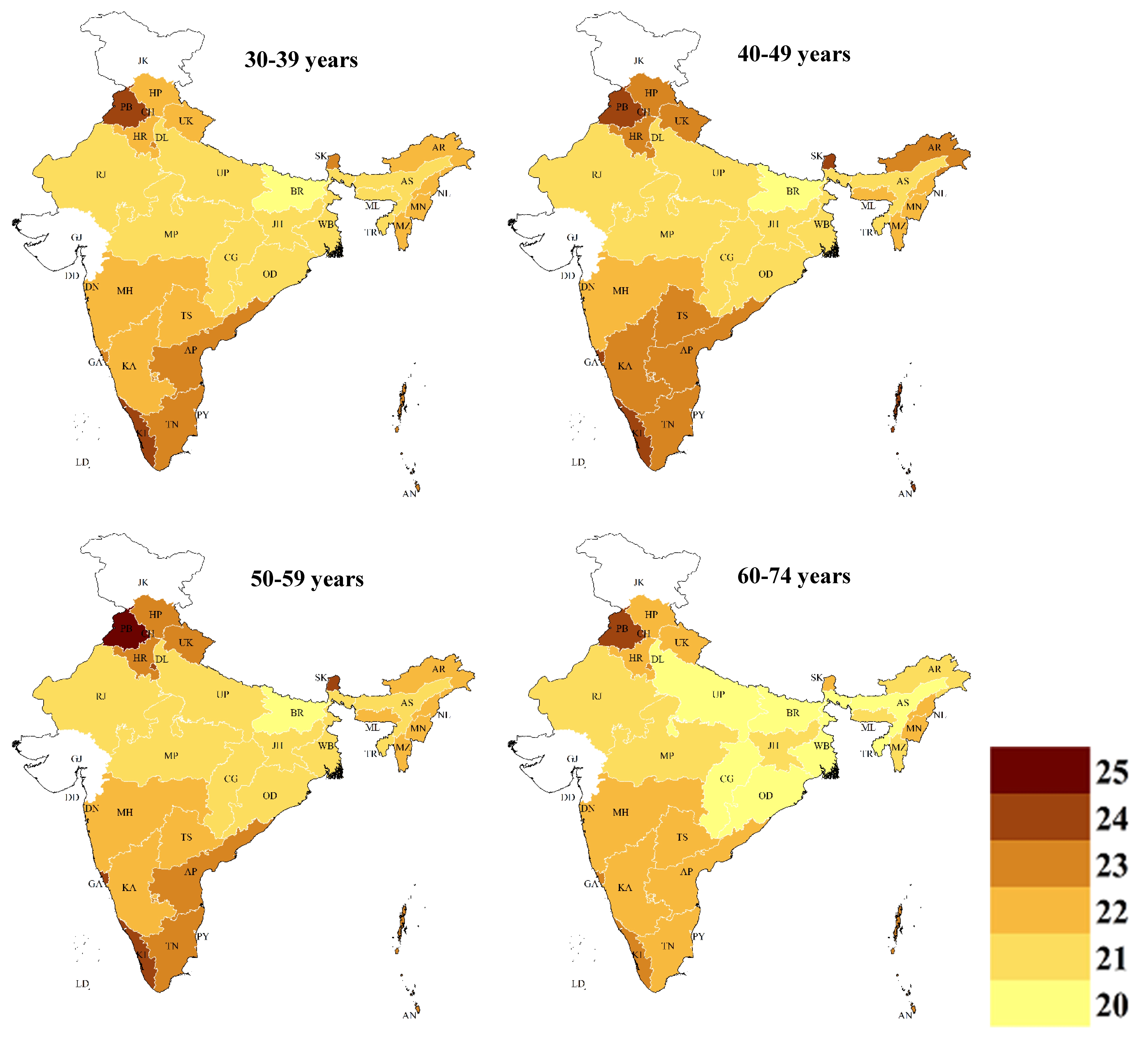
^

^2^ The Global Burden of Disease Project’s 2013 population for India was used for age standardization.[1]

^3^ No data was available for Gujarat, and Jammu and Kashmir.

Abbreviations: AP indicates Andhra Pradesh; AR, Arunachal Pradesh; AS, Assam; BR, Bihar; CG, Chhattisgarh; CH, Chandigarh; DD, Daman and Diu; DL, Delhi; GA, Goa; GJ, Gujarat; HR, Haryana; HP, Himachal Pradesh; JH, Jharkhand; JK, Jammu and Kashmir; KA, Karnataka; KL, Kerala; MP, Madhya Pradesh; MH, Maharashtra; MN, Manipur; ML, Meghalaya; MZ, Mizoram; NL, Nagaland; OD, Odisha (Orissa); PB, Punjab; PY, Puducherry; RJ, Rajasthan; SK, Sikkim; TN, Tamil Nadu; TS, Telangana State; TR, Tripura; UP, Uttar Pradesh; UK, Uttarakhand (Uttaranchal); WB, West Bengal.

Age-standardized state-level mean body mass index, by rural versus urban areas^2,3^

^
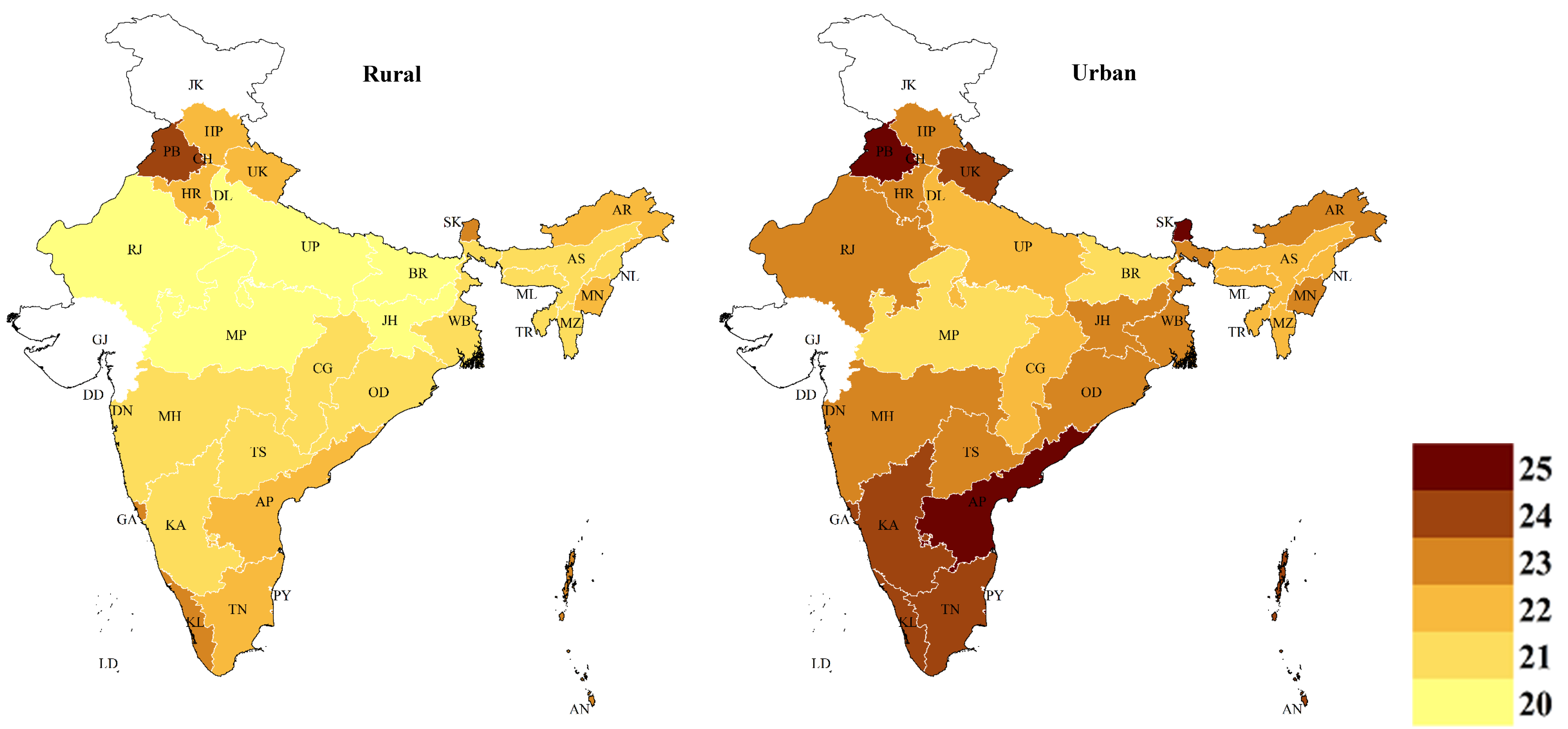
^

^2^ The Global Burden of Disease Project’s 2013 population for India was used for age standardization.[1]

^3^ No data was available for Gujarat, and Jammu and Kashmir.

Abbreviations: AP indicates Andhra Pradesh; AR, Arunachal Pradesh; AS, Assam; BR, Bihar; CG, Chhattisgarh; CH, Chandigarh; DD, Daman and Diu; DL, Delhi; GA, Goa; GJ, Gujarat; HR, Haryana; HP, Himachal Pradesh; JH, Jharkhand; JK, Jammu and Kashmir; KA, Karnataka; KL, Kerala; MP, Madhya Pradesh; MH, Maharashtra; MN, Manipur; ML, Meghalaya; MZ, Mizoram; NL, Nagaland; OD, Odisha (Orissa); PB, Punjab; PY, Puducherry; RJ, Rajasthan; SK, Sikkim; TN, Tamil Nadu; TS, Telangana State; TR, Tripura; UP, Uttar Pradesh; UK, Uttarakhand (Uttaranchal); WB, West Bengal.

Age-standardized state-level prevalence of a high blood glucose, by age group^2,3,4^

^
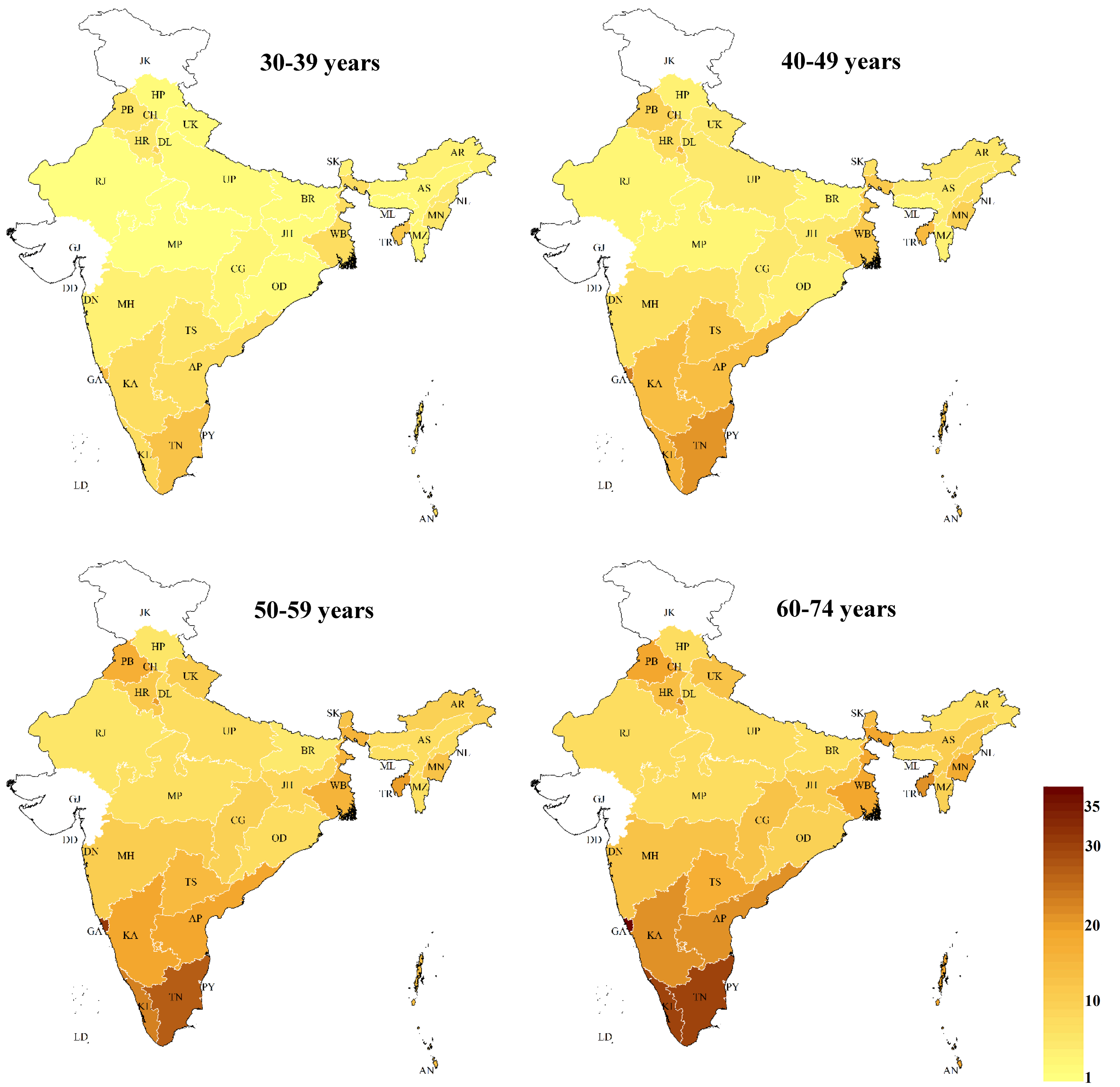
^

^2^ The Global Burden of Disease Project’s 2013 population for India was used for age standardization.[1]

^3^ No data was available for Gujarat, and Jammu and Kashmir.

^4^ ‘High blood glucose’ was defined as a high capillary blood glucose measurement (≥126mg/dl if fasted and ≥200mg/dl if non-fasted) or reporting to be on regular treatment for diabetes.

Abbreviations: AP indicates Andhra Pradesh; AR, Arunachal Pradesh; AS, Assam; BR, Bihar; CG, Chhattisgarh; CH, Chandigarh; DD, Daman and Diu; DL, Delhi; GA, Goa; GJ, Gujarat; HR, Haryana; HP, Himachal Pradesh; JH, Jharkhand; JK, Jammu and Kashmir; KA, Karnataka; KL, Kerala; MP, Madhya Pradesh; MH, Maharashtra; MN, Manipur; ML, Meghalaya; MZ, Mizoram; NL, Nagaland; OD, Odisha (Orissa); PB, Punjab; PY, Puducherry; RJ, Rajasthan; SK, Sikkim; TN, Tamil Nadu; TS, Telangana State; TR, Tripura; UP, Uttar Pradesh; UK, Uttarakhand (Uttaranchal); WB, West Bengal.

Age-standardized state-level prevalence of a high blood glucose, by rural versus urban areas^2,3,4^

^
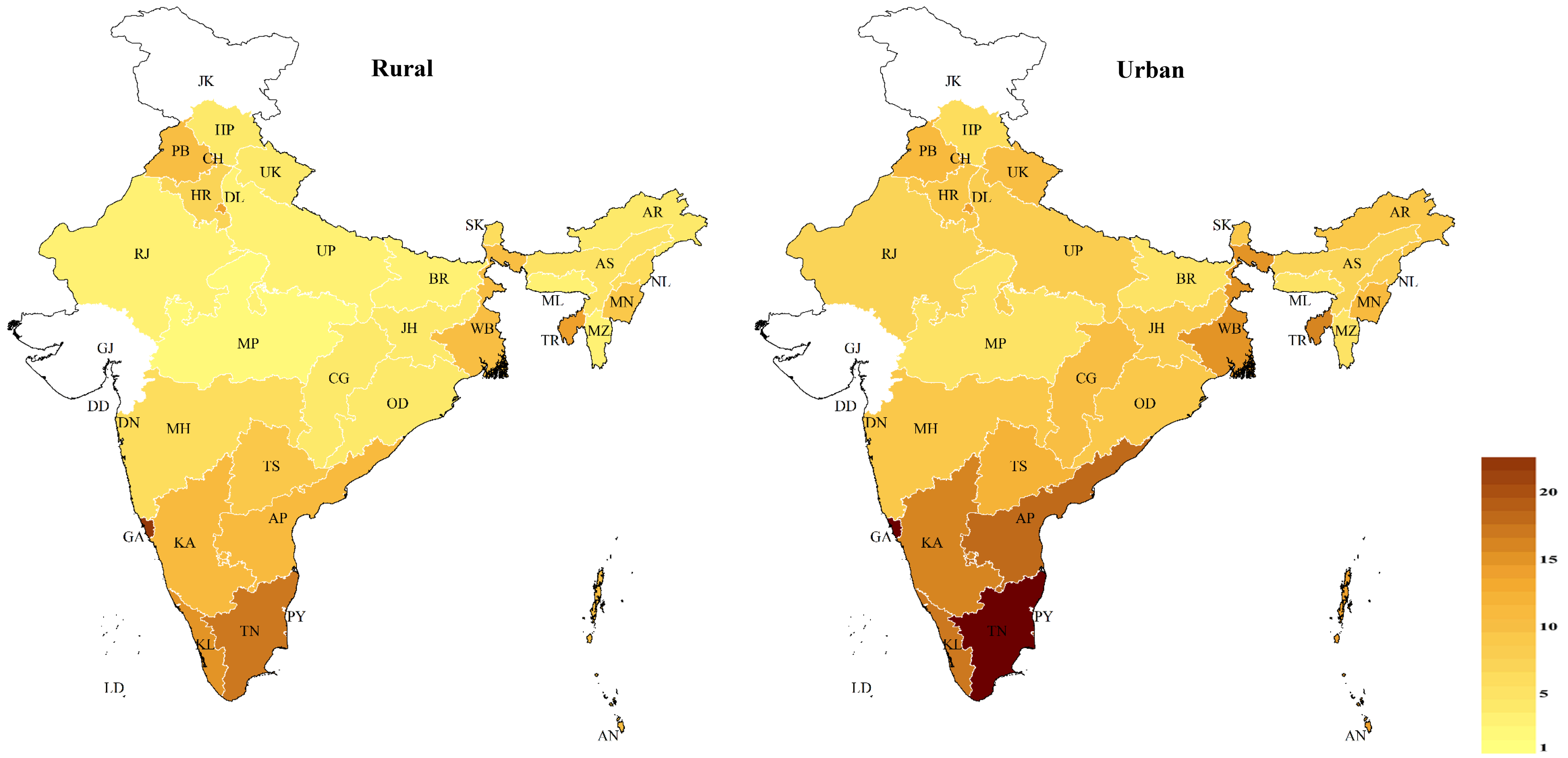
^

^2^ The Global Burden of Disease Project’s 2013 population for India was used for age standardization.[1]

^3^ No data was available for Gujarat, and Jammu and Kashmir.

^4^ ‘High blood glucose’ was defined as a high capillary blood glucose measurement (≥126mg/dl if fasted and ≥200mg/dl if non-fasted) or reporting to be on regular treatment for diabetes.

Abbreviations: AP indicates Andhra Pradesh; AR, Arunachal Pradesh; AS, Assam; BR, Bihar; CG, Chhattisgarh; CH, Chandigarh; DD, Daman and Diu; DL, Delhi; GA, Goa; GJ, Gujarat; HR, Haryana; HP, Himachal Pradesh; JH, Jharkhand; JK, Jammu and Kashmir; KA, Karnataka; KL, Kerala; MP, Madhya Pradesh; MH, Maharashtra; MN, Manipur; ML, Meghalaya; MZ, Mizoram; NL, Nagaland; OD, Odisha (Orissa); PB, Punjab; PY, Puducherry; RJ, Rajasthan; SK, Sikkim; TN, Tamil Nadu; TS, Telangana State; TR, Tripura; UP, Uttar Pradesh; UK, Uttarakhand (Uttaranchal); WB, West Bengal.

Age-standardized state-level prevalence of current smoking, by age group^2,3,5^

^
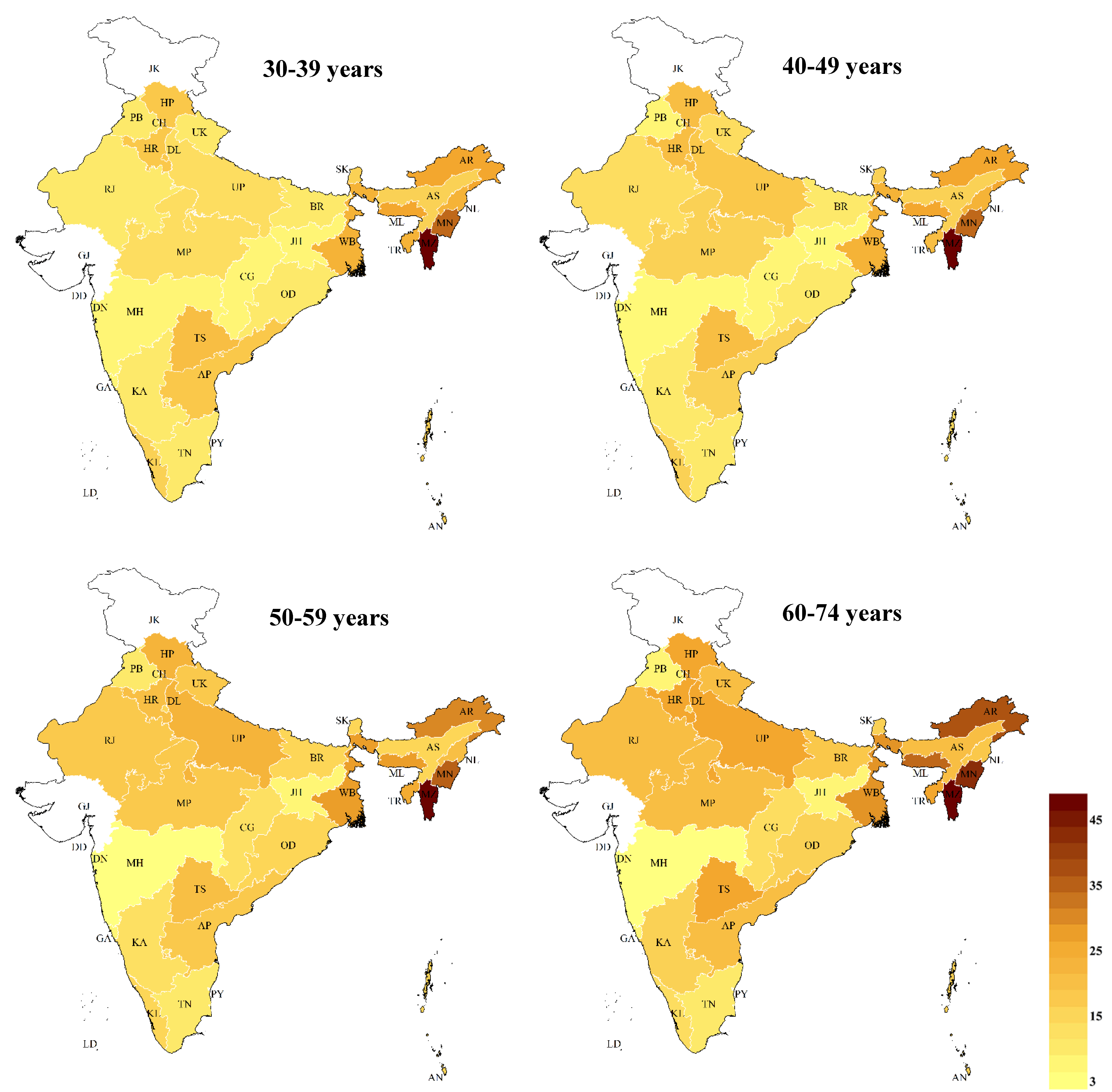
^

^2^ The Global Burden of Disease Project’s 2013 population for India was used for age standardization.[1]

^3^ No data was available for Gujarat, and Jammu and Kashmir.

^5^ ‘Smoking’ refers to smoking of any tobacco products but does not include chewing of tobacco.

Abbreviations: AP indicates Andhra Pradesh; AR, Arunachal Pradesh; AS, Assam; BR, Bihar; CG, Chhattisgarh; CH, Chandigarh; DD, Daman and Diu; DL, Delhi; GA, Goa; GJ, Gujarat; HR, Haryana; HP, Himachal Pradesh; JH, Jharkhand; JK, Jammu and Kashmir; KA, Karnataka; KL, Kerala; MP, Madhya Pradesh; MH, Maharashtra; MN, Manipur; ML, Meghalaya; MZ, Mizoram; NL, Nagaland; OD, Odisha (Orissa); PB, Punjab; PY, Puducherry; RJ, Rajasthan; SK, Sikkim; TN, Tamil Nadu; TS, Telangana State; TR, Tripura; UP, Uttar Pradesh; UK, Uttarakhand (Uttaranchal); WB, West Bengal.

Age-standardized state-level prevalence of current smoking, by rural versus urban areas^2,3,5^

^
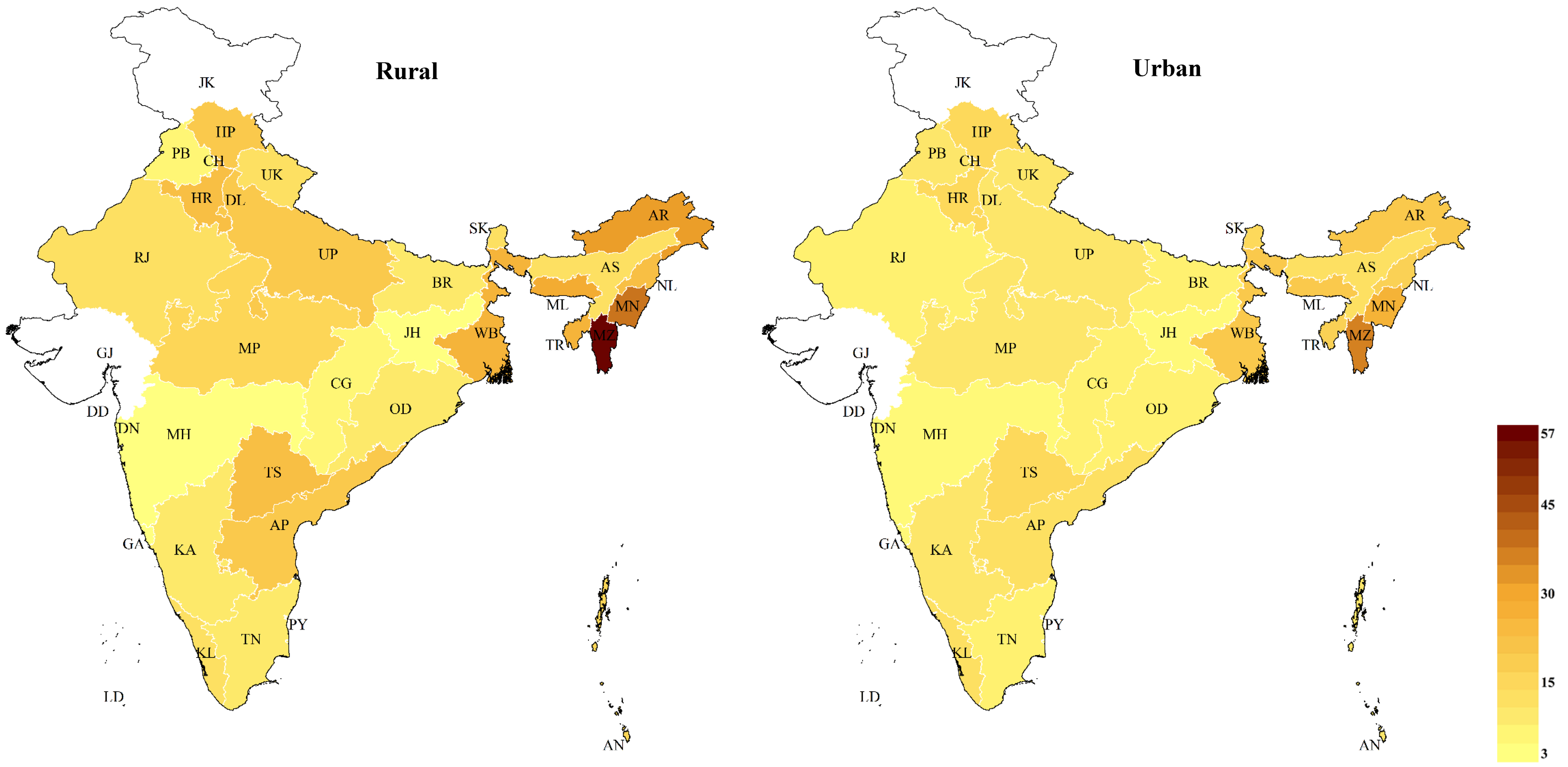
^

^2^ The Global Burden of Disease Project’s 2013 population for India was used for age standardization.[1]

^3^ No data was available for Gujarat, and Jammu and Kashmir.

^5^ ‘Smoking’ refers to smoking of any tobacco products but does not include chewing of tobacco.

Abbreviations: AP indicates Andhra Pradesh; AR, Arunachal Pradesh; AS, Assam; BR, Bihar; CG, Chhattisgarh; CH, Chandigarh; DD, Daman and Diu; DL, Delhi; GA, Goa; GJ, Gujarat; HR, Haryana; HP, Himachal Pradesh; JH, Jharkhand; JK, Jammu and Kashmir; KA, Karnataka; KL, Kerala; MP, Madhya Pradesh; MH, Maharashtra; MN, Manipur; ML, Meghalaya; MZ, Mizoram; NL, Nagaland; OD, Odisha (Orissa); PB, Punjab; PY, Puducherry; RJ, Rajasthan; SK, Sikkim; TN, Tamil Nadu; TS, Telangana State; TR, Tripura; UP, Uttar Pradesh; UK, Uttarakhand (Uttaranchal); WB, West Bengal.

Age-standardized state-level mean systolic blood pressure, by age group^2,3^

^
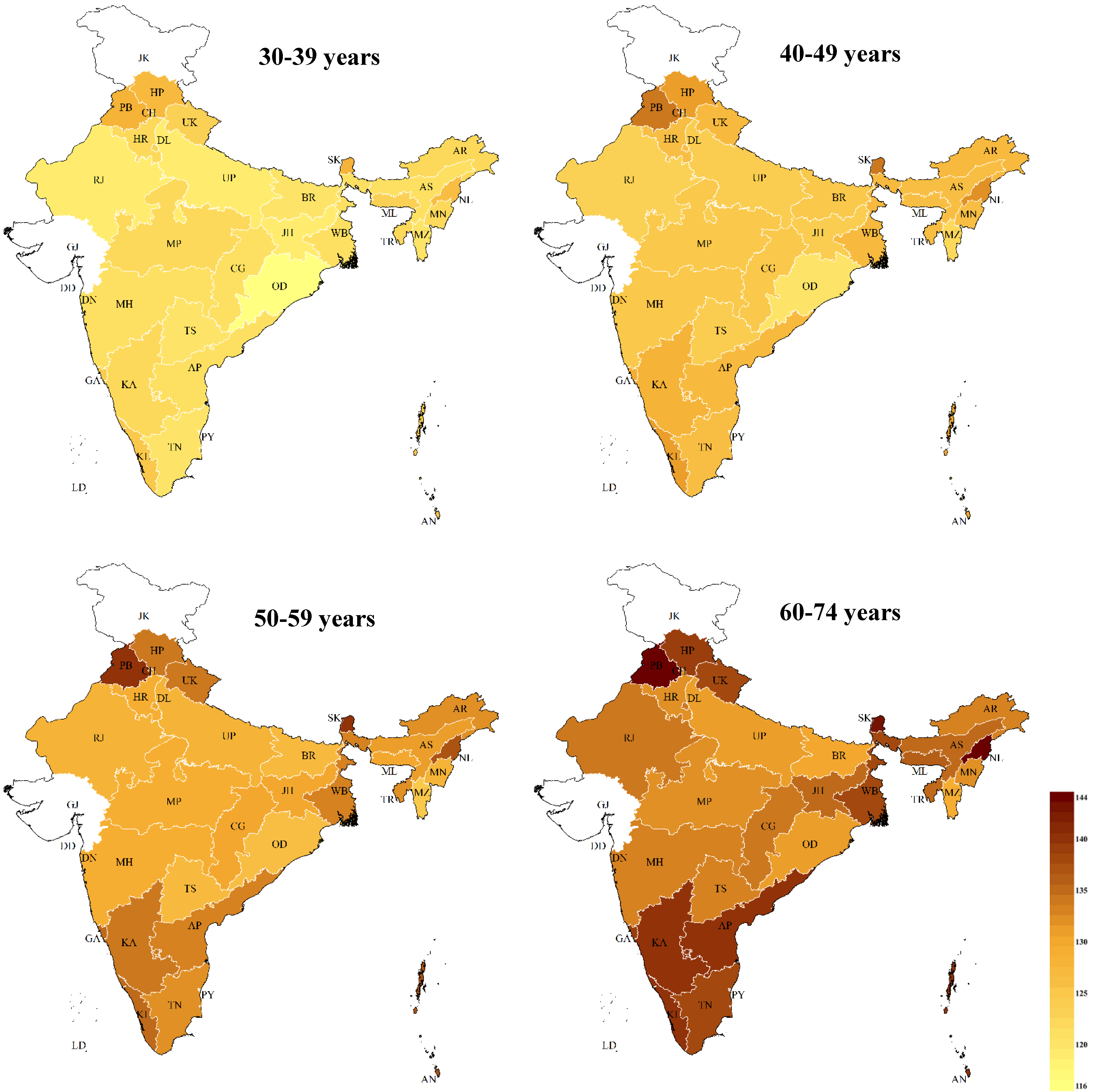
^

^2^ The Global Burden of Disease Project’s 2013 population for India was used for age standardization.[1]

^3^ No data was available for Gujarat, and Jammu and Kashmir.

Abbreviations: AP indicates Andhra Pradesh; AR, Arunachal Pradesh; AS, Assam; BR, Bihar; CG, Chhattisgarh; CH, Chandigarh; DD, Daman and Diu; DL, Delhi; GA, Goa; GJ, Gujarat; HR, Haryana; HP, Himachal Pradesh; JH, Jharkhand; JK, Jammu and Kashmir; KA, Karnataka; KL, Kerala; MP, Madhya Pradesh; MH, Maharashtra; MN, Manipur; ML, Meghalaya; MZ, Mizoram; NL, Nagaland; OD, Odisha (Orissa); PB, Punjab; PY, Puducherry; RJ, Rajasthan; SK, Sikkim; TN, Tamil Nadu; TS, Telangana State; TR, Tripura; UP, Uttar Pradesh; UK, Uttarakhand (Uttaranchal); WB, West Bengal.

Age-standardized state-level mean systolic blood pressure, by rural versus urban areas^2,3^

^
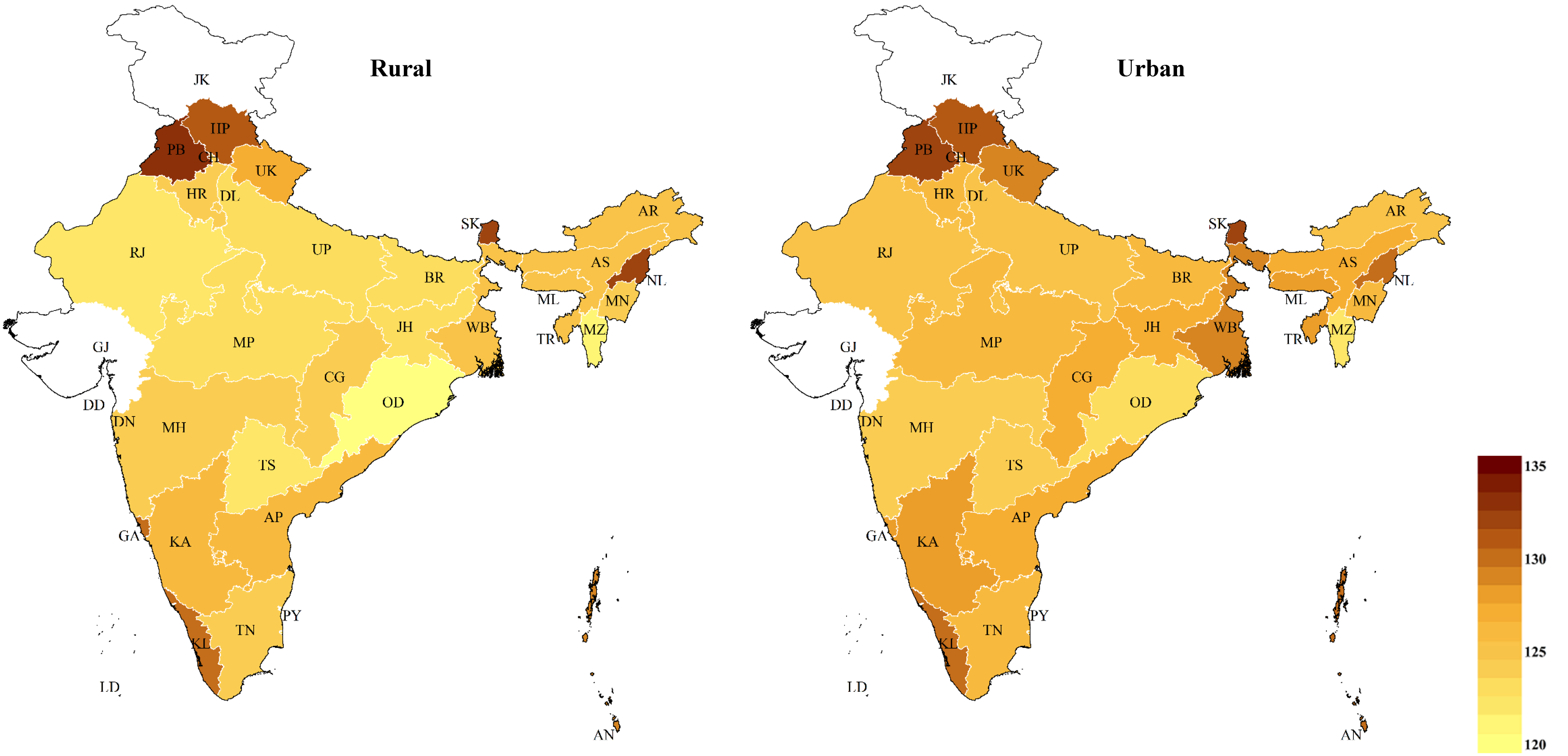
^

^2^ The Global Burden of Disease Project’s 2013 population for India was used for age standardization.[1]

^3^ No data was available for Gujarat, and Jammu and Kashmir.

Abbreviations: AP indicates Andhra Pradesh; AR, Arunachal Pradesh; AS, Assam; BR, Bihar; CG, Chhattisgarh; CH, Chandigarh; DD, Daman and Diu; DL, Delhi; GA, Goa; GJ, Gujarat; HR, Haryana; HP, Himachal Pradesh; JH, Jharkhand; JK, Jammu and Kashmir; KA, Karnataka; KL, Kerala; MP, Madhya Pradesh; MH, Maharashtra; MN, Manipur; ML, Meghalaya; MZ, Mizoram; NL, Nagaland; OD, Odisha (Orissa); PB, Punjab; PY, Puducherry; RJ, Rajasthan; SK, Sikkim; TN, Tamil Nadu; TS, Telangana State; TR, Tripura; UP, Uttar Pradesh; UK, Uttarakhand (Uttaranchal); WB, West Bengal.

Age-standardized state-level mean diastolic blood pressure, by sex^2,3^


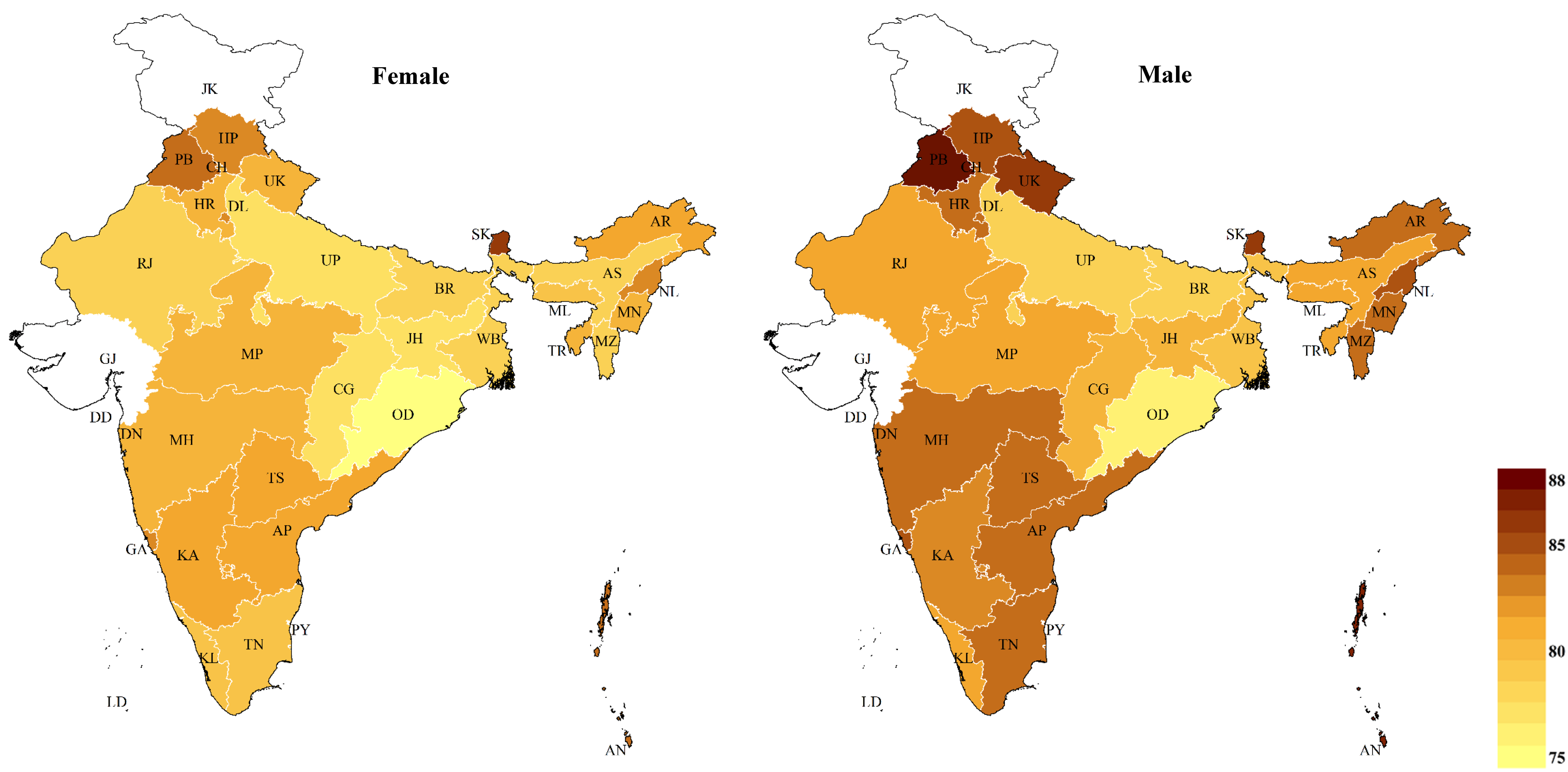


^2^ The Global Burden of Disease Project’s 2013 population for India was used for age standardization.[1]

^3^ No data was available for Gujarat, and Jammu and Kashmir.

Abbreviations: AP indicates Andhra Pradesh; AR, Arunachal Pradesh; AS, Assam; BR, Bihar; CG, Chhattisgarh; CH, Chandigarh; DD, Daman and Diu; DL, Delhi; GA, Goa; GJ, Gujarat; HR, Haryana; HP, Himachal Pradesh; JH, Jharkhand; JK, Jammu and Kashmir; KA, Karnataka; KL, Kerala; MP, Madhya Pradesh; MH, Maharashtra; MN, Manipur; ML, Meghalaya; MZ, Mizoram; NL, Nagaland; OD, Odisha (Orissa); PB, Punjab; PY, Puducherry; RJ, Rajasthan; SK, Sikkim; TN, Tamil Nadu; TS, Telangana State; TR, Tripura; UP, Uttar Pradesh; UK, Uttarakhand (Uttaranchal); WB, West Bengal.

Age-standardized state-level mean diastolic blood pressure, by age group^2,3^

^
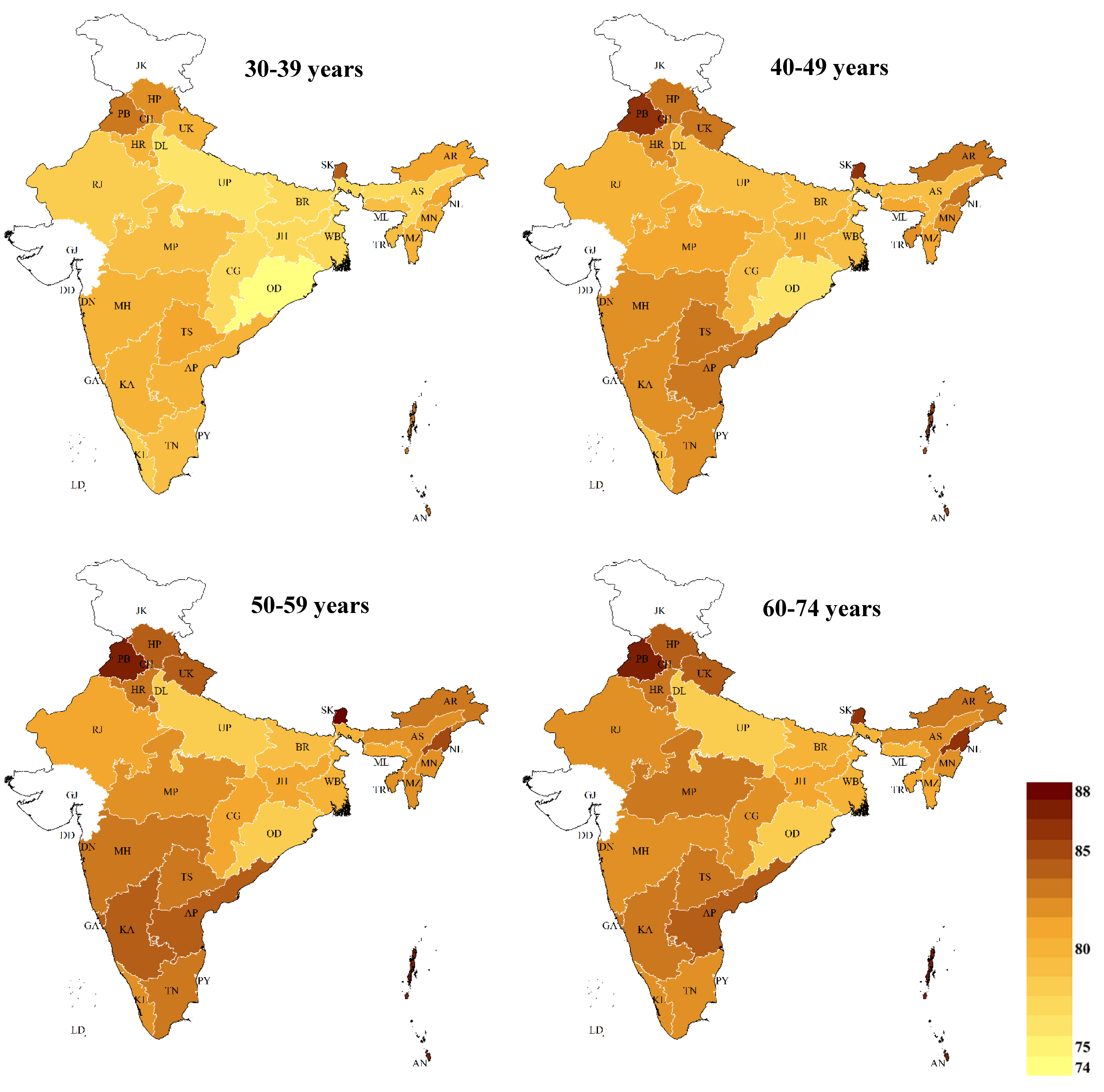
^

^2^ The Global Burden of Disease Project’s 2013 population for India was used for age standardization.[1]

^3^ No data was available for Gujarat, and Jammu and Kashmir.

Abbreviations: AP indicates Andhra Pradesh; AR, Arunachal Pradesh; AS, Assam; BR, Bihar; CG, Chhattisgarh; CH, Chandigarh; DD, Daman and Diu; DL, Delhi; GA, Goa; GJ, Gujarat; HR, Haryana; HP, Himachal Pradesh; JH, Jharkhand; JK, Jammu and Kashmir; KA, Karnataka; KL, Kerala; MP, Madhya Pradesh; MH, Maharashtra; MN, Manipur; ML, Meghalaya; MZ, Mizoram; NL, Nagaland; OD, Odisha (Orissa); PB, Punjab; PY, Puducherry; RJ, Rajasthan; SK, Sikkim; TN, Tamil Nadu; TS, Telangana State; TR, Tripura; UP, Uttar Pradesh; UK, Uttarakhand (Uttaranchal); WB, West Bengal.

Age-standardized state-level mean diastolic blood pressure, by rural versus urban areas^2,3^

^
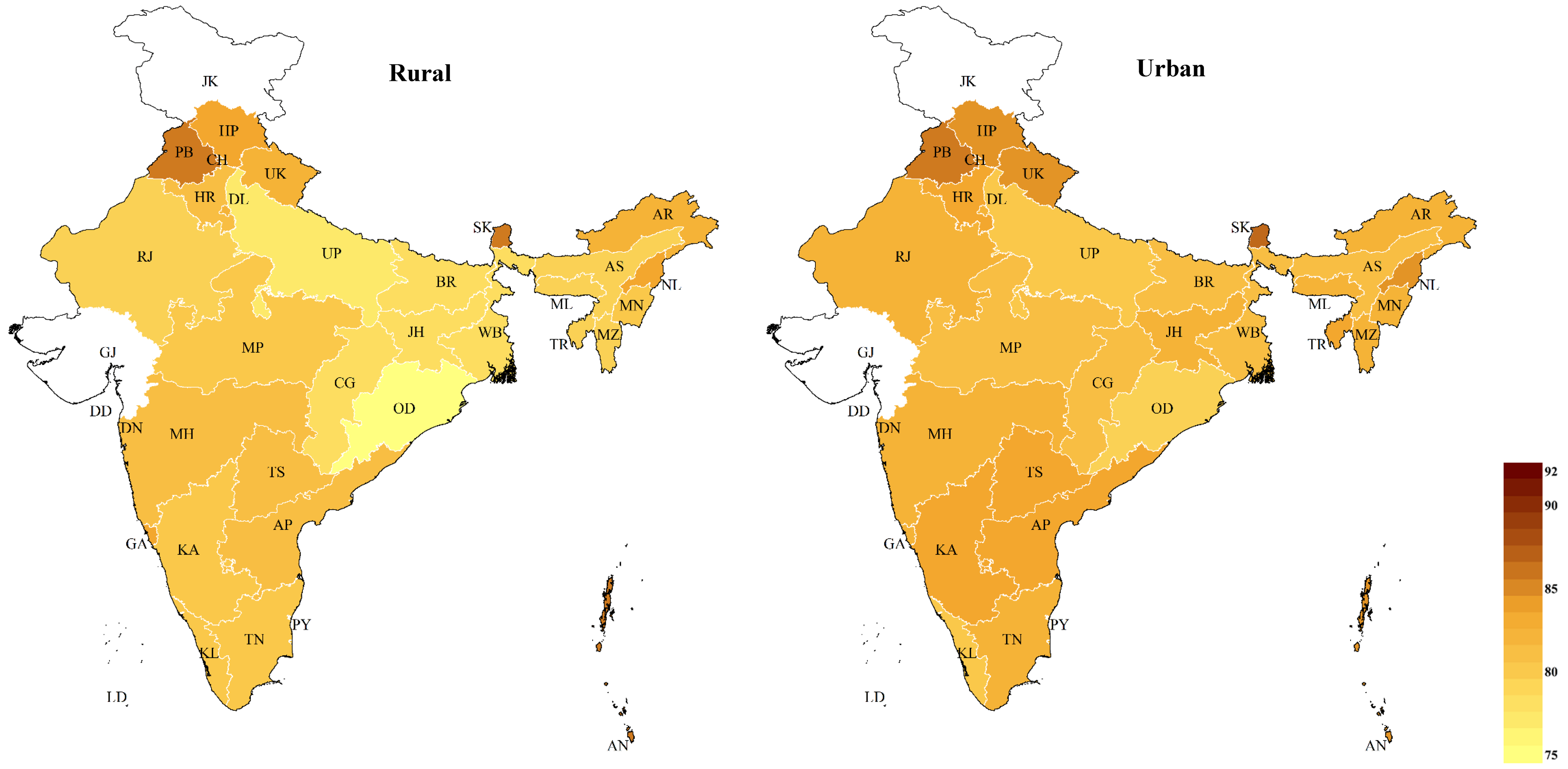
^

^2^ The Global Burden of Disease Project’s 2013 population for India was used for age standardization.[1]

^3^ No data was available for Gujarat, and Jammu and Kashmir.

Abbreviations: AP indicates Andhra Pradesh; AR, Arunachal Pradesh; AS, Assam; BR, Bihar; CG, Chhattisgarh; CH, Chandigarh; DD, Daman and Diu; DL, Delhi; GA, Goa; GJ, Gujarat; HR, Haryana; HP, Himachal Pradesh; JH, Jharkhand; JK, Jammu and Kashmir; KA, Karnataka; KL, Kerala; MP, Madhya Pradesh; MH, Maharashtra; MN, Manipur; ML, Meghalaya; MZ, Mizoram; NL, Nagaland; OD, Odisha (Orissa); PB, Punjab; PY, Puducherry; RJ, Rajasthan; SK, Sikkim; TN, Tamil Nadu; TS, Telangana State; TR, Tripura; UP, Uttar Pradesh; UK, Uttarakhand (Uttaranchal); WB, West Bengal.

**Figure E**. Crude mean 10-year Harvard-NHANES and Globorisk score by household wealth quintile, age group, rural versus urban location, and sex^6^

Harvard-NHANES:

^
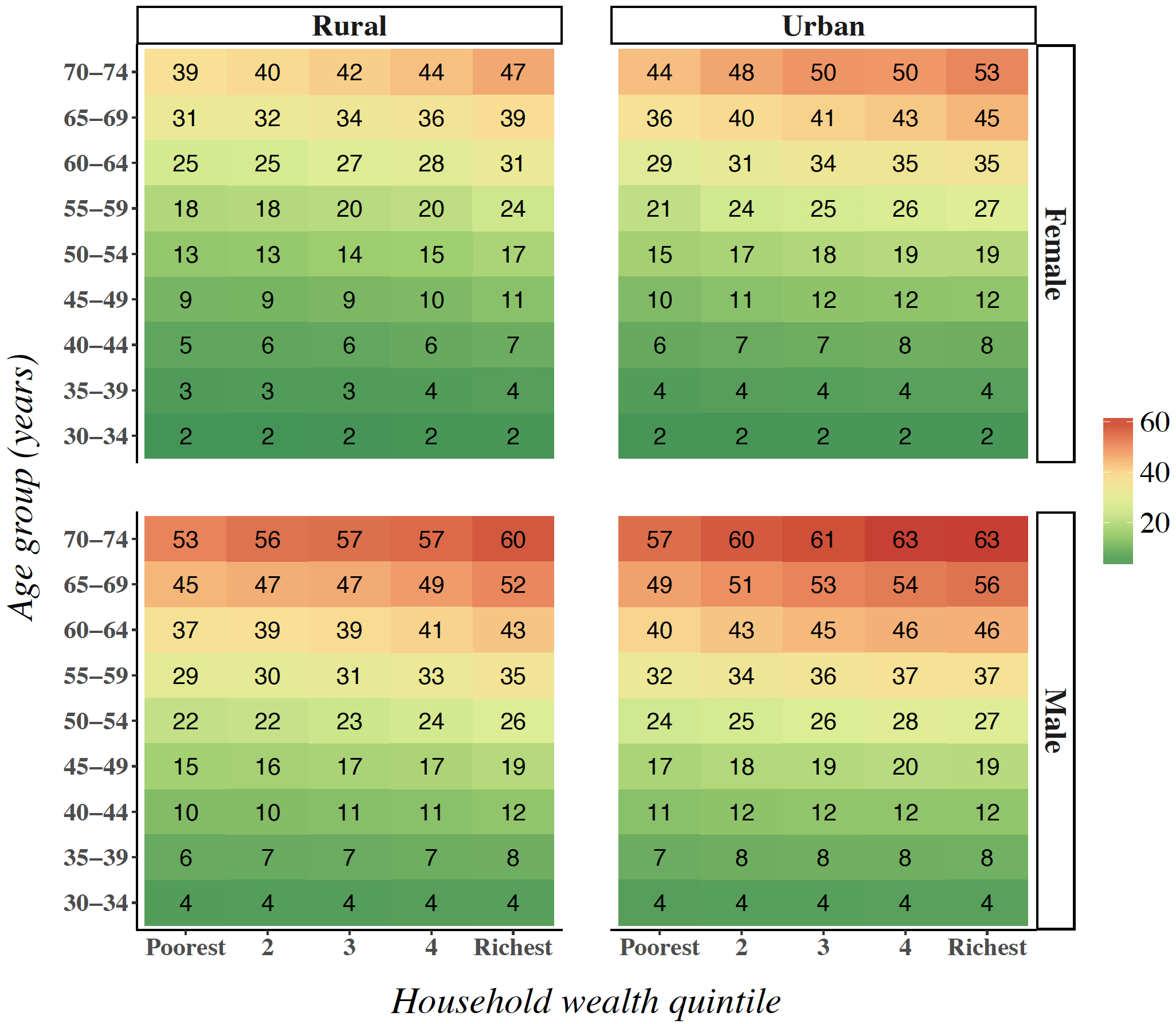
^

^6^ This is the crude (i.e., age-unstandardized) mean 10-year risk (in percent) of a CVD event as calculated by the Harvard-NHANES score.

Globorisk^7,8^:

^
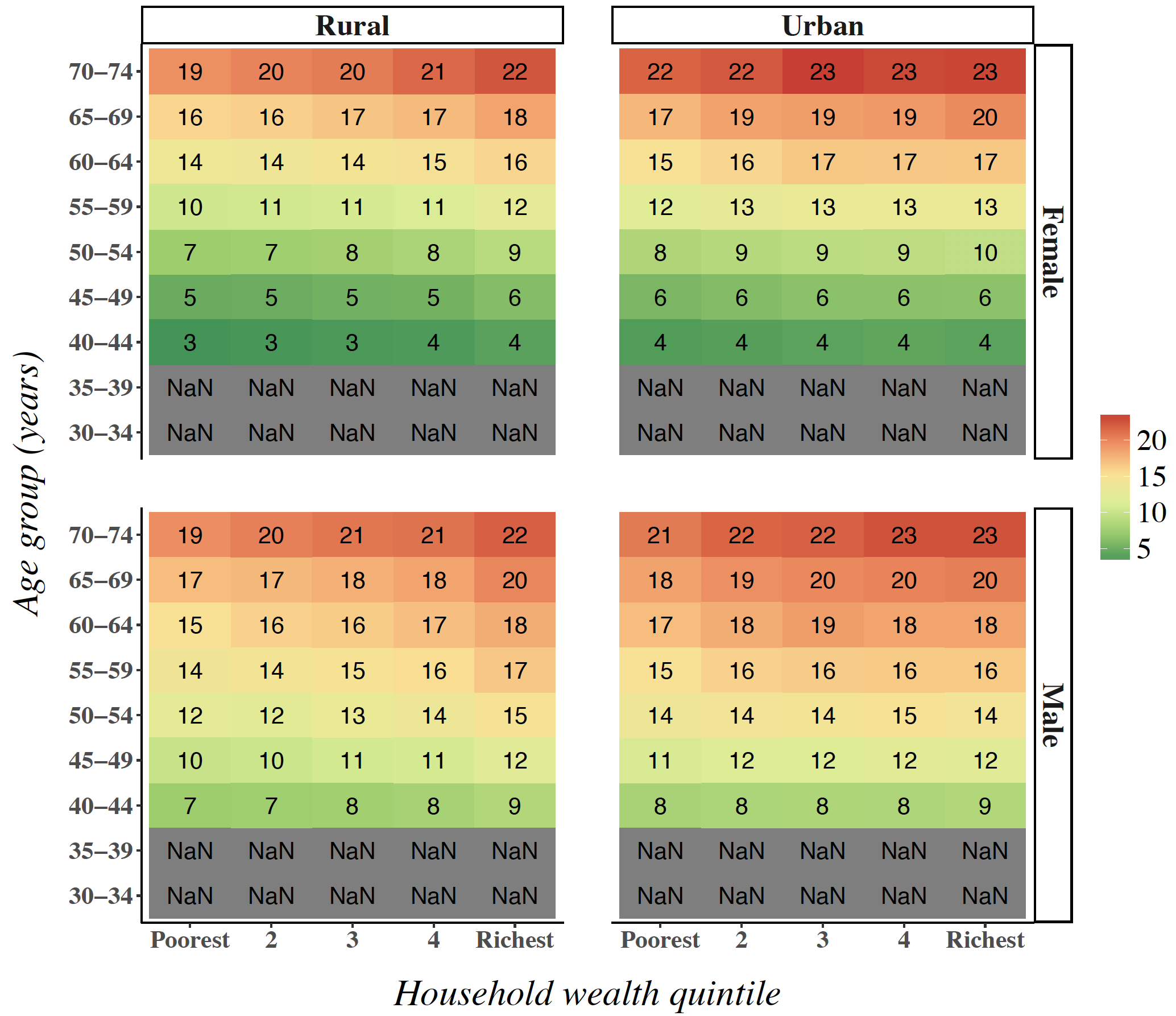
^

7 This is the crude (i.e., age-unstandardized) mean 10-year risk (in percent) of a CVD event as calculated by the Globorisk score.

^8^ Globorisk estimates cardiovascular risk only for those aged 40 to 74 years.

**Figure F. Percentage of population with a high (≥30%) 10-year risk score by household wealth quintile, age group, rural versus urban location, and sex**

Framingham risk score:^9^

^9^ This is the crude prevalence (disaggregated by household wealth quintile, age group, rural-urban residence, and sex) of a high (≥30%) 10-year CVD risk as computed with the Framingham risk score.

Harvard-NHANES:^10^

^10^ This is the crude prevalence (disaggregated by household wealth quintile, age group, rural-urban residence, and sex) of a high (≥30%) 10-year CVD risk as computed with the Harvard-NHANES score.

Globorisk^8,11^:

^8^ Globorisk estimates cardiovascular risk only for those aged 40 to 74 years.

^11^ This is the crude prevalence (disaggregated by household wealth quintile, age group, rural-urban residence, and sex) of a high (≥30%) 10-year CVD risk as computed with the Globorisk score.

WHO-ISH:^12^


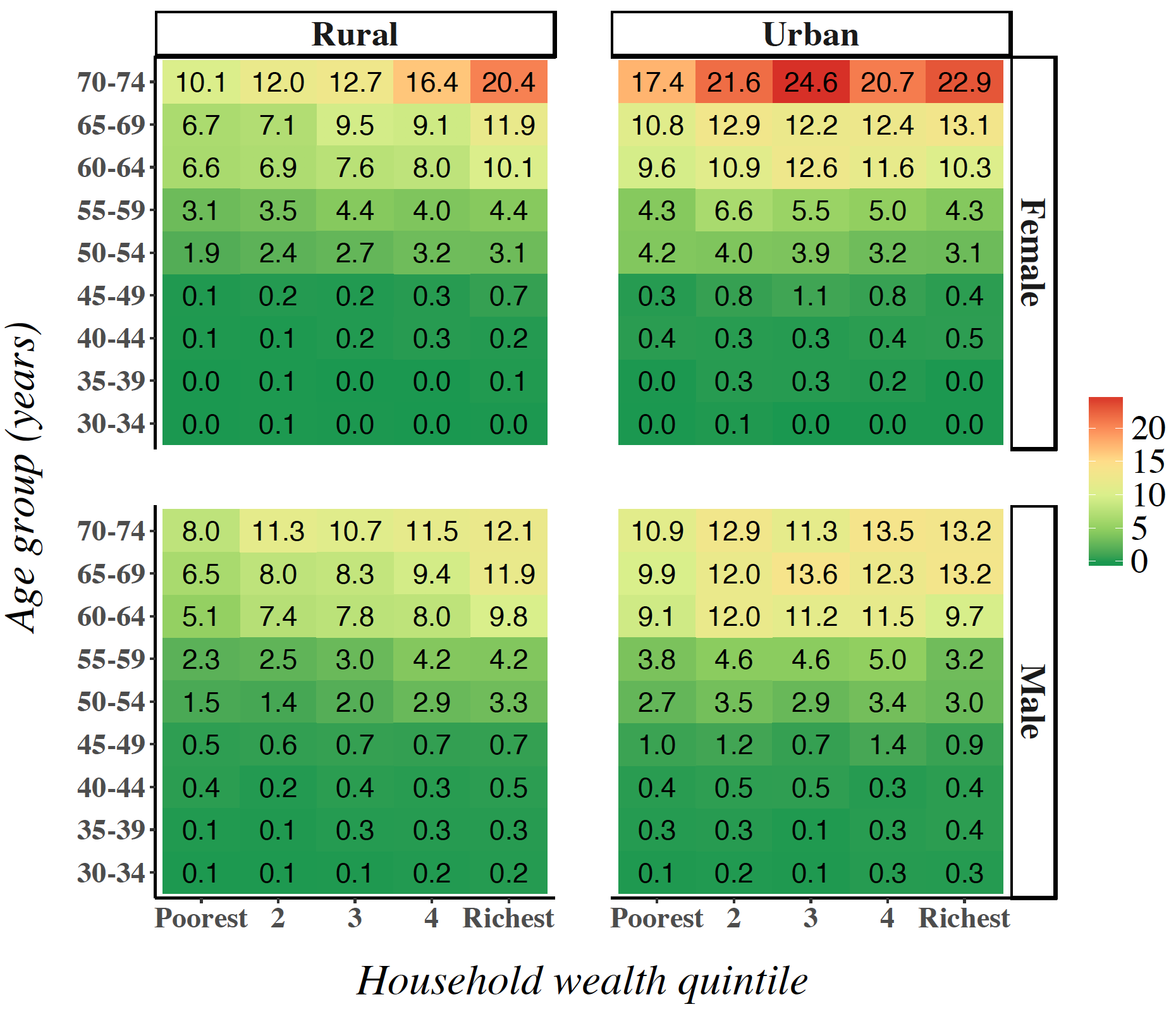


^12^ This is the crude prevalence (disaggregated by household wealth quintile, age group, rural-urban residence, and sex) of a high (≥30%) 10-year CVD risk as computed with the WHO-ISH score.

# **Figure G. Mean diastolic blood pressure by household wealth quintile, age group, rural versus urban location, and sex.**


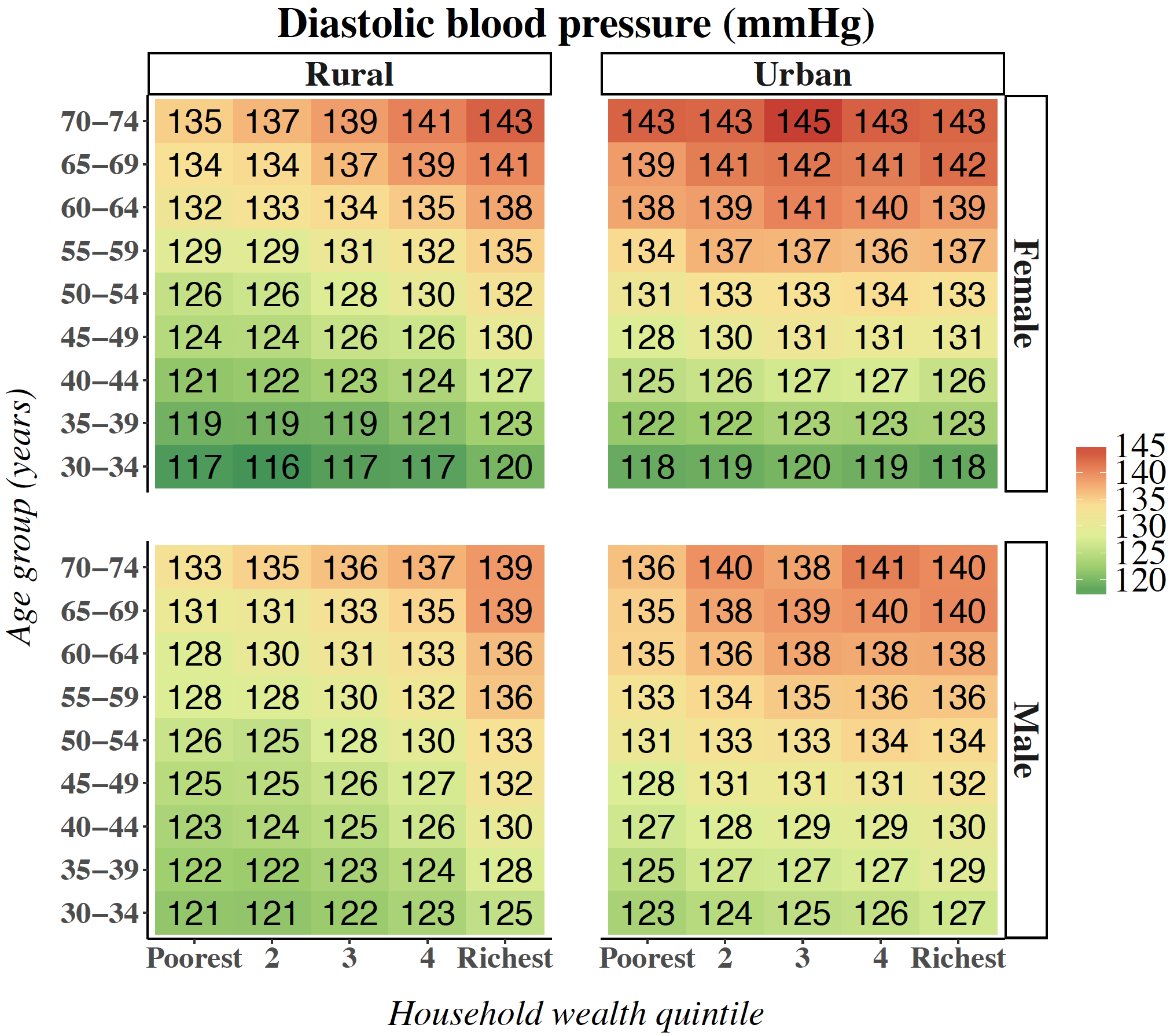


# **References:**

1. Global Burden of Disease Study 2015. Global Burden of Disease Study 2015 (GBD 2015) Population Estimates 1970-2015 Seattle, United States: Institute for Health Metrics and Evaluation (IHME),; 2016 [cited 2017 Jul 17]. Available from: <http://ghdx.healthdata.org/record/global-burden-disease-study-2015-gbd-2015-population-estimates-1970-2015>.
