# Supplementary material for: Geographic and sociodemographic variation of cardiovascular disease risk in India: A cross-sectional study of 797,540 adults
Source: PLoS Med. 2018 Jun 19;15(6):e1002581. doi: 10.1371/journal.pmed.1002581 (PMC6007838; doi:10.1371/journal.pmed.1002581)
Supplement: S1 Table — (DOCX) [file pmed.1002581.s003.docx]

**Supplementary tables**

Table A. Characteristics of adults excluded from the analysis 3

Table B. 10-year cardiovascular risk as calculated by the Framingham risk score, by age group (not age-standardized) 5

Table C. 10-year cardiovascular risk as calculated by Harvard-NHANES, Globorisk, and WHO-ISH by age group (not age-standardized) 6

Table D. Proportion of participants who were overweight, current smokers, hypertensive, or had a high blood glucose 8

Table E. Prevalence of a high (≥30%) 10-year cardiovascular risk, by state and sex 9

Table F. Prevalence of cardiovascular risk factors, by state and sex 12

Table G. Multivariable linear regression of the natural logarithm of 10-year CVD risk (as calculated with the Harvard-NHANES score) on district-level wealth quintile and individual-level socio-demographic characteristics 14

Table H. Multivariable linear regression of the natural logarithm of 10-year CVD risk (as calculated with the Harvard-NHANES score) on the proportion of participants in a district who live in an urban area 15

Table I. Multivariable linear regression of the natural logarithm of 10-year CVD risk (as calculated with Globorisk) on district-level wealth quintile and individual-level socio-demographic characteristics 16

Table J. Multivariable linear regression of the natural logarithm of 10-year CVD risk (as calculated with Globorisk) on the proportion of participants in a district who live in an urban area 17

Table K. Ordinary least squares regressions of the natural logarithm of the 10-year Harvard-NHANES risk score on sociodemographic covariates and district-level fixed effects 18

Table L. Ordinary least squares regressions of the natural logarithm of the 10-year Globorisk score on sociodemographic covariates and district-level fixed effects 19

**Combined legend for all tables in this file.**

^1^ Defined as a 10-year cardiovascular risk ≥30% as computed with the Framingham risk score.

^2^ WHO-ISH computes a risk category rather than a continuous risk score. It was, therefore, not possible to estimate mean risk for WHO-ISH.

^3^ The model included random intercepts by district.

^4^ This model included all variables listed in the table, five-year age groups, and a binary indicator for sex as explanatory variables.

^5^ 10-year cardiovascular disease risk was calculated using the Harvard-NHANES score.

^6^ The dataset was first divided into participants living in rural versus urban areas before district wealth quintile was calculated and the regression model was fitted.

^7^ District wealth quintile was calculated, separately for rural and urban areas within districts, by computing the median of the continuous household wealth index in a district and then categorizing the district-level median into quintiles.

^8^ Coefficients were multiplied by 100 so that they can be interpreted as an approximation of the percentage change in cardiovascular risk associated with a one unit change in the explanatory variable.

^9^ ‘District-level proportion living in an urban area’ refers to the proportion (between 0 and 1) of the participants in a district who live in an urban area.

^10^ Standard errors were adjusted for clustering at the level of a primary sampling unit.

^11^ These models included one sociodemographic characteristic, age group, and a binary indicator variable for each district as explanatory variables.

^12^ This model included all variables listed in the table, age group, and a binary indicator for each district as explanatory variables.

# **Table A. Characteristics of adults excluded from the analysis**

In total, 27.1% (296,822/1,094,754) of participants were excluded from the analysis because they had a missing value for at least one of the cardiovascular disease risk factors needed to compute a predicted cardiovascular risk (age, sex, Body Mass Index, blood glucose, smoking status, systolic blood pressure, and treatment for hypertension). The table below compares (by sex) the sampling characteristics of those who were excluded from the analysis with those who were included.

| **Characteristic** | **Female** | | **Male** | |
| --- | --- | --- | --- | --- |
|  | *Included* | *Excluded* | *Included* | *Excluded* |
| n | 420,852 | 108,937 | 377,080 | 187,885 |
|  |  |  |  |  |
| **Cardiovascular risk factors** |  |  |  |  |
|  |  |  |  |  |
| Age group (%) |  |  |  |  |
| 30-34 years | 17.2 | 18.3 | 15.3 | 18.6 |
| 35-39 years | 17.0 | 16.5 | 15.0 | 16.5 |
| 40-44 years | 15.3 | 14.0 | 14.8 | 15.1 |
| 45-49 years | 13.2 | 11.6 | 13.4 | 12.9 |
| 50-54 years | 11.7 | 11.6 | 11.8 | 11.0 |
| 55-59 years | 8.8 | 8.9 | 9.6 | 8.6 |
| 60-64 years | 7.6 | 8.2 | 8.7 | 7.7 |
| 65-69 years | 5.6 | 6.3 | 6.7 | 5.5 |
| 70-74 years | 3.6 | 4.7 | 4.7 | 4.0 |
|  |  |  |  |  |
| Mean BMI in kg/m^2^ (SD) | 22.6 (4.8) | 22.5 (4.8) | 22.3 (4.1) | 22.1 (4.1) |
| BMI (%) |  |  |  |  |
| <18.5 kg/m^2^ | 17.3 | 17.6 | 15.7 | 16.8 |
| 18.5-22.9 kg/m^2^ | 43.6 | 44.6 | 46.9 | 47.1 |
| 23.0-24.9 kg/m^2^ | 15.1 | 15.0 | 17.2 | 17.2 |
| 25.0-29.9 kg/m^2^ | 17.6 | 16.8 | 16.3 | 15.3 |
| ≥30.0 kg/m^2^ | 6.4 | 5.9 | 3.9 | 3.5 |
|  |  |  |  |  |
| Diabetes (%) | 10.0 | 10.0 | 10.8 | 12.9 |
|  |  |  |  |  |
| Current smoking (%) | 2.6 | 2.6 | 27.1 | 27.9 |
|  |  |  |  |  |
| Mean systolic BP in mmHg (SD) | 126.7 (21.3) | 126.4 (21.3) | 129.1 (19.7) | 128.6 (19.5) |
| Systolic BP (%) |  |  |  |  |
| <120 mmHg | 40.1 | 40.1 | 31.4 | 31.6 |
| 120 – 129 mmHg | 22.1 | 22.4 | 24.6 | 24.9 |
| 130 – 139 mmHg | 15.7 | 15.9 | 20.0 | 21.1 |
| 140 – 179 mmHg | 19.4 | 19.2 | 21.9 | 20.6 |
| ≥180 mmHg | 2.6 | 2.4 | 2.1 | 1.8 |
|  |  |  |  |  |
| Current treatment for hypertension | 2.3 | 2.1 | 1.7 | 1.4 |
|  |  |  |  |  |
| **Socio-demographic characteristics** |  |  |  |  |
|  |  |  |  |  |
| Educational attainment (%) |  |  |  |  |
| <Primary School | 56.4 | 57.1 | 34.1 | 30.6 |
| Primary School | 12.1 | 11.2 | 13.6 | 12.3 |
| Middle School | 12.0 | 11.5 | 16.2 | 16.2 |
| Secondary School | 9.6 | 8.9 | 15.8 | 16.9 |
| High School | 4.7 | 4.9 | 8.8 | 9.9 |
| >High School | 5.3 | 6.3 | 11.5 | 14.0 |
|  |  |  |  |  |
| Urban area (%) | 32.4 | 32.6 | 32.2 | 36.8 |
|  |  |  |  |  |
| Wealth quintile (%) |  |  |  |  |
| 1 (poorest) | 21.6 | 17.4 | 21.0 | 15.2 |
| 2 | 19.9 | 18.8 | 19.8 | 17.9 |
| 3 | 19.1 | 20.0 | 19.1 | 20.4 |
| 4 | 19.4 | 21.5 | 19.8 | 22.2 |
| 5 (richest) | 20.0 | 22.3 | 20.2 | 24.3 |

# **Table B. 10-year cardiovascular risk as calculated by the Framingham risk score, by age group (not age-standardized)**

|  | **Mean risk** | | **Percentage at high risk^1^** | |
| --- | --- | --- | --- | --- |
| **Age** | ***Female***  (95% CI) | ***Female***  (95% CI) | ***Female***  (95% CI) | ***Male***  (95% CI) |
| 30-34 years | 3.9 (3.9, 3.9) | 6.1 (6.1, 6.2) | 0.0 (0.0, 0.0) | 0.1 (0.0, 0.1) |
| 35-39 years | 6.3 (6.3, 6.3) | 10.1 (10.0, 10.2) | 0.2 (0.2, 0.2) | 0.8 (0.7, 0.9) |
| 40-44 years | 9.7 (9.6, 9.7) | 15.1 (15.0, 15.2) | 1.5 (1.4, 1.6) | 4.7 (4.4, 5.0) |
| 45-49 years | 14.2 (14.1, 14.3) | 21.6 (21.5, 21.8) | 6.2 (5.9, 6.6) | 18.1 (17.6, 18.7) |
| 50-54 years | 19.5 (19.3, 19.7) | 28.9 (28.7, 29.1) | 14.8 (14.2, 15.3) | 38.0 (37.1, 38.8) |
| 55-59 years | 25.4 (25.1, 25.6) | 37.1 (36.8, 37.4) | 26.9 (26.2, 27.7) | 60.3 (59.4, 61.1) |
| 60-64 years | 32.2 (31.8, 32.5) | 44.8 (44.5, 45.1) | 43.7 (42.8, 44.6) | 80.0 (79.3, 80.7) |
| 65-69 years | 38.0 (37.7, 38.4) | 51.9 (51.5, 52.2) | 59.4 (58.5, 60.4) | 91.5 (90.9, 92.0) |
| 70-74 years | 44.3 (43.8, 44.7) | 58.7 (58.3, 59.1) | 73.1 (72.0, 74.1) | 96.9 (96.6, 97.3) |
|  |  |  |  |  |
| *30-74 years* | *12.7 (12.7, 12.8)* | *21.4 (21.3, 21.6)* | *14.6 (14.4, 14.8)* | *31.7 (31.4, 32.0)* |
| *50-74 years* | *28.7 (28.6, 28.9)* | *41.4 (41.2, 41.6)* | *36.1 (35.6, 36.5)* | *67.6 (67.1, 68.1)* |

Abbreviation: CI=confidence interval.

^1^ Defined as a 10-year cardiovascular risk ≥30% as computed with the Framingham risk score.

# **Table C. 10-year cardiovascular risk as calculated by Harvard-NHANES, Globorisk, and WHO-ISH by age group (not age-standardized)**

|  | **Harvard-NHANES** | | **Globorisk** | | **WHO-ISH^2^** | |
| --- | --- | --- | --- | --- | --- | --- |
|  | ***Female***  (95% CI) | ***Male***  (95% CI) | ***Female***  (95% CI) | ***Male***  (95% CI) | ***Female***  (95% CI) | ***Male***  (95% CI) |
| **Mean risk (%)** |  |  |  |  |  |  |
| 30-34 years | 2.0 (2.0, 2.0) | 4.0 (4.0, 4.0) | - | - | - | - |
| 35-39 years | 3.7 (3.7, 3.7) | 7.1 (7.0, 7.1) | - | - | - | - |
| 40-44 years | 6.3 (6.2, 6.3) | 11.3 (11.2, 11.3) | 3.7 (3.7, 3.7) | 7.9 (7.8, 7.9) | - | - |
| 45-49 years | 10.1 (10.1, 10.2) | 17.1 (17.0, 17.2) | 5.5 (5.5, 5.6) | 11.0 (10.9, 11.1) | - | - |
| 50-54 years | 15.2 (15.1, 15.4) | 24.1 (24.0, 24.3) | 8.2 (8.2, 8.3) | 13.5 (13.3, 13.6) | - | - |
| 55-59 years | 21.2 (21.0, 21.3) | 32.6 (32.3, 32.8) | 11.5 (11.4, 11.6) | 15.3 (15.2, 15.5) | - | - |
| 60-64 years | 28.5 (28.3, 28.8) | 40.9 (40.7, 41.2) | 14.9 (14.8, 15.0) | 17.0 (16.8, 17.1) | - | - |
| 65-69 years | 35.8 (35.5, 36.1) | 49.0 (48.7, 49.4) | 17.2 (17.1, 17.4) | 18.4 (18.3, 18.5) | - | - |
| 70-74 years | 43.5 (43.2, 43.9) | 57.2 (56.8, 57.5) | 21.1 (20.9, 21.3) | 20.9 (20.7, 21.1) | - | - |
| *30-74 years* | 15.9 (15.8, 16.0) | 25.0 (24.9, 25.1) | - | - | - | - |
| *50-74 years* | 25.3 (25.1, 25.4) | 37.6 (37.4, 37.7) | 13.0 (12.9, 13.1) | 16.3 (16.2, 16.4) | - | - |
| **% at high risk** | | | | | | |
| 30-34 years | 0.0 (0.0, 0.0) | 0.0 (0.0, 0.0) | - | - | 0.0 (0.0, 0.1) | 0.1 (0.1, 0.2) |
| 35-39 years | 0.0 (0.0, 0.0) | 0.1 (0.1, 0.1) | - | - | 0.1 (0.1, 0.1) | 0.2 (0.2, 0.3) |
| 40-44 years | 0.0 (0.0, 0.1) | 1.1 (1.0, 1.2) | 0.0 (0.0, 0.0) | 0.5 (0.4, 0.6) | 0.2 (0.2, 0.3) | 0.4 (0.3, 0.5) |
| 45-49 years | 0.8 (0.7, 0.9) | 6.9 (6.6, 7.3) | 0.1 (0.1, 0.2) | 1.7 (1.5, 1.9) | 0.4 (0.3, 0.5) | 0.8 (0.7, 0.9) |
| 50-54 years | 5.4 (5.0, 5.8) | 23.3 (22.7, 24.0) | 0.7 (0.6, 0.8) | 3.0 (2.8, 3.2) | 3.0 (2.7, 3.2) | 2.4 (2.2, 2.6) |
| 55-59 years | 15.3 (14.8, 15.9) | 47.7 (46.9, 48.6) | 1.9 (1.7, 2.1) | 4.6 (4.2, 4.9) | 4.2 (3.9, 4.5) | 3.5 (3.2, 3.8) |
| 60-64 years | 33.8 (33.0, 34.7) | 73.8 (73.1, 74.5) | 4.4 (4.1, 4.7) | 5.8 (5.4, 6.2) | 8.6 (8.2, 9.1) | 8.4 (8.0, 8.9) |
| 65-69 years | 58.5 (57.5, 59.5) | 91.2 (90.7, 91.8) | 6.9 (6.5, 7.4) | 7.0 (6.6, 7.4) | 9.5 (9.0, 10.0) | 9.5 (9.0, 10.0) |
| 70-74 years | 82.6 (81.7, 83.5) | 98.0 (97.7, 98.2) | 13.2 (12.4, 13.9) | 11.0 (10.3, 11.8) | 15.7 (14.9, 16.5) | 11.0 (10.2, 11.7) |
| *30-74 years* | *11.1 (10.9, 11.2)* | *26.1 (25.9, 26.4)* | *-* | *-* | *2.6 (2.5, 2.7)* | *2.8 (2.7, 2.9)* |
| *50-74 years* | *29.2 (28.8, 29.6)* | *59.4 (58.9, 59.9)* | *3.9 (3.7, 4.0)* | *5.5 (5.3, 5.7)* | *6.7 (6.5, 6.8)* | *6.1 (5.9, 6.3)* |

Abbreviation: CI=confidence interval.

^2^ WHO-ISH computes a risk category rather than a continuous risk score. It was, therefore, not possible to estimate mean risk for WHO-ISH.

# **Table D. Proportion of participants who were overweight, current smokers, hypertensive, or had a high blood glucose**

The table below shows the proportion of participants who were either current smokers, had a high blood glucose (or reporting to be on regular treatment for diabetes), hypertension, or who were overweight. Diabetes was defined as having a high blood glucose reading or reporting to be on regular treatment for diabetes. Hypertension was defined as a systolic blood pressure ≥140mmHg, a diastolic blood pressure ≥90mmHg, or reporting to be on regular treatment for hypertension. Overweight was defined as a Body Mass Index ≥25kg/m^2^. The proportions shown were weighted using sampling weights (but not age-standardized).

| **Age** | ***Female***  (95% CI) | ***Male***  (95% CI) |
| --- | --- | --- |
| 30-34 years | 32.26 (31.60 - 32.93) | 49.31 (48.58 - 50.04) |
| 35-39 years | 38.22 (37.56 - 38.88) | 55.79 (55.06 - 56.53) |
| 40-44 years | 43.66 (42.95 - 44.38) | 59.74 (58.97 - 60.52) |
| 45-49 years | 49.15 (48.43 - 49.87) | 63.73 (62.99 - 64.47) |
| 50-54 years | 54.14 (53.32 - 54.96) | 66.15(65.29 – 67.00) |
| 55-59 years | 56.00 (55.13 - 56.87) | 68.37 (67.57 - 69.18) |
| 60-64 years | 60.90 (59.98 - 61.81) | 70.04 (69.26 - 70.82) |
| 65-69 years | 61.48 (60.48 - 62.49) | 69.63 (68.72 - 70.53) |
| 70-74 years | 63.39 (62.20 - 64.56) | 70.00 (68.99 - 71.01) |
|  |  |  |
| *30-74 years* | *53.0 (52.5 - 53.5)* | *67.2 (66.8 - 67.7)* |
| *50-74 years* | *46.84 (46.36 - 47.33)* | *61.82 (61.36 - 62.27)* |

# **Table E. Prevalence of a high (≥30%) 10-year cardiovascular risk, by state and sex**^3^

| ***State*** | ***Sex*** | **Framingham** | | | **Harvard-NHANES** | | | **Globorisk** | | |
| --- | --- | --- | --- | --- | --- | --- | --- | --- | --- | --- |
|  |  | ***Estimate*** | ***Lower CI*** | ***Upper CI*** | ***Estimate*** | ***Lower CI*** | ***Upper CI*** | ***Estimate*** | ***Lower CI*** | ***Upper CI*** |
| Andaman and Nicobar | Female | 12.4 | 10.6 | 14.4 | 5.8 | 4.7 | 6.9 | 3.4 | 2.3 | 4.7 |
| Andaman and Nicobar | Male | 22.8 | 20.7 | 25.0 | 16.6 | 14.6 | 18.7 | 5.0 | 3.6 | 6.7 |
| Andhra Pradesh | Female | 12.7 | 12.1 | 13.3 | 8.5 | 8.0 | 8.9 | 2.4 | 2.1 | 2.7 |
| Andhra Pradesh | Male | 24.2 | 23.3 | 25.1 | 18.4 | 17.7 | 19.1 | 5.3 | 4.8 | 5.8 |
| Arunachal Pradesh | Female | 6.4 | 5.7 | 7.1 | 3.5 | 2.8 | 4.2 | 1.9 | 1.2 | 2.7 |
| Arunachal Pradesh | Male | 20.1 | 18.9 | 21.4 | 13.1 | 12.1 | 14.2 | 2.7 | 2.2 | 3.2 |
| Assam | Female | 5.0 | 4.5 | 5.6 | 3.1 | 2.8 | 3.5 | 0.4 | 0.3 | 0.5 |
| Assam | Male | 17.2 | 15.9 | 18.6 | 12.5 | 11.5 | 13.5 | 1.5 | 1.3 | 1.9 |
| Bihar | Female | 5.2 | 4.7 | 5.6 | 3.3 | 3.0 | 3.5 | 0.6 | 0.5 | 0.7 |
| Bihar | Male | 16.6 | 15.6 | 17.6 | 12.6 | 11.8 | 13.4 | 1.3 | 1.0 | 1.5 |
| Chandigarh | Female | 9.9 | 8.3 | 11.5 | 5.8 | 4.8 | 6.9 | 0.7 | 0.4 | 1.2 |
| Chandigarh | Male | 21.7 | 18.8 | 24.8 | 14.9 | 12.5 | 17.4 | 2.6 | 1.7 | 3.8 |
| Chhattisgarh | Female | 5.5 | 4.7 | 6.3 | 3.4 | 2.9 | 4.0 | 1.2 | 0.8 | 1.7 |
| Chhattisgarh | Male | 14.4 | 13.2 | 15.6 | 10.5 | 9.5 | 11.7 | 1.4 | 1.0 | 1.9 |
| Daman and Diu | Female | 10.6 | 8.4 | 12.9 | 6.1 | 4.4 | 8.1 | 1.7 | 0.8 | 2.9 |
| Daman and Diu | Male | 17.8 | 12.9 | 23.4 | 11.3 | 7.9 | 15.4 | 3.7 | 1.3 | 7.3 |
| Goa | Female | 15.5 | 13.7 | 17.3 | 10.6 | 9.3 | 11.9 | 1.8 | 1.2 | 2.4 |
| Goa | Male | 25.8 | 23.2 | 28.5 | 19.9 | 17.6 | 22.3 | 2.9 | 1.7 | 4.5 |
| Haryana | Female | 8.3 | 8.0 | 8.7 | 6.1 | 5.8 | 6.4 | 1.3 | 1.1 | 1.5 |
| Haryana | Male | 21.4 | 20.8 | 22.0 | 15.7 | 15.2 | 16.2 | 2.8 | 2.5 | 3.1 |
| Himachal Pradesh | Female | 9.2 | 8.4 | 10.0 | 5.7 | 5.1 | 6.2 | 1.9 | 1.6 | 2.3 |
| Himachal Pradesh | Male | 29.3 | 27.7 | 30.9 | 21.0 | 19.7 | 22.3 | 4.8 | 4.0 | 5.7 |
| Jharkhand | Female | 6.2 | 5.5 | 6.9 | 3.5 | 3.1 | 4.0 | 0.8 | 0.6 | 1.0 |
| Jharkhand | Male | 15.4 | 14.2 | 16.6 | 11.7 | 10.7 | 12.7 | 2.1 | 1.6 | 2.6 |
| Karnataka | Female | 11.8 | 11.4 | 12.1 | 7.4 | 7.1 | 7.6 | 2.0 | 1.9 | 2.2 |
| Karnataka | Male | 20.8 | 20.4 | 21.3 | 15.7 | 15.3 | 16.1 | 4.1 | 3.8 | 4.4 |
| Kerala | Female | 14.3 | 13.6 | 14.9 | 9.9 | 9.4 | 10.5 | 2.1 | 1.9 | 2.5 |
| Kerala | Male | 30.4 | 28.8 | 32.0 | 22.4 | 21.2 | 23.6 | 5.1 | 4.4 | 5.8 |
| Madhya Pradesh | Female | 5.3 | 4.6 | 6.2 | 3.6 | 3.1 | 4.1 | 0.5 | 0.3 | 0.6 |
| Madhya Pradesh | Male | 15.7 | 15.0 | 16.5 | 11.1 | 10.6 | 11.7 | 1.1 | 0.8 | 1.4 |
| Maharashtra | Female | 8.2 | 7.9 | 8.5 | 5.8 | 5.5 | 6.0 | 1.2 | 1.1 | 1.3 |
| Maharashtra | Male | 16.2 | 15.8 | 16.6 | 12.5 | 12.2 | 12.9 | 2.1 | 1.9 | 2.3 |
| Manipur | Female | 9.1 | 8.5 | 9.8 | 6.3 | 5.8 | 6.8 | 1.7 | 1.4 | 2.1 |
| Manipur | Male | 28.4 | 27.3 | 29.6 | 19.5 | 18.6 | 20.4 | 3.1 | 2.7 | 3.7 |
| Meghalaya | Female | 6.3 | 5.5 | 7.1 | 3.4 | 2.9 | 4.0 | 1.4 | 1.0 | 1.8 |
| Meghalaya | Male | 21.7 | 20.1 | 23.4 | 14.4 | 13.1 | 15.8 | 5.1 | 4.0 | 6.2 |
| Mizoram | Female | 6.5 | 6.0 | 7.1 | 4.4 | 4.1 | 4.8 | 2.2 | 1.7 | 2.6 |
| Mizoram | Male | 23.1 | 22.1 | 24.0 | 15.0 | 14.2 | 15.7 | 3.2 | 2.7 | 3.7 |
| Nagaland | Female | 10.7 | 9.7 | 11.7 | 5.5 | 4.9 | 6.1 | 2.8 | 2.2 | 3.3 |
| Nagaland | Male | 30.2 | 28.5 | 31.9 | 21.4 | 20.0 | 22.9 | 6.9 | 6.0 | 7.8 |
| NCT of Delhi | Female | 8.5 | 7.7 | 9.3 | 4.7 | 4.2 | 5.3 | 0.9 | 0.6 | 1.3 |
| NCT of Delhi | Male | 17.4 | 16.4 | 18.6 | 12.1 | 11.3 | 13.0 | 2.3 | 1.8 | 2.8 |
| Odisha | Female | 6.3 | 5.9 | 6.7 | 4.0 | 3.7 | 4.3 | 0.7 | 0.6 | 0.8 |
| Odisha | Male | 16.9 | 16.1 | 17.7 | 13.1 | 12.5 | 13.7 | 1.8 | 1.6 | 2.1 |
| Puducherry | Female | 14.5 | 13.6 | 15.5 | 9.9 | 9.1 | 10.7 | 1.9 | 1.5 | 2.4 |
| Puducherry | Male | 22.1 | 20.5 | 23.8 | 18.1 | 16.7 | 19.5 | 3.8 | 3.0 | 4.6 |
| Punjab | Female | 12.9 | 12.5 | 13.3 | 8.6 | 8.3 | 8.9 | 2.5 | 2.3 | 2.7 |
| Punjab | Male | 22.7 | 22.2 | 23.3 | 16.9 | 16.4 | 17.3 | 4.8 | 4.4 | 5.1 |
| Rajasthan | Female | 6.3 | 5.8 | 6.8 | 4.4 | 4.0 | 4.8 | 1.1 | 0.9 | 1.2 |
| Rajasthan | Male | 17.9 | 16.8 | 19.1 | 13.0 | 12.2 | 13.9 | 1.8 | 1.5 | 2.2 |
| Sikkim | Female | 12.3 | 11.2 | 13.4 | 6.6 | 5.9 | 7.4 | 4.6 | 3.7 | 5.6 |
| Sikkim | Male | 20.0 | 18.7 | 21.4 | 13.9 | 12.7 | 15.1 | 6.5 | 5.2 | 7.8 |
| Tamil Nadu | Female | 13.3 | 12.9 | 13.7 | 8.4 | 8.1 | 8.7 | 1.8 | 1.6 | 1.9 |
| Tamil Nadu | Male | 22.7 | 22.2 | 23.2 | 17.7 | 17.3 | 18.1 | 3.8 | 3.5 | 4.0 |
| Telangana | Female | 8.4 | 7.8 | 9.1 | 5.6 | 5.1 | 6.1 | 1.2 | 0.9 | 1.5 |
| Telangana | Male | 20.3 | 19.4 | 21.3 | 15.1 | 14.3 | 15.9 | 3.4 | 2.9 | 3.9 |
| Tripura | Female | 9.2 | 7.9 | 10.5 | 5.1 | 4.3 | 5.9 | 1.7 | 1.0 | 2.4 |
| Tripura | Male | 21.4 | 19.3 | 23.5 | 15.2 | 13.6 | 16.9 | 3.0 | 2.2 | 3.9 |
| Uttar Pradesh | Female | 7.2 | 6.7 | 7.7 | 4.6 | 4.3 | 5.0 | 1.1 | 0.9 | 1.3 |
| Uttar Pradesh | Male | 19.9 | 19.0 | 20.8 | 14.4 | 13.8 | 15.1 | 2.2 | 1.8 | 2.6 |
| Uttarakhand | Female | 10.2 | 8.9 | 11.6 | 7.7 | 6.7 | 8.8 | 1.7 | 1.3 | 2.3 |
| Uttarakhand | Male | 25.9 | 23.3 | 28.5 | 20.6 | 18.4 | 23.0 | 4.3 | 3.4 | 5.4 |
| West Bengal | Female | 10.1 | 9.6 | 10.6 | 5.7 | 5.3 | 6.0 | 1.8 | 1.5 | 2.0 |
| West Bengal | Male | 25.2 | 24.4 | 26.1 | 18.7 | 18.0 | 19.4 | 4.2 | 3.8 | 4.6 |

**Abbreviations:** CI=95% confidence interval

^3^ Harvard-NHANES and Framingham estimates are for those aged 30 to 74 years, while Globorisk estimates are for those aged 40 to 74 years.

# **Table F. Prevalence of cardiovascular risk factors, by state and sex**

| **State** | **Sex** | **Mean BMI (kg/m^2^)** | | | **Diabetes prevalence (%)** | | | **Mean systolic BP (mmHg)** | | | **Smoking prevalence (%)** | | |
| --- | --- | --- | --- | --- | --- | --- | --- | --- | --- | --- | --- | --- | --- |
|  |  | ***Estimate*** | ***Lower CI*** | ***Upper CI*** | ***Estimate*** | ***Lower CI*** | ***Upper CI*** | ***Estimate*** | ***Lower CI*** | ***Upper CI*** | ***Estimate*** | ***Lower CI*** | ***Upper CI*** |
| Andaman and Nicobar | Female | 23.8 | 23.4 | 24.2 | 11.1 | 9.0 | 13.4 | 128.8 | 126.9 | 130.8 | 2.7 | 1.7 | 3.9 |
| Andaman and Nicobar | Male | 23.7 | 23.4 | 24.1 | 12.1 | 9.9 | 14.4 | 132.0 | 130.3 | 133.7 | 24.6 | 21.1 | 28.3 |
| Andhra Pradesh | Female | 23.5 | 23.4 | 23.7 | 13.0 | 12.2 | 13.9 | 125.5 | 125.0 | 126.0 | 3.4 | 2.7 | 4.2 |
| Andhra Pradesh | Male | 23.5 | 23.4 | 23.7 | 14.3 | 13.4 | 15.2 | 128.8 | 128.3 | 129.3 | 30.2 | 28.6 | 31.9 |
| Arunachal Pradesh | Female | 23.0 | 22.8 | 23.2 | 6.4 | 5.1 | 8.0 | 125.2 | 124.1 | 126.4 | 8.5 | 7.3 | 9.7 |
| Arunachal Pradesh | Male | 22.7 | 22.6 | 22.9 | 6.2 | 4.7 | 7.8 | 127.0 | 126.1 | 127.9 | 46.4 | 43.9 | 48.9 |
| Assam | Female | 21.5 | 21.2 | 21.7 | 5.0 | 4.3 | 5.7 | 123.7 | 122.7 | 124.7 | 1.3 | 1.1 | 1.5 |
| Assam | Male | 21.5 | 21.3 | 21.8 | 6.1 | 5.2 | 7.0 | 128.1 | 127.2 | 128.9 | 29.4 | 26.3 | 32.6 |
| Bihar | Female | 21.0 | 20.8 | 21.2 | 3.2 | 2.7 | 3.7 | 123.6 | 122.6 | 124.6 | 2.7 | 2.1 | 3.3 |
| Bihar | Male | 20.6 | 20.5 | 20.8 | 4.3 | 3.7 | 5.0 | 125.2 | 124.5 | 125.9 | 22.5 | 20.4 | 24.7 |
| Chandigarh | Female | 24.5 | 24.1 | 24.8 | 15.0 | 12.2 | 18.0 | 129.1 | 127.6 | 130.7 | 0.3 | 0.1 | 0.6 |
| Chandigarh | Male | 23.9 | 23.5 | 24.2 | 14.6 | 12.2 | 17.2 | 133.8 | 132.5 | 135.0 | 18.7 | 13.2 | 24.9 |
| Chhattisgarh | Female | 21.2 | 21.0 | 21.4 | 5.0 | 4.2 | 5.8 | 123.4 | 122.3 | 124.5 | 0.4 | 0.3 | 0.6 |
| Chhattisgarh | Male | 21.6 | 21.4 | 21.8 | 7.2 | 6.0 | 8.5 | 127.0 | 126.1 | 127.8 | 15.1 | 13.0 | 17.3 |
| Daman and Diu | Female | 23.5 | 22.7 | 24.3 | 14.8 | 10.2 | 20.1 | 130.2 | 127.9 | 132.4 | 0.1 | 0.0 | 0.3 |
| Daman and Diu | Male | 24.1 | 23.4 | 24.7 | 10.5 | 6.3 | 15.6 | 132.3 | 130.2 | 134.5 | 9.9 | 6.2 | 14.4 |
| Goa | Female | 24.4 | 24.0 | 24.8 | 21.9 | 19.4 | 24.4 | 128.4 | 126.9 | 129.8 | 2.2 | 1.2 | 3.4 |
| Goa | Male | 24.0 | 23.7 | 24.3 | 24.8 | 22.0 | 27.7 | 131.4 | 129.6 | 133.2 | 9.3 | 7.1 | 11.8 |
| Haryana | Female | 23.3 | 23.2 | 23.4 | 8.1 | 7.6 | 8.6 | 123.7 | 123.3 | 124.0 | 2.2 | 1.9 | 2.5 |
| Haryana | Male | 23.0 | 22.9 | 23.1 | 8.3 | 7.8 | 8.9 | 127.6 | 127.3 | 128.0 | 35.3 | 34.0 | 36.6 |
| Himachal Pradesh | Female | 23.0 | 22.8 | 23.2 | 4.8 | 4.1 | 5.5 | 130.3 | 129.6 | 131.0 | 2.5 | 1.9 | 3.1 |
| Himachal Pradesh | Male | 23.0 | 22.9 | 23.2 | 4.4 | 3.8 | 5.1 | 132.7 | 132.0 | 133.5 | 41.7 | 39.1 | 44.3 |
| Jharkhand | Female | 21.2 | 20.9 | 21.5 | 4.5 | 3.8 | 5.3 | 122.3 | 121.5 | 123.2 | 0.7 | 0.4 | 1.0 |
| Jharkhand | Male | 21.6 | 21.4 | 21.8 | 5.9 | 5.0 | 6.9 | 126.6 | 125.6 | 127.6 | 9.9 | 8.2 | 11.7 |
| Karnataka | Female | 22.8 | 22.7 | 22.9 | 12.9 | 12.4 | 13.4 | 126.8 | 126.5 | 127.1 | 0.9 | 0.8 | 1.0 |
| Karnataka | Male | 22.9 | 22.8 | 22.9 | 13.8 | 13.2 | 14.4 | 128.6 | 128.3 | 128.9 | 21.4 | 20.5 | 22.2 |
| Kerala | Female | 24.4 | 24.2 | 24.6 | 15.1 | 14.0 | 16.1 | 129.6 | 128.7 | 130.5 | 0.8 | 0.6 | 1.1 |
| Kerala | Male | 23.9 | 23.7 | 24.2 | 18.3 | 16.9 | 19.8 | 132.3 | 131.2 | 133.4 | 30.4 | 28.2 | 32.7 |
| Madhya Pradesh | Female | 21.3 | 21.2 | 21.5 | 3.5 | 2.9 | 4.1 | 123.9 | 122.8 | 125.1 | 0.9 | 0.4 | 1.7 |
| Madhya Pradesh | Male | 21.1 | 20.9 | 21.2 | 4.0 | 3.5 | 4.5 | 126.2 | 125.5 | 126.9 | 25.4 | 23.8 | 27.0 |
| Maharashtra | Female | 22.3 | 22.2 | 22.4 | 7.4 | 7.0 | 7.8 | 123.7 | 123.4 | 123.9 | 0.5 | 0.4 | 0.6 |
| Maharashtra | Male | 22.7 | 22.6 | 22.8 | 8.3 | 7.8 | 8.7 | 126.2 | 126.0 | 126.5 | 9.4 | 8.8 | 10.0 |
| Manipur | Female | 22.9 | 22.8 | 23.1 | 9.6 | 8.7 | 10.5 | 123.5 | 122.9 | 124.1 | 12.6 | 10.8 | 14.5 |
| Manipur | Male | 22.6 | 22.4 | 22.7 | 10.2 | 9.3 | 11.2 | 128.1 | 127.5 | 128.7 | 60.3 | 57.9 | 62.7 |
| Meghalaya | Female | 21.8 | 21.6 | 22.0 | 3.8 | 3.0 | 4.7 | 125.2 | 124.3 | 126.1 | 5.8 | 4.7 | 7.1 |
| Meghalaya | Male | 22.1 | 21.9 | 22.3 | 4.3 | 3.3 | 5.4 | 127.7 | 126.5 | 128.9 | 59.7 | 56.2 | 63.3 |
| Mizoram | Female | 22.1 | 21.9 | 22.2 | 4.6 | 4.0 | 5.3 | 119.1 | 118.5 | 119.6 | 26.1 | 24.5 | 27.6 |
| Mizoram | Male | 22.3 | 22.1 | 22.4 | 4.8 | 4.2 | 5.5 | 125.7 | 125.2 | 126.3 | 71.7 | 70.1 | 73.2 |
| Nagaland | Female | 22.2 | 22.0 | 22.4 | 7.1 | 6.1 | 8.1 | 130.7 | 129.8 | 131.7 | 1.4 | 1.0 | 1.9 |
| Nagaland | Male | 22.3 | 22.1 | 22.5 | 7.5 | 6.6 | 8.5 | 133.6 | 132.6 | 134.5 | 42.1 | 39.6 | 44.6 |
| NCT of Delhi | Female | 24.0 | 23.7 | 24.2 | 15.3 | 14.0 | 16.7 | 124.8 | 124.2 | 125.4 | 1.8 | 1.3 | 2.4 |
| NCT of Delhi | Male | 23.7 | 23.6 | 23.9 | 14.4 | 13.3 | 15.6 | 127.1 | 126.6 | 127.7 | 27.8 | 25.6 | 30.0 |
| Odisha | Female | 21.4 | 21.2 | 21.6 | 4.2 | 3.9 | 4.7 | 120.0 | 119.2 | 120.9 | 0.9 | 0.7 | 1.1 |
| Odisha | Male | 21.4 | 21.2 | 21.6 | 5.6 | 5.1 | 6.2 | 121.7 | 120.7 | 122.6 | 19.6 | 18.1 | 21.1 |
| Puducherry | Female | 25.1 | 24.8 | 25.4 | 20.7 | 19.1 | 22.3 | 123.9 | 123.3 | 124.6 | 0.3 | 0.1 | 0.5 |
| Puducherry | Male | 24.2 | 23.9 | 24.4 | 22.2 | 20.5 | 24.1 | 128.2 | 127.3 | 129.1 | 14.0 | 12.1 | 16.0 |
| Punjab | Female | 25.1 | 25.0 | 25.1 | 10.9 | 10.4 | 11.3 | 131.2 | 130.9 | 131.5 | 0.3 | 0.2 | 0.4 |
| Punjab | Male | 24.3 | 24.2 | 24.4 | 10.4 | 9.9 | 10.9 | 136.2 | 135.9 | 136.6 | 15.9 | 15.0 | 16.8 |
| Rajasthan | Female | 21.5 | 21.3 | 21.7 | 3.7 | 3.3 | 4.2 | 121.4 | 120.8 | 122.1 | 2.2 | 1.6 | 2.8 |
| Rajasthan | Male | 21.3 | 21.1 | 21.5 | 4.5 | 3.9 | 5.0 | 126.1 | 125.4 | 126.7 | 23.6 | 20.9 | 26.5 |
| Sikkim | Female | 24.5 | 24.2 | 24.8 | 7.5 | 6.4 | 8.6 | 132.8 | 131.5 | 134.0 | 6.0 | 4.9 | 7.2 |
| Sikkim | Male | 23.6 | 23.3 | 23.8 | 7.4 | 6.4 | 8.6 | 133.1 | 132.0 | 134.2 | 23.6 | 21.2 | 26.1 |
| Tamil Nadu | Female | 24.0 | 23.9 | 24.2 | 20.1 | 19.5 | 20.7 | 124.3 | 124.0 | 124.6 | 0.5 | 0.4 | 0.5 |
| Tamil Nadu | Male | 23.3 | 23.2 | 23.3 | 21.2 | 20.6 | 21.8 | 128.3 | 128.1 | 128.6 | 19.4 | 18.6 | 20.2 |
| Telangana | Female | 22.4 | 22.2 | 22.7 | 9.9 | 9.0 | 10.9 | 122.3 | 121.6 | 122.9 | 2.9 | 2.2 | 3.6 |
| Telangana | Male | 22.9 | 22.7 | 23.1 | 11.3 | 10.3 | 12.4 | 125.2 | 124.5 | 125.9 | 34.7 | 32.5 | 37.0 |
| Tripura | Female | 21.8 | 21.6 | 22.1 | 14.8 | 12.8 | 17.0 | 125.7 | 124.5 | 126.9 | 5.6 | 3.9 | 7.6 |
| Tripura | Male | 21.8 | 21.5 | 22.0 | 15.7 | 13.4 | 18.2 | 127.1 | 125.8 | 128.4 | 39.3 | 34.8 | 43.9 |
| Uttar Pradesh | Female | 21.7 | 21.5 | 21.8 | 4.7 | 4.2 | 5.2 | 122.9 | 122.1 | 123.6 | 2.5 | 2.1 | 2.9 |
| Uttar Pradesh | Male | 20.8 | 20.6 | 21.0 | 5.2 | 4.6 | 5.8 | 125.0 | 124.1 | 125.9 | 32.9 | 31.0 | 34.7 |
| Uttarakhand | Female | 23.4 | 22.9 | 23.8 | 6.3 | 4.7 | 8.0 | 125.8 | 124.5 | 127.2 | 1.6 | 1.1 | 2.3 |
| Uttarakhand | Male | 22.8 | 22.5 | 23.1 | 6.9 | 5.4 | 8.6 | 131.7 | 130.3 | 133.2 | 26.6 | 22.6 | 30.9 |
| West Bengal | Female | 21.7 | 21.6 | 21.8 | 11.6 | 10.9 | 12.4 | 127.0 | 126.4 | 127.5 | 3.3 | 2.8 | 3.9 |
| West Bengal | Male | 21.7 | 21.6 | 21.8 | 12.8 | 12.0 | 13.6 | 126.9 | 126.4 | 127.5 | 49.5 | 48.0 | 51.0 |

**Abbreviations:** CI=95% confidence interval

**Table G. Multivariable linear regression of the natural logarithm of 10-year CVD risk (as calculated with the Harvard-NHANES score) on district-level wealth quintile and individual-level socio-demographic characteristics***^3,4,5,6,7,8^*

|  | **Rural areas** | | **Urban areas** | |
| --- | --- | --- | --- | --- |
|  | *Coefficient*  *(95% CI)* | *P* | *Coefficient*  *(95% CI)* | *P* |
| District wealth quintile |  |  |  |  |
| 1 (poorest) | Ref. |  | Ref. |  |
| 2 | 2.16 (0.87 - 3.45) | 0.001 | 2.93 (1.12 - 4.75) | 0.002 |
| 3 | 6.17 (4.29 - 8.04) | <0.001 | 1.50 (-0.54 - 3.54) | 0.149 |
| 4 | 4.66 (2.78 - 6.54) | <0.001 | 3.02 (0.98 - 5.07) | 0.004 |
| 5 (richest) | 10.71 (8.67 - 12.75) | <0.001 | 3.36 (0.91 - 5.81) | 0.007 |
| Household wealth quintile |  |  |  |  |
| 1 (poorest) | Ref. |  | Ref. |  |
| 2 | 0.75 (0.43 - 1.06) | <0.001 | 4.34 (3.83 - 4.85) | <0.001 |
| 3 | 2.39 (2.04 - 2.74) | <0.001 | 8.79 (8.26 - 9.33) | <0.001 |
| 4 | 5.41 (5.04 - 5.78) | <0.001 | 11.82 (11.26 - 12.38) | <0.001 |
| 5 (richest) | 11.92 (11.50 - 12.34) | <0.001 | 14.02 (13.41 - 14.63) | <0.001 |
| Educational attainment |  |  |  |  |
| <Primary School | Ref. |  | Ref. |  |
| Primary School | 1.55 (1.24 - 1.87) | <0.001 | 2.62 (2.08 - 3.17) | <0.001 |
| Middle School | 1.75 (1.43 - 2.08) | <0.001 | 2.66 (2.14 - 3.18) | <0.001 |
| Secondary School | 2.54 (2.16 - 2.91) | <0.001 | 2.89 (2.37 - 3.40) | <0.001 |
| High School | 1.84 (1.33 - 2.34) | <0.001 | 1.36 (0.74 - 1.98) | <0.001 |
| >High School | 2.29 (1.72 - 2.85) | <0.001 | 0.73 (0.15 - 1.30) | 0.013 |

**Abbreviations:** CI=Confidence Interval; Ref. = Reference category.

^3^ The model included random intercepts by district.

^4^ This model included all variables listed in the table, five-year age groups, and a binary indicator for sex as explanatory variables.

^5^ 10-year cardiovascular disease risk was calculated using the Harvard-NHANES score.

^6^ The dataset was first divided into participants living in rural versus urban areas before district wealth quintile was calculated and the regression model was fitted.

^7^ District wealth quintile was calculated, separately for rural and urban areas within districts, by computing the median of the continuous household wealth index in a district and then categorizing the district-level median into quintiles.

^8^ Coefficients were multiplied by 100 so that they can be interpreted as an approximation of the percentage change in cardiovascular risk associated with a one unit change in the explanatory variable.

**Table H. Multivariable linear regression of the natural logarithm of 10-year CVD risk (as calculated with the Harvard-NHANES score) on the proportion of participants in a district who live in an urban area***^5,6,11,7,10^*

|  | **Coefficient**  **(95% CI)** | **P** |
| --- | --- | --- |
| District-level proportion living in an urban area | 16.43 (12.95 - 19.91) | <0.001 |
| Household wealth quintile |  |  |
| 1 (poorest) | Ref. |  |
| 2 | 0.96 (0.69 - 1.23) | <0.001 |
| 3 | 2.40 (2.11 - 2.69) | <0.001 |
| 4 | 4.43 (4.13 - 4.73) | <0.001 |
| 5 (richest) | 8.47 (8.14 - 8.81) | <0.001 |
| Educational attainment |  |  |
| <Primary School | Ref. |  |
| Primary School | 3.76 (3.49 - 4.04) | <0.001 |
| Middle School | 4.75 (4.47 - 5.02) | <0.001 |
| Secondary School | 6.71 (6.41 - 7.00) | <0.001 |
| High School | 6.30 (5.92 - 6.68) | <0.001 |
| >High School | 7.30 (6.94 - 7.65) | <0.001 |

**Abbreviations:** CI=Confidence Interval; Ref. = Reference category.

^3^ The model included random intercepts by district.

^4^ This model included all variables listed in the table, five-year age groups, and a binary indicator for sex as explanatory variables.

^9^ ‘District-level proportion living in an urban area’ refers to the proportion (between 0 and 1) of the participants in a district who live in an urban area.

^8^ Coefficients were multiplied by 100 so that they can be interpreted as an approximation of the percentage change in cardiovascular risk associated with a one unit change in the explanatory variable.

**Table I. Multivariable linear regression of the natural logarithm of 10-year CVD risk (as calculated with Globorisk) on district-level wealth quintile and individual-level socio-demographic characteristics***^3,4,6,7,8^*

|  | **Rural areas** | | **Urban areas** | |
| --- | --- | --- | --- | --- |
|  | *Coefficient*  *(95% CI)* | *P* | *Coefficient*  *(95% CI)* | *P* |
| District wealth quintile |  |  |  |  |
| 1 (poorest) | Ref. |  | Ref. |  |
| 2 | 2.12 (0.31 - 3.94) | 0.022 | 3.69 (1.36 - 6.03) | 0.002 |
| 3 | 6.68 (4.25 - 9.11) | <0.001 | 1.59 (-0.94 - 4.11) | 0.219 |
| 4 | 5.45 (3.01 - 7.89) | <0.001 | 3.26 (0.72 - 5.79) | 0.012 |
| 5 (richest) | 11.99 (9.38 - 14.60) | <0.001 | 5.43 (2.43 - 8.43) | <0.001 |
| Household wealth quintile |  |  |  |  |
| 1 (poorest) | Ref. |  | Ref. |  |
| 2 | -0.08 (-0.56 - 0.40) | 0.749 | 3.44 (2.70 - 4.18) | <0.001 |
| 3 | 0.99 (0.46 - 1.52) | <0.001 | 7.19 (6.42 - 7.95) | <0.001 |
| 4 | 3.16 (2.60 - 3.71) | <0.001 | 9.09 (8.30 - 9.88) | <0.001 |
| 5 (richest) | 8.63 (8.00 - 9.26) | <0.001 | 10.26 (9.40 - 11.12) | <0.001 |
| Educational attainment |  |  |  |  |
| <Primary School | Ref. |  | Ref. |  |
| Primary School | 1.50 (1.02 - 1.98) | <0.001 | 1.52 (0.77 - 2.26) | <0.001 |
| Middle School | 2.45 (1.93 - 2.97) | <0.001 | 1.39 (0.65- 2.12) | <0.001 |
| Secondary School | 4.29 (3.69 - 4.89) | <0.001 | 1.38 (0.66 - 2.10) | <0.001 |
| High School | 5.06 (4.19 - 5.94) | <0.001 | -0.02 (-0.93 - 0.88) | 0.959 |
| >High School | 6.22 (5.28 - 7.16) | <0.001 | -0.26 (-1.07 - 0.56) | 0.539 |

**Abbreviations:** CI=Confidence Interval; Ref. = Reference category.

^3^ The model included random intercepts by district.

^4^ This model included all variables listed in the table, five-year age groups, and a binary indicator for sex as explanatory variables.

^6^ The dataset was first divided into participants living in rural versus urban areas before district wealth quintile was calculated and the regression model was fitted.

^7^ District wealth quintile was calculated, separately for rural and urban areas within districts, by computing the median of the continuous household wealth index in a district and then categorizing the district-level median into quintiles.

^8^ Coefficients were multiplied by 100 so that they can be interpreted as an approximation of the percentage change in cardiovascular risk associated with a one unit change in the explanatory variable.

**Table J. Multivariable linear regression of the natural logarithm of 10-year CVD risk (as calculated with Globorisk) on the proportion of participants in a district who live in an urban area***^3,4,9,8^*

|  | **Coefficient**  **(95% CI)** | **P** |
| --- | --- | --- |
| District-level proportion living in an urban area | 12.71 (8.39 - 17.03) | <0.001 |
| Household wealth quintile |  |  |
| 1 (poorest) | Ref. |  |
| 2 | 0.17 (-0.24 - 0.57) | 0.421 |
| 3 | 1.16 (0.73 - 1.58) | <0.001 |
| 4 | 2.44 (1.99 - 2.88) | <0.001 |
| 5 (richest) | 5.65 (5.16 - 6.14) | <0.001 |
| Educational attainment |  |  |
| <Primary School | Ref. |  |
| Primary School | 3.36 (2.96 - 3.76) | <0.001 |
| Middle School | 4.70 (4.28 - 5.12) | <0.001 |
| Secondary School | 6.98 (6.53 - 7.42) | <0.001 |
| High School | 7.13 (6.53 - 7.73) | <0.001 |
| >High School | 7.93 (7.38 - 8.48) | <0.001 |

**Abbreviations:** CI=Confidence Interval; Ref. = Reference category.

^3^ The model included random intercepts by district.

^4^ This model included all variables listed in the table, five-year age groups, and a binary indicator for sex as explanatory variables.

^9^ ‘District-level proportion living in an urban area’ refers to the proportion (between 0 and 1) of the participants in a district who live in an urban area.

^8^ Coefficients were multiplied by 100 so that they can be interpreted as an approximation of the percentage change in cardiovascular risk associated with a one unit change in the explanatory variable.

**Table K. Ordinary least squares regressions of the natural logarithm of the 10-year Harvard-NHANES risk score on sociodemographic covariates and district-level fixed effects**^10,8^

|  | **Female (n=419,478)** | | | | **Male (n=375,642)** | | | |
| --- | --- | --- | --- | --- | --- | --- | --- | --- |
|  | **Adjusted for age group only^11^** | | **Adjusted for all covariates^12^** | | **Adjusted for age group only^11^** | | **Adjusted for all covariates^12^** | |
|  | *Coefficient*  *(95% CI)* | *P* | *Coefficient*  *(95% CI)* | *P* | *Coefficient*  *(95% CI)* | *P* | *Coefficient*  *(95% CI)* | *P* |
| Wealth quintile |  |  |  |  |  |  |  |  |
| 1 (poorest) | Ref. |  | Ref. |  | Ref. |  | Ref. |  |
| 2 | 2.73 (2.36 - 3.09) | <0.001 | 2.88 (2.52 - 3.25) | <0.001 | 0.85 (0.46 - 1.24) | <0.001 | 0.97 (0.58 - 1.36) | <0.001 |
| 3 | 5.44 (5.05 - 5.83) | <0.001 | 5.95 (5.56 - 6.34) | <0.001 | 2.30 (1.89 - 2.71) | <0.001 | 2.64 (2.23 - 3.06) | <0.001 |
| 4 | 8.81 (8.41 - 9.21) | <0.001 | 9.41 (9.00 - 9.82) | <0.001 | 4.39 (3.97 - 4.81) | <0.001 | 4.83 (4.39 - 5.27) | <0.001 |
| 5 (richest) | 15.19 (14.76 - 15.62) | <0.001 | 15.71 (15.25 - 16.18) | <0.001 | 8.48 (8.03 - 8.92) | <0.001 | 8.87 (8.38 - 9.37) | <0.001 |
| Educational attainment |  |  |  |  |  |  |  |  |
| <Primary School | Ref. |  | Ref. |  | Ref. |  | Ref. |  |
| Primary School | 7.58 (7.21 - 7.96) | <0.001 | 4.18 (3.80 - 4.55) | <0.001 | 2.26 (1.87 - 2.65) | <0.001 | 1.09 (0.70 - 1.48) | <0.001 |
| Middle School | 9.55 (9.17 - 9.93) | <0.001 | 4.50 (4.11 - 4.89) | <0.001 | 3.12 (2.75 - 3.50) | <0.001 | 1.19 (0.81 - 1.57) | <0.001 |
| Secondary School | 12.83 (12.41 - 13.26) | <0.001 | 5.33 (4.89 - 5.77) | <0.001 | 5.98 (5.60 - 6.36) | <0.001 | 2.75 (2.35 - 3.15) | <0.001 |
| High School | 12.72 (12.15 - 13.29) | <0.001 | 3.54 (2.95 - 4.14) | <0.001 | 5.34 (4.87 - 5.81) | <0.001 | 1.15 (0.65 - 1.64) | <0.001 |
| >High School | 13.65 (13.10 - 14.20) | <0.001 | 1.55 (0.95 - 2.14) | <0.001 | 8.05 (7.62 - 8.48) | <0.001 | 1.89 (1.41 - 2.38) | <0.001 |
| Geography |  |  |  |  |  |  |  |  |
| Rural | Ref. |  | Ref. |  | Ref. |  | Ref. |  |
| Urban | 12.60 (12.34 - 12.87) | <0.001 | 12.92 (12.63 - 13.21) | <0.001 | 7.16 (6.88 - 7.44) | <0.001 | 7.46 (7.16 - 7.76) | <0.001 |

**Abbreviations:** CI=Confidence Interval; Ref. = Reference category.

^10^ Standard errors were adjusted for clustering at the level of a primary sampling unit.

^11^ These models included one sociodemographic characteristic, age group, and a binary indicator variable for each district as explanatory variables.

^12^ This model included all variables listed in the table, age group, and a binary indicator for each district as explanatory variables.

^8^ Coefficients were multiplied by 100 so that they can be interpreted as an approximation of the percentage change in cardiovascular risk associated with a one unit change in the explanatory variable.

**Table L. Ordinary least squares regressions of the natural logarithm of the 10-year Globorisk score on sociodemographic covariates and district-level fixed effects**^10,8^

|  | **Female (n=276,318)** | | | | **Male (n=** **261,736)** | | | |
| --- | --- | --- | --- | --- | --- | --- | --- | --- |
|  | **Adjusted for age group only^11^** | | **Adjusted for all covariates^12^** | | **Adjusted for age group only^11^** | | **Adjusted for all covariates^12^** | |
|  | *Coefficient*  *(95% CI)* | *P* | *Coefficient*  *(95% CI)* | *P* | *Coefficient*  *(95% CI)* | *P* | *Coefficient*  *(95% CI)* | *P* |
| Wealth quintile |  |  |  |  |  |  |  |  |
| 1 (poorest) | Ref. |  | Ref. |  | Ref. |  | Ref. |  |
| 2 | 1.67 (1.13 - 2.21) | <0.001 | 1.76 (1.22 - 2.30) | <0.001 | 0.96 (0.42 - 1.51) | <0.001 | 1.00 (0.46 - 1.55) | <0.001 |
| 3 | 3.89 (3.32 - 4.46) | <0.001 | 4.15 (3.58 - 4.73) | <0.001 | 2.38 (1.81 - 2.96) | <0.001 | 2.56 (1.97 - 3.14) | <0.001 |
| 4 | 6.49 (5.90 - 7.07) | <0.001 | 6.68 (6.08 - 7.27) | <0.001 | 4.17 (3.58 - 4.76) | <0.001 | 4.37 (3.75 - 4.98) | <0.001 |
| 5 (richest) | 12.19 (11.57 - 12.81) | <0.001 | 11.98 (11.31 - 12.64) | <0.001 | 8.05 (7.43 - 8.68) | <0.001 | 8.08 (7.39 - 8.77) | <0.001 |
| Educational attainment |  |  |  |  |  |  |  |  |
| <Primary School | Ref. |  | Ref. |  | Ref. |  | Ref. |  |
| Primary School | 7.29 (6.74 - 7.84) | <0.001 | 4.11 (3.55 - 4.67) | <0.001 | 2.62 (2.09 - 3.15) | <0.001 | 1.43 (0.90 - 1.97) | <0.001 |
| Middle School | 9.42 (8.83 - 10.02) | <0.001 | 4.78 (4.17 - 5.40) | <0.001 | 3.42 (2.90 - 3.95) | <0.001 | 1.50 (0.96 - 2.03) | <0.001 |
| Secondary School | 12.47 (11.81 - 13.14) | <0.001 | 5.61 (4.90 - 6.31) | <0.001 | 6.56 (6.02 - 7.09) | <0.001 | 3.33 (2.77 - 3.89) | <0.001 |
| High School | 12.75 (11.77 - 13.73) | <0.001 | 4.64 (3.62 - 5.66) | <0.001 | 5.04 (4.35 - 5.74) | <0.001 | 0.87 (0.14 - 1.60) | 0.019 |
| >High School | 12.58 (11.67 - 13.50) | <0.001 | 2.33 (1.35 - 3.30) | <0.001 | 7.89 (7.28 - 8.50) | <0.001 | 1.88 (1.19 - 2.57) | <0.001 |
| Geography |  |  |  |  |  |  |  |  |
| Rural | Ref. |  | Ref. |  | Ref. |  | Ref. |  |
| Urban | 11.82 (11.43 - 12.22) | <0.001 | 11.43 (11.01 - 11.85) | <0.001 | 7.25 (6.85 - 7.65) | <0.001 | 7.28 (6.86 - 7.71) | <0.001 |

**Abbreviations:** CI=Confidence Interval; Ref. = Reference category.

^10^ Standard errors were adjusted for clustering at the level of a primary sampling unit.

^11^ These models included one sociodemographic characteristic, age group, and a binary indicator variable for each district as explanatory variables.

^12^ This model included all variables listed in the table, age group, and a binary indicator for each district as explanatory variables.

^8^ Coefficients were multiplied by 100 so that they can be interpreted as an approximation of the percentage change in cardiovascular risk associated with a one unit change in the explanatory variable.
