# Supplementary material for: Geographic and sociodemographic variation of cardiovascular disease risk in India: A cross-sectional study of 797,540 adults
Source: PLoS Med. 2018 Jun 19;15(6):e1002581. doi: 10.1371/journal.pmed.1002581 (PMC6007838; doi:10.1371/journal.pmed.1002581)
Supplement: S1 Text — (DOCX) [file pmed.1002581.s004.docx]

**Text A. Matching AHS biomarker data to participants’ sociodemographic data**

AHS data in the public domain does not have a unique identifier that allows for merging of the ‘laboratory dataset’, which contains height, weight, blood pressure, and blood glucose measurements, to the dataset that contains respondents’ full sociodemographic and treatment information. We thus merged these datasets using an indicator composed of the state, district, stratum (indicating rural versus urban location and village size), a household identifier that is unique within each PSU, and a household member serial number given during data entry as well as one assigned after data entry.

415,728 out of 661,141 (62.9%) non-pregnant adults aged 30 to 74 years in the laboratory dataset were successfully matched to their corresponding sociodemographic and treatment information. As detailed in the tables below, participants who were not matched had similar characteristics as those who were matched.

***Across all nine AHS states:***

| **Variable** | **Matched** | **Not matched** |
| --- | --- | --- |
|  | *n= 415,728* | *n=245,413* |
| Male (%) | 49.8 | 49.0 |
| Age (mean ± SD) | 46.5±11.7 | 47.1±12.3 |
| Glucose (mean ± SD) | 99.0±21.3 | 99.2±21.6 |
| Diabetes (%) | 4.6 | 4.6 |
| Systolic BP (mean ± SD) | 125.4±19.3 | 125.6±19.9 |
| BMI (mean ± SD) | 21.2±3.7 | 21.2±3.7 |
| Urban (%) | 19.3 | 18.3 |

**Abbreviations:** SD=standard deviation

***Assam:***

| **Variable** | **Matched** | **Not matched** |
| --- | --- | --- |
|  | *n=44,479* | *n=13,132* |
| Male (%) | 51.3 | 47.1 |
| Age (mean ± SD) | 45.4±11.2 | 45.6±12.0 |
| Glucose (mean ± SD) | 99.8±21.0 | 99.8±21.2 |
| Diabetes (%) | 5.2 | 5.4 |
| Systolic BP (mean ± SD) | 128.1±18.0 | 127.2±18.4 |
| BMI (mean ± SD) | 21.5±3.3 | 21.1±3.5 |
| Urban (%) | 17.1 | 23.1 |

**Abbreviations:** SD=standard deviation

***Bihar:***

| **Variable** | **Matched** | **Not matched** |
| --- | --- | --- |
|  | *n=49,213* | *n=45,723* |
| Male (%) | 48.4 | 53.5 |
| Age (mean ± SD) | 46.4±11.8 | 46.6±12.3 |
| Glucose (mean ± SD) | 97.0±18.9 | 95.2±18.4 |
| Diabetes (%) | 3.9 | 3.4 |
| Systolic BP (mean ± SD) | 125.4±19.0 | 123.8±18.3 |
| BMI (mean ± SD) | 20.7±3.1 | 20.9±3.1 |
| Urban (%) | 9.9 | 8.3 |

**Abbreviations:** SD=standard deviation

***Chhattisgarh:***

| **Variable** | **Matched** | **Not matched** |
| --- | --- | --- |
|  | *n=26,035* | *n= 12,185* |
| Male (%) | 51.0 | 49.2 |
| Age (mean ± SD) | 45.8±11.3 | 46.5±12.0 |
| Glucose (mean ± SD) | 100.5±18.2 | 101.2±21.1 |
| Diabetes (%) | 5.5 | 6.6 |
| Systolic BP (mean ± SD) | 125.2±17.1 | 126.1±17.8 |
| BMI (mean ± SD) | 21.2±3.3 | 21.3±3.4 |
| Urban (%) | 19.4 | 23.2 |

**Abbreviations:** SD=standard deviation

***Jharkhand:***

| **Variable** | **Matched** | **Not matched** |
| --- | --- | --- |
|  | *n=24,645* | *n=11,255* |
| Male (%) | 44.5 | 45.0 |
| Age (mean ± SD) | 46.3±11.7 | 47.3±12.3 |
| Glucose (mean ± SD) | 96.9±24.9 | 96.6±22.5 |
| Diabetes (%) | 5.1 | 4.7 |
| Systolic BP (mean ± SD) | 125.6±20.7 | 126.7±21.0 |
| BMI (mean ± SD) | 21.2±4.1 | 21.2±3.9 |
| Urban (%) | 17.5 | 22.7 |

**Abbreviations:** SD=standard deviation

***Madhya Pradesh:***

| **Variable** | **Matched** | **Not matched** |
| --- | --- | --- |
|  | *n=67,506* | *n=37,172* |
| Male (%) | 53.2 | 52.1 |
| Age (mean ± SD) | 46.0±11.7 | 46.7±12.2 |
| Glucose (mean ± SD) | 97.9±19.2 | 98.0±18.6 |
| Diabetes (%) | 3.7 | 3.5 |
| Systolic BP (mean ± SD) | 126.3±18.2 | 126.7±17.9 |
| BMI (mean ± SD) | 21.2±3.2 | 21.2±3.3 |
| Urban (%) | 32.6 | 29.2 |

**Abbreviations:** SD=standard deviation

***Odisha:***

| **Variable** | **Matched** | **Not matched** |
| --- | --- | --- |
|  | *n=65,511* | *n=12,034* |
| Male (%) | 49.2 | 45.6 |
| Age (mean ± SD) | 47.0±11.8 | 47.9±12.3 |
| Glucose (mean ± SD) | 98.7±23.5 | 99.0±24.5 |
| Diabetes (%) | 5.0 | 5.2 |
| Systolic BP (mean ± SD) | 122.7±20.2 | 123.4±20.7 |
| BMI (mean ± SD) | 21.2±4.0 | 21.3±4.1 |
| Urban (%) | 14.0 | 13.8 |

**Abbreviations:** SD=standard deviation

***Rajasthan:***

| **Variable** | **Matched** | **Not matched** |
| --- | --- | --- |
|  | *n=55,928* | *n=13,682* |
| Male (%) | 47.8 | 46.3 |
| Age (mean ± SD) | 46.6±11.7 | 47.3±12.5 |
| Glucose (mean ± SD) | 100.1±20.1 | 100.2±19.9 |
| Diabetes (%) | 4.4 | 4.3 |
| Systolic BP (mean ± SD) | 124.8±19.0 | 125.4±19.5 |
| BMI (mean ± SD) | 21.4±4.0 | 21.4±4.1 |
| Urban (%) | 17.6 | 17.7 |

**Abbreviations:** SD=standard deviation

***Uttar Pradesh:***

| **Variable** | **Matched** | **Not matched** |
| --- | --- | --- |
|  | *n=68,497* | *n=88,121* |
| Male (%) | 50.7 | 47.9 |
| Age (mean ± SD) | 47.0±11.9 | 47.6±12.5 |
| Glucose (mean ± SD) | 100.9±22.0 | 101.1±22.9 |
| Diabetes (%) | 4.8 | 4.9 |
| Systolic BP (mean ± SD) | 124.9±20.6 | 125.8±21.4 |
| BMI (mean ± SD) | 21.2±3.9 | 21.0±3.9 |
| Urban (%) | 20.7 | 17.3 |

**Abbreviations:** SD=standard deviation

***Uttarakhand:***

| **Variable** | **Matched** | **Not matched** |
| --- | --- | --- |
|  | *n=13,914* | *n=12,109* |
| Male (%) | 46.3 | 42.2 |
| Age (mean ± SD) | 48.0±12.1 | 47.7±12.3 |
| Glucose (mean ± SD) | 100.1±24.4 | 103.2±26.8 |
| Diabetes (%) | 5.6 | 5.6 |
| Systolic BP (mean ± SD) | 129.6±19.7 | 127.2±20.9 |
| BMI (mean ± SD) | 22.9±4.0 | 22.7±4.3 |
| Urban (%) | 21.7 | 21.2 |

**Abbreviations:** SD=standard deviation
